# Optimising fundoscopy practices across the medical spectrum: A focus group study

**DOI:** 10.1371/journal.pone.0280937

**Published:** 2023-01-27

**Authors:** Hamish P. Dunn, Christine J. Kang, Samuel Marks, Stewart M. Dunn, Paul R. Healey, Andrew J. White

**Affiliations:** 1 Rural Clinical School, Faculty of Medicine, University of New South Wales, Sydney, NSW, Australia; 2 Faculty of Medicine & Health, University of Sydney, Sydney, NSW, Australia; 3 Department of Ophthalmology, Westmead Hospital, Sydney, NSW, Australia; 4 Department of Ophthalmology, Port Macquarie Base Hospital, Port Macquarie, NSW, Australia; 5 The Westmead Institute for Medical Research, Westmead Hospital, Westmead, NSW, Australia; 6 The Pam McClean Cancer Communications Centre, Sydney, NSW, Australia; 7 Faculty of Medicine, University of New South Wales, Sydney, NSW, Australia; Public Library of Science, UNITED STATES

## Abstract

**Introduction:**

Fundoscopy can be of great clinical value, yet remains underutilised. Educational attempts to improve fundoscopy utilisation have had limited success. We aimed to explore the barriers and facilitators underlying the uptake of clinical direct ophthalmoscopy across a spectrum of medical specialties and training levels.

**Methods:**

Ten focus groups were conducted with medical students (n = 42), emergency department doctors (n = 24), basic physician trainees (n = 7), hospital physicians (n = 6) and general practitioners (n = 7). Independent thematic analysis of transcripts was conducted by three investigators. A consensus thematic framework was developed, and transcripts were reanalysed using this framework.

**Results:**

Thematic analysis identified seven main themes: (1) technical barriers to performing fundoscopy examinations; (2) clinical culture and expectations regarding fundoscopy; (3) the influence of fundoscopy on clinical management; (4) motivation to perform the examination; (5) novel technology including smartphone fundoscopy, and the value of a digital fundus image; (6) training requirements, and; (7) use of limited resources.

**Conclusion:**

Our results build a more nuanced picture of the factors which determine fundoscopy utilisation. As current barriers limit practice by clinicians and medical students, expertise and confidence performing and interpreting fundoscopy are lost. This shifts the balance of perceived clinical utility to futility in changing patient management, and reinforces a cycle of reducing fundoscopy utilisation. We identified important cultural barriers such as accepted incompetence, and misperceptions of senior discouragement. Emerging technologies reduce the technical barriers to fundoscopy. Therefore education should: focus on detecting pathology from digital images; clarify the role of fundoscopy in patient management, and; be targeted at key career progression points.

## Introduction

Fundoscopy offers a non-invasive view of neurovascular tissues relevant to multitudinous clinical presentations, yet it is neglected in routine clinical practice [1]. Reported fundoscopy rates amongst patients whose presentation warrants this examination are universally poor at just: 3% by hospital physicians [2]; 14% amongst Emergency Department (ED) specialists during a known trial on fundoscopy [3]; 18% amongst acute medical presentations [4]; and 34% by community physicians examining patients with newly diagnosed type 2 diabetes [5].

The International Council of Ophthalmology ranked fundoscopy amongst seven basic ophthalmic competencies for medical training [6]. However, the technical challenges of traditional direct ophthalmoscope (TDO) examination and subsequent interpretation of findings are reported barriers to fundus examination [1, 2]. The consequent diagnostic inaccuracy of TDO has fuelled disagreements about the capabilities expected of medical graduates [7].

There has been progress in the ease of fundoscopy, with emerging technologies including smartphone adaptors and increasingly portable non-mydriatic cameras [8, 9]. However increasing fundoscopy utilisation has been more elusive [10]. The ToTEMS study found medical students were more accurate in identifying fundus pathology using fundus photographs. However the following year students’ median frequency of fundoscopy on patients was only 10%. Reported barriers to fundoscopy included discomfort with the examination (38%), discouragement by supervisors (20%), and insufficient time (15%) [11].

Clinician behaviours and their choices to employ clinical examinations are complex and multifactorial [12]. Despite the emergence of novel technologies and fundoscopy education tools, reported rates of fundoscopy remain low. Existing explanations for the decline of fundoscopy have been proposed on the basis of expert opinion, and surveys. However, there remains a lack of qualitative ophthalmology research in general [13], and in particular examining why fundoscopy is so infrequently performed, and how this may be ameliorated. A deeper exploration is warranted to underpin more effective fundoscopy education, and thus ensure that doctors have access to valuable ocular fundus findings, and patients experience the best clinical outcomes. Therefore this study aimed to explore the barriers and facilitators of fundoscopy across a spectrum of medical training and specialties.

## Methods

This study adhered to the Declaration of Helsinki and Ethics Committee approval was obtained. Written informed participant consent was obtained.

### Design, setting & participants

Focus group participants for this qualitative study were recruited from two major metropolitan teaching hospitals with affiliated medical schools and two General Practice (GP) locations in northern and south-western Sydney. These were purposely selected for differing patient catchment socio-economic and ethnic demographics. Second- and Final-Year Medical Students were recruited by advertisement on their University online forum and an email from clinical administration. Doctors in training in Emergency Medicine and Basic Physician Trainees received an email offer from their Directors of Clinical Training. Consultant Emergency Medicine physicians and internal medicine physicians were recruited by word of mouth referral by relevant Directors of Clinical Training. Focus groups for Medical Students were run after OSCE sessions involving a crossover trial of smartphone and TDO [14]. Specialty trainee sessions were run after scheduled ophthalmology education using smartphones and TDO, to improve recruitment. All sessions were held in education rooms routinely used by participants.

Focus groups were facilitated by authors with formal training in facilitation and qualitative research (HD, SD), and whenever possible by an author who was not an ophthalmologist (SD) to minimise bias. A focus group prompt guide was developed by synthesising questions from the literature regarding fundoscopy utilisation (S1 Appendix). This guide was iteratively modified after successive sessions, incorporating data from participant groups following an inductive grounded theory approach [15]. In this theoretical sampling method, questions could be modified to test interpretations and build emerging theories. Amongst each participant group, focus groups were run until thematic saturation, when no new themes arose in successive sessions. Focus groups were audio-recorded and transcribed with de-identification.

### Analysis

Reporting and analysis was conducted according to the Standards for Reporting Qualitative Research [16]. NVivo 11.0 was used to code and analyse transcripts. A constructivist grounded theory approach was used to develop a theoretical explanation of the emerging themes using an iterative process of returning to qualitative data to refine theories [15]. Grounded theory uses an inductive rather than deductive approach to data analysis, and was selected as the underperformance of fundoscopy has not previously been studied using in-depth qualitative analysis, and existing theories are unlikely to completely explain its poor uptake. Thematic analysis open and axial coding of three transcripts was performed independently by three investigators (HD, SD, CK) who then met to develop a consensus thematic framework. Open coding involved breaking transcripts into discrete data components and applying a descriptive label. Axial coding involved looking for connections between codes, and condensing them into broader categories. This initial consensus framework involved the generation of axial coding frameworks and agreements on the codes to be used. All transcripts were re-coded using this framework by one author (CK), and verified by a second author (HD). During this process open and axial codes were iteratively refined then selective coding was used to: determine overarching categories; identify thematic interconnections between the overarching category and other codes, and; remove categories without sufficient supporting data. Three investigators (HD, SD, CK) then reconvened to refine the consensus selective coding framework. Our research team had expertise in clinical and research ophthalmology (HD, AW, PH), medical education (SD, HD), clinical medicine (CK, HD, AW, PH), biomedical engineering and computer science (SM), and qualitative research (SD, HD). The final thematic coding framework was distributed to the research team to facilitate reflective discussion and interpretation of data, acknowledging the diverse backgrounds and prior assumptions of the team.

## Results

### Baseline characteristics

Ten focus group sessions were conducted between May to September 2017 (see Table 1).

**Table 1:** Focus group participant numbers and demographics.

	Sessions	Number of participants	Female (%)
General Practitioners	2	7	4 (57.1)
ED Consultants and Trainees	2	24	10 (41.7)
Physicians	1	6	4 (66.7)
Basic Physician Trainees	1	7	5 (71.4)
Final-Year Medical Students	3	27	10 (37)
Second-Year Medical Students	1	15	3 (20)
**TOTAL**	**10**	**86**	**36 (41.9)**

**Fig 1:**
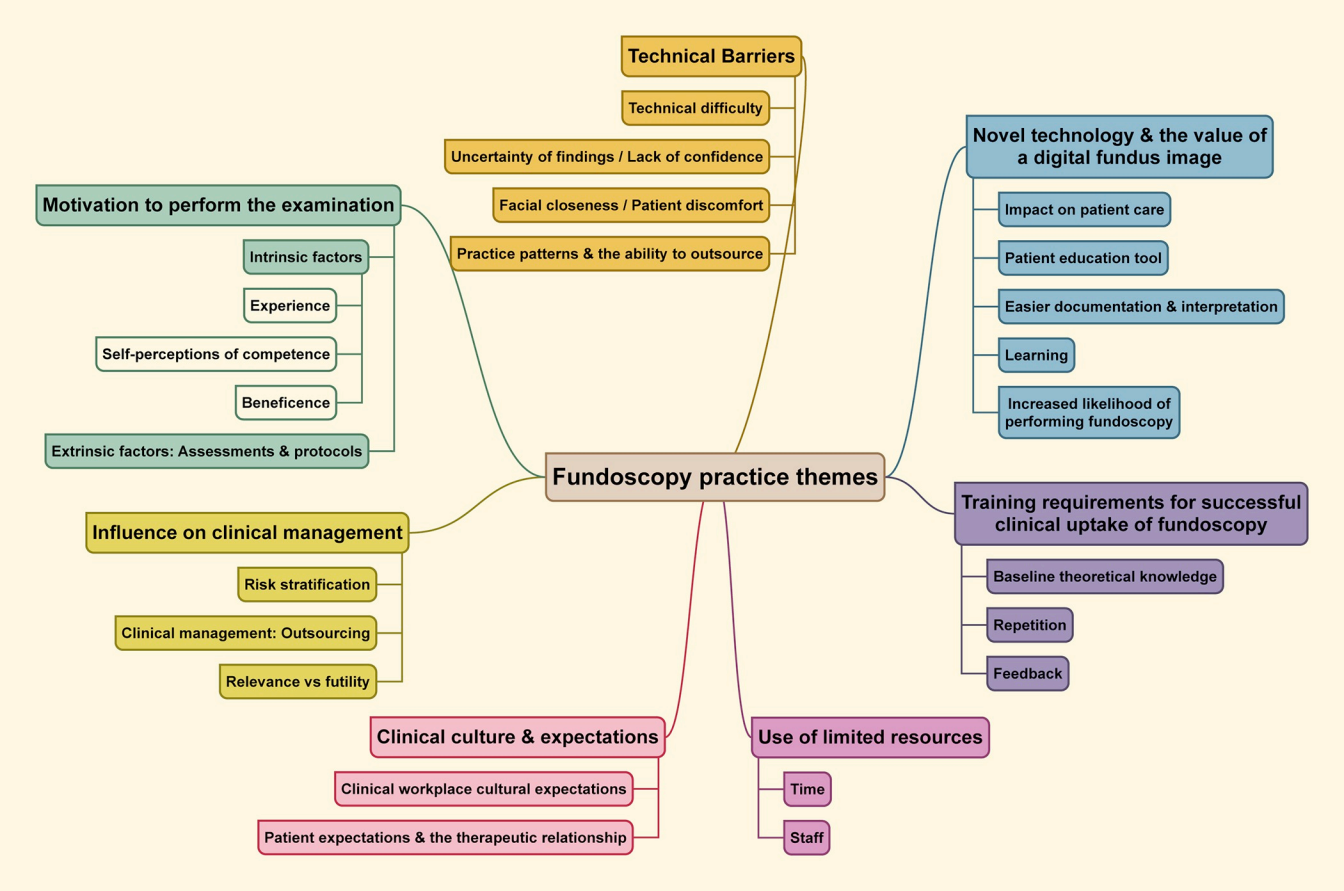
Themes arising from the focus groups regarding the barriers and facilitators of performing fundoscopy.

The seven major themes which emerged (Fig 1) are described below with representative quotes provided in italics (additional quotes referenced in brackets are found in S2 Appendix).

#### 1. Technical barriers to performing fundoscopy examinations

Participants voiced several key, interconnected barriers to performing fundoscopy examinations.

*Uncertainty of findings / lack of confidence*. Limited confidence to visualise the fundus was common to all groups, hence interpreting the findings was unrealistic for most (1.5, 1.6). Even when a fundal view was obtained, participants expressed poor confidence interpreting anything beyond florid pathology. Consequently fundoscopy has become perceived as futile.

“…*not knowing what I’m looking at*. *Particularly for papilledema*, *I guess if it’s obvious*, *it’s obvious*, *but the subtle changes*. *We see patients*, *they usually come here very early for stuff*, *they don’t come here with florid pathology usually*. *So picking up early changes and subtle things would be hard*.*”* (GP)

*Technical difficulties*. The technical difficulty of using TDO was identified as a barrier by all groups (1.1). Logistical barriers included: refractive difficulties with glasses; limited availability of reliable equipment (1.3); bright department lighting constricting pupils, and; the challenge of moving unwell ED patients to a dark space (1.2).

The patient cooperation required during an already technical fundus examination was challenging, especially amongst children. Medical students worried the bright ophthalmoscope light would cause discomfort to patients, hence would avoid practicing the technique.

*Closeness*. The face-to-face nature of fundoscopy was somewhat confronting for all, and a significant contributor was the perception of being observed by the patient whilst feeling incompetent at the examination (1.7). By contrast, doctors felt less concerned by physical proximity than medical students. One GP noted, “*There’s a lot of things that we do that we gotta get very close”*. Consequently, clinicians become, “*quite used to putting up a clinical shield*. *So you’ve got your doctor brain on*, *but actually when you’re doing it you don’t actually notice”* (GP).

*Practice patterns / ability to outsource*. For GPs, particularly in more affluent areas, accessibility to specialists and optometrists meant they were more likely to refer patients when fundoscopy was indicated, than to perform the examination themselves.

Frequency of ophthalmoscope use appeared to be modulated by confidence performing and interpreting the examination, and perceived clinical utility or futility in changing patient management. An ED Advanced Trainee expressed the problem thus, “*It’s inactivity*. *We don’t do it*, *we don’t remember how to do it*, *and then when we’re forced to do it*, *we’re not sure that we clinically interpret what we get*. *And there’s no embarrassment or weirdness about being face to face*, *it’s just I haven’t done it in so long*.” As barriers limit practice by clinicians and medical students, expertise is lost, reinforcing a cycle of reducing fundoscopy utilisation (1.8).

#### 2. Clinical culture and expectations regarding fundoscopy

*Clinical cultural expectations*. Medical Students perceived a culture of incompetence in fundoscopy, with no expectation that it would be performed for patients (2.1). Trainees described greater expectations to perform most other examinations. A Second-Year Medical Student stated, “*almost any doctor on the ward is comfortable taking blood pressures and showing us how to do it*, *whereas I think there’s perhaps even like*, *institutional avoidance of doing it* [fundoscopy].” Students perceived minimal expectations about their level of ophthalmic knowledge (2.2), and consequently, “*the culture of fundoscopy doesn’t really encourage us to be good at it*” (Final-year medical student).

Supervisor discouragement, while not intended by senior doctors, was universally experienced by medical students and training doctors. Perceived senior clinician disapproval of fundoscopic examination was described in acts of both omission and commission. Fundoscopy was visibly omitted from patient examination, and trainees absorb cultural expectations implicit in the behavior of senior clinicians.

“*I don’t think I’ve ever seen even a senior doctor do fundoscopy on any patient*. *And I guess you kind of model what you see in the hospital*.*”* (Final-year medical student)

Whilst there was no conscious disapproval of fundoscopy by senior trainees and physicians, they admitted their dismissive responses could build an unconscious culture of disapproval.

“*If they* [a junior doctor] *said*, *you know*, *does this person need a fundoscopy*, *I’d be like*, *yeah*, *sure*. *That’d be what the textbooks would say*, *but I wouldn’t know how to interpret it*. *Which would probably be read as discouragement*.*”* (ED advanced trainee)

ED doctors described a cultural expectation to attempt fundoscopy prior to requesting an ophthalmology consult, but without any assumption of competence (2.3, 2.7). At the extreme of this, one student observed a training doctor falsifying findings to meet the clinical assumptions of their supervisors (2.4).

Medical students and ED trainees described a period of conscious incompetence in training for any clinical skill when an examination such as ultrasound or fundoscopy may be practised but not documented (2.5). However for many, fundoscopy remains at this stage as a ‘dirty secret’ of clinical examination, to be occasionally performed but not reported.

*ED Junior Trainee 1: you still tend to sort of float around and do some. But you don’t really record your findings because you can’t make a clinical decision based on it, obviously unless there’s something huge and then you sort of, it gives you a clue. I don’t feel confident doing certain things, but I’ll still just put it on, have a look around, and see what I see. But I won’t document down that these are the definite findings. And I think that’s why, even if you try to do fundoscopy, I wouldn’t call it either way. I might do it secretly and not tell anyone, I might have a look, ’oh’…*

*Group: [laughter]*

*ED Advanced Trainee 2: I’ve had a peek behind the curtains*.

*ED Junior Trainee 3: I’ve had a few looks before and then just not told anyone that I’ve had a look because I don’t know what I’m seeing, really*.

Increasing medical specialisation was noted as a driver against fundoscopy use by non-experts. Fundoscopy has been relegated to the domain of ophthalmology and to an extent neurology. Second-Year Medical Students observed senior clinicians stating, "*Oh*, *no*, *no*, *we don’t do that*. *We just send them to the optometrist… or the ophthalmologist*.” Even physicians noted that endocrinologists referred their diabetic patients to the ophthalmologist rather than screening the fundus themselves.

*Patient expectations and therapeutic relationship*. General practitioners in a more affluent area noted patient expectations to see a specialist or obtain medical imaging reduced the potential demand for fundoscopy in patient management (2.8, 2.9).

#### 3. The influence of fundoscopy on clinical management

*Risk stratification*. Currently fundoscopy findings were seen to be more specific than sensitive, “*It*’*s more of a rule in than a rule out”* (ED Advanced Trainee).

However, ED, physicians and students felt fundus imaging would help screen for serious pathology to determine the patient’s disposition and thereby triage appropriate clinical review, “*hopefully it will reduce that hand-wringing”* (ED Junior Trainee) (3.1, 3.2).

*Clinical management*: *Outsourcing*. ED participants preferred to refer patients to specialists or utilise tele-ophthalmology, unless they became more competent at fundus interpretation themselves. There was disagreement about the level of experience and training required to confidently and safely triage presentations based on findings. Participants described experiential journeys through various stages of confidence and competence in examination (3.2, 5.9).

*Relevance / futility*. Most clinicians thought their fundoscopy skills were too unreliable to safely underpin patient management (3.3).

“*Unless it becomes very evident to me that what I’m seeing in fundoscopy is abnormal*, *I would not base my further management on that*, *what I’m seeing*, *because I don’t trust it*.*”* (ED Advanced Trainee)

Whilst some in community practice felt fundoscopy would not change their management plans (3.4). These barriers have engendered an overall perception that fundoscopy is futile, which exacerbates avoidance of fundoscopy (2.7–2.9).

#### 4. Motivation to perform the examination

Motivational factors of particular importance to subgroups are summarised in Table 2.

**Table 2:** Factors affecting motivation to perform clinical examinations such as fundoscopy: Common to all groups, and; particularly noted amongst different clinical groups.

Participant group	Intrinsic motivating factors	Extrinsic motivating factors
Common to all groups	Competence	Barrier assessments
	Beneficence	Protocols
	In/experience	College requirements
SUBGROUPS		
Medical Students		Barrier examinations
ED Trainees	Risk stratification	Protocols
	Harm minimization	Accredited courses
	Avoiding missed pathology	
Physicians		Accredited courses
		Curriculum vitae building
General Practitioners	Screening	
	Catching rare conditions	
	Academic interest	

*Intrinsic motivating factors*. Self-perceptions of clinical competence were important to all participant groups (4.1, 4.2). Conversely, most doctors felt inexperienced and incompetent with fundoscopy, hence were reluctant to employ it.

“*Or to ask a patient to like sit through me being incompetent for that amount of time… I honestly think I’d be more comfortable*, *like if you were to right now give me the option of go do a speculum exam and a pap smear*, *or do direct ophthalmoscopy*, *I’d probably take the pap smear*.*”* (Female, Second-Year Medical Student)

Beneficence was elucidated as a strong motivation behind choosing examinations (4.6), as expressed by one second-year medical student, “*We come into medical school so that we can help people and so that we can hopefully make a difference*, *improve their quality of life*.*”* Whilst clinical curiosity was a variable, weaker motivator (4.4). When asked their motivations to perform specific examinations, ED generally agreed, “*It*’*s risk stratification*.” (ED Junior Trainee) (4.5, 4.6).

Accordingly, ED doctors were more motivated to detect pathology which would change their emergent management, rather than subtleties of chronic eye conditions more relevant to specialists. “*I’m trying not to miss the glaringly obvious*, *not that I’m trying to pick the really subtle*.” (ED Junior Trainee)

For GPs, motivation to perform an examination was more individual. Some liked the capacity of screening to detect the occasional highly significant disease such as finding melanoma amongst many normal skin checks. Others were most proud of rare diagnoses, such as Addison’s disease, detected early through more in depth examination. Underpinning both these categories of motivation was the sentiment that, “*You feel like you’ve actually saved*, *like altered the course of their lives*.” (GP)

*Extrinsic motivating factors*: *Assessments and protocols*. Barrier assessments are the key time to ensure a skill is appropriately learnt, as the expected competency level dictates the effort applied by students and trainees (4.7).

Several checkpoints in the medical training pathway were identified as potential intervention times to motivate better fundoscopy capabilities: 1) medical student examination requirements likely set future capabilities (4.7); 2) certified accreditation courses targeted prior to specialist training admission could enhance trainees’ curriculum vitaes; 3) specialty college examinations motivate trainees to learn whatever is required to pass (4.8), and; 4) career progression hurdles may be a useful forcing function (4.9).

Clear guidelines on the relevance of fundoscopy to patient management would help motivate fundoscopy training, “*Enough for me to want to commit to doing a learning programme on it when there are competing priorities*” (GP). Physicians suggested using standardised protocols for ED presentations like headache may improve fundoscopy rates. However the diversity of aetiologies and presentations requiring fundoscopy would pose a greater challenge than established protocols like chest pain.

#### 5. Novel technology including smartphone fundoscopy, and the value of a digital fundus image

*Impact on patient care*. Participants noted a fundus photograph would give doctors an objective measure of clinical findings, improve confidence in fundoscopic findings and help direct clinical management. A digital image can be sent to other clinicians for advice, which could reduce the barriers to specialty consultation and streamline intervention as it currently does in other specialities such as surgery, dermatology or orthopaedics (5.1).

Fundus photographs would help workflow, on busy ward rounds when a patient may wait all day for a consultation, and especially in ED. Patient triage and discharge may become safer (5.2). ED trainees noted a concurrent educational and cultural shift would be needed as they became more confident interpreting the images themselves.

“*We’ll probably still be speaking to opthalmologists about the same cases*, *(but) we’ll have a bit more information for them and I think it will change the disposition*, *and it might mean they get seen in a few days or they get seen straight away*.*”* (ED Junior Trainee)

Integrating tele-ophthalmology feedback would likely incentivise more frequent fundus examination (5.3).

*A patient education tool*. A fundus photograph could help patients visualise their eye health and assist GPs to motivate lifestyle changes (5.4).

*Documentation and easier interpretation*. The practicality of a documented clinical image to be interpreted and reviewed at leisure with colleagues would reduce the uncertainty and discomfort surrounding TDO (5.5–5.7).

*Learning*. Whilst fundoscopy is currently performed rarely, it may take some time for increasing use to feed back into optimised patient management. Repeated pattern recognition seeing fundi in clinical context would help emergency physicians decide which patients need further investigation or could be safely discharged. Whilst ED may initially over-refer or over-investigate patients (5.8), outsourcing of clinical decision-making should reduce with experience and training.

Introducing novel examination technologies could be difficult for senior doctors (5.9) with some participants anticipating initial resistance (5.10). Additionally, senior physicians expressed concern that fundus interpretation would remain challenging (5.11), or that the ease of consultation using a digital photograph could provide a perverse incentive against interpreting fundoscopic findings (5.12).

*Likelihood of performing fundoscopy*. It was noted that a fundus image would reduce the number of examinations required of a patient, and would allow doctors to, “*get a consensus from consultants*” (ED Junior Trainee). A fundus image also mitigates the responsibility on the examiner to diagnose pathology which is a deterrent to performing TDO.

“*I’d be more likely to do it*, *knowing that like*, *you know*, *it doesn’t rest with my interpretation*, *because like that’s the thing that’s been difficult with fundoscopy is that if you look*, *it rests on your interpretation*.*”* (Final-Year Medical Student)

If novel fundoscopic technology such as smartphone adaptors or fundus cameras were integrated into medical school, many students felt the technical barriers to learning and practicing fundoscopy would be minimised: “*we’re in this kind of almost transition period between what has been tradition and what might be the possibility coming*.” (Final-Year Medical Student, 5.13)

#### 6. Training requirements for successful clinical uptake of fundoscopy

*Theoretical knowledge*. Participants voiced difficulties with the technical use of TDO and interpretation of their findings (6.1). All groups raised the prospect of baseline fundoscopic competencies and tailoring the extent of knowledge required within different specialties and training levels. There were mixed opinions about the appropriate training level required to interpret fundoscopy. ED trainees described learning pathways in other examinations, from unconscious to conscious incompetence, and finally expertise (3.2, 5.8).

A cultural shift towards a basic recognition of abnormal fundoscopy could be a reasonable educational target (6.2, 6.3). Medical students and ED groups expressed a need to improve understanding of the implications of fundoscopy on patient management and outcomes.

“*I guess being told stories about how a simple fundoscopy can prevent so many complications to a patient and I think as medical students*, *we aren’t taught the value of sight and how easily it can be lost*.” (Final-Year Medical Student)

*Repetition*. All participant groups agreed the lack of repetition of fundoscopy practice resulted in significant attrition of skills. With comparable skills such as otoscopy, looking at multiple normal patients allowed an experience and recognition of the range of normal (6.4). By comparison, the lack of repetition of fundoscopy skills means clinician expertise languishes (6.5),

“*You get stuck in this cycle of not dilating people because you don’t feel confident to look at the back of their eye*, *and then you’re like*, “*well I’m not just going to dilate them pointlessly*,*” and then because you don’t dilate people*, *you don’t look at many eyes*.*”* (ED Advanced Trainee)

Repetition of ophthalmoscopy to understand the range of normal is logistically difficult in ED, however less technically demanding fundoscopy would facilitate repetition of skills (6.6).

If fundoscopy findings were reinforced in regular teaching meetings, as occurs for radiology, trainees would more likely recognise relevant abnormalities (6.7).

*Feedback*. Feedback on technique and interpretation of findings reinforces clinical skill development. Large group training was felt to be insufficient for ophthalmoscopy, as there was little capacity for feedback and individual practice. Many trainees reflected that they did not feel competent in fundoscopy at the end of medical school. Objective ophthalmoscopic competence was not assessed by medical schools, and some participants felt pressured to feign competence to avoid embarrassment (6.8).

A fundus image was preferred for ED training (6.9).

Participants noted comparable training and accreditation pathways, like ultrasound, which could model improved fundoscopy. Notable steps in this process were a readily achievable fundus photograph, easy access to repeated feedback on interpretations, and re-accreditation methods like online training (6.10).

#### 7. Use of limited resources

*Time*. Time-efficient and practical learning and utilisation tools will be necessary for uptake of fundoscopy. The importance of this is absorbed early (8.1). The technical demands of fundoscopy, alongside a training structure that does not reward competence, leads trainees to abandon fundoscopy.

“…*the times when I’ve tried it and I’ve just blatantly failed*. *And you know*, *it’s that moment where; do I keep wasting time just pursuing something that I’m not really going to know if I’m seeing anything or any pathology*, *I don’t even know if I’d recognise it; or do I just spend more time on the things that I’m more confident I’ll get*.*”* (Final-Year Medical Student)

The practical requirements of TDO including a darkened room, dilation drops and the time required for them to work, limited uptake amongst ED and GPs.

*Staff*. Duplication of service currently occurs whenever a junior doctor performs fundoscopy, as the senior clinician, neurologist or ophthalmologist will have to re-examine the patient. Whereas a fundus photograph would allow convenient review.

## Discussion

We explored perceptions and experiences of undertaking fundoscopy amongst medical students, doctors in training and consultant doctors across general practice, ED and medical specialties.

Multiple technical barriers limit TDO performance. Challenges common in the literature were expressed in our study including patient cooperation, ambient lighting causing pupil constriction, the impracticality of moving unwell patients to darkened areas, difficulty obtaining a fundal view, concerns about pupil dilation (which is helpful although not strictly necessary) and device failures [4].

The interpersonal proximity during TDO examination was problematic for medical students, but less so for doctors who described having a clinical ‘shield’ to cope with recurrent clinical breaches of usual interpersonal distance. Whilst physical proximity is required in many physical examinations, the face-to face nature of TDO seems to generate qualitatively different barriers. The awareness of being face-to-face during a prolonged, novice examination underscored student discomfort and reduced their motivation to practice fundoscopy. Furthermore, our study was completed before the onset of COVID-19 which amplifies concerns of facial proximity and consequent risks of disease transmission [17]. All participant groups identified perceptions of professional competence as a motivation, and performing fundoscopy (in which they feel minimally competent) is worse for the awareness that the patient is watching. As students and doctors wish to avoid causing discomfort to the patient, shining a light into the patient’s eye was concerning. These factors could modulate the positive effect of practice on confidence with fundoscopy found in other studies [18, 19]. Overcoming these intermediate steps may facilitate greater repetitive practice, which our participants report underlies competence in more frequently performed examinations.

Several studies have explored confidence in fundoscopy [18–20], based on the assumption that those less confident are less likely to perform the examination. Dalay et al. found that doctors’ perceptions of their own TDO competence were directly related to their seniority [4]. Akaishi et al. found that experience using the TDO was a predictor of proficiency using a fundoscopy simulator [21]. Experience using TDO has been correlated with greater confidence to perform fundoscopy, however confidence to recognise major pathologies was only associated with experience seeing eyes with pathology, not just normal eyes [18]. Indeed confidence may correlate poorly with clinical utility or proficiency [22]. One study found medical students who were less accurate at identifying a fundus using TDO, were actually more confident in their answers than those more proficient [23]. Participants in our study described an educational journey of increasing confidence with using examinations or investigations like fundoscopy, ECG or ultrasonography in their clinical management pathways. Our results suggest that a biphasic pattern may be common as trainees describe early incompetence and associated poor confidence; then further experience can initially lead to overconfidence with an ignorance of their limitations. When later studying for specialist exams, our participants proposed that doctors often become less confident as they learn of the complexities of an examination before finally achieving competence and clinically appropriate confidence, balanced with an awareness of their limitations. These findings align with the Dunning-Kruger model of self-perceptions of competence [22, 24], and it seems reduced confidence using fundoscopy at any of these stages may result in trainees giving up on fundoscopy. Our results suggest there may be a threshold of competence with the technical examination procedure and interpretation of fundoscopy that generates sufficient confidence to drive repetitive practice and increasing mastery over time.

To achieve such a threshold of technical examination competence, several strategies are proposed in the literature. A randomised trial comparing TDO simulation with traditional learning found simulation use improved duration of practice and technique scores, with possible gains in lesion localision, but no significant change to global scoring [25]. Standardised TDO performance criteria have been validated [26]. Randomised trials amongst medical students have demonstrated that flexible e-learning video instruction led to greater diagnostic accuracy than face to face instruction (63.4% vs 36.6%, p < 0.001) in Japan [27], and combining video and live demonstration had a synergistic effect on TDO technique in Sri Lanka [28]. A systematic review of non-simulator methods for TDO instruction found volunteer fundus photograph matching, ocular pathology instruction, small-group training and video improved learning outcomes [29].

Novel fundoscopy technologies such as smartphone fundoscopy and non-mydriatic cameras have the capacity to obviate many of the technical barriers of TDO [8]. Direct fundoscopy takes years to perform competently [30], whilst most studies have found smartphone fundoscopy is preferred by medical students and junior doctors [9, 31] and has greater accuracy [14, 32, 33]. Such devices offer the advantage of a shared view for feedback during training, which was important to optometry students [34], and has been shown to improve clinical performance [35]. This suggests the emergence of a significant paradigm shift that could, in many contexts, improve the appropriate use of fundoscopy, without the need for TDO.

Our ED participants in particular felt that an easily obtainable digital fundus image would: provide an objective record of the findings; facilitate interpretation without the temporal and technical pressures of TDO; enhance telemedicine support; streamline specialty referrals, and; translate to more efficient patient care. Using TDO places all responsibility for a challenging examination with a single doctor who usually feels inexperienced and incompetent [18–20]. Concerningly, we found that TDO will be avoided, findings may not be documented, or may even be falsified as a consequence. Such patterns of under-, over- and inappropriate documentation were identified amongst 96% of videotaped physical examinations in a study of medical students [36]. Clinical cultures and documentation pathways can, in some cases, induce incorrect or even fraudulent physical examination documentation [37]. By contrast, a digital image mitigates this barrier of personal examiner responsibility by facilitating easy, rapid, collegiate review [8]. Indeed a prospective study of portable, non-mydriatic fundus photography with telemedicine ophthalmic support, performed after routine TDO in a rural ED, found subsequent management was altered for 39% of 133 patients [38]. Moreover, even before fundus interpretation training or telemedicine support, ED doctors’ sensitivity to detect acute life-threatening or blinding pathology increased from 0% using TDO to 29% using a non-mydriatic fundus photograph, and their sensitivity improved from 59% to 84%.

Across the medical spectrum, we identified pervasive clinical cultures which relegate fundoscopy to ophthalmology, optometry or neurology. Participants across different hospital sites in our study suggested that perfunctory fundoscopy performance may be required for an ED referral, yet there was no expectation of competence. Senior clinicians, beleaguered by relatively infrequent fundoscopy practice and the perennial challenge of interpreting findings, usually omit fundoscopy or express their dismissive perceptions of the futility of the exam, but did not consciously discourage fundoscopy. These expressed and implied cultural norms are absorbed by medical students and trainees, reinforcing a cycle of underperformance of fundoscopy. Our finding suggests the perception of disapproval by seniors which was reported as a barrier to fundoscopy by 20% of medical students in the ToTEMS 2 study [11], and identified in other studies [4], may in fact be a *mis*perception of disapproval. Our results suggest that interventions to increase fundoscopy use should address cultural factors across the hierarchy of medical training.

Our participants reported fundoscopy interpretation is challenging for all but florid pathology. Yet GPs more often address early presentations, and ED doctors need to manage risks amongst patients where the clinical signs may be subtle. These doctors’ concerns about their sensitivity interpreting the fundus limit their sense of safety to integrate fundus findings in patient management. Hence they come to perceive fundoscopic examination as futile. Training to interpret fundoscopic findings is essential to overcome these barriers, and interpretation checklists could help. Ophthalmic overview of digital fundus images may help support other doctors as they upskill, but some participants were concerned this could provide a perverse incentive against learning. Telemedicine review of fundus images in the ED has been shown effective in the USA [30] and Australia [38, 39]. As the clinical applicability of artificial intelligence improves for conditions such as papilloedema [40], computerised diagnostic support could supplement ophthalmic oversight. Our participants felt they would progressively become more competent in identifying pathology, especially if they received continual feedback on their diagnoses. This strategy has proven effective in diabetic retinopathy and glaucoma screening [41, 42].

The place of fundoscopy in clinical management guidelines warrants reinforcement, and medical education should clarify the practical implications of fundus findings to increase the salience of the examination and overcome the historical barriers to implementing fundoscopy in clinical practice patterns. Focusing on the benefit of fundoscopy for patients would engage the motivation of beneficence [43], which we found much stronger and ubiquitous compared with academic curiosity about the fundus. A study of 102 patients found simply providing GPs with an audit of their hypertension management practices benchmarked against clinical guidelines improved the rate of fundoscopy from 11% to 54% [44]. Similarly, after a case of avoidable blindness from missed papilloedema, two UK teaching hospitals found employing a patient audit tool and personalised feedback to doctors likely had a greater effect on fundoscopy utilisation than audit, training and education [45].

An interactive practical ophthalmic skills education session for 121 GPs resulted in increased self-reported fundoscopy performance at both six and twelve months [46]. However, after the introduction of fundus photographs, an e-learning interpretation course failed to further improve diagnostic accuracy of ED physicians [47]. Spaced education has been shown to improve clinical skills [48], yet a three-year curriculum retraining medical students in ophthalmology examination improved examination performance but failed to increase the frequency of fundoscopy [10]. Our participants suggested fundoscopy uptake would be improved by individual feedback, repetitive practice of examination and interpretation skills, and accreditation of training. Further research to combine these factors is warranted.

Our study provides a more complete picture of the complex balance of barriers and facilitators which interact to drive fundoscopy practice across the spectrum of medical practice.

### Strengths

The trustworthiness of our analysis was optimised by incorporating: a facilitator who was not an ophthalmologist; an inductive grounded theory approach to qualitative data analysis; independent thematic coding analysis by three authors to develop a consensus framework, and; repeat analysis by two authors. The credibility of our results was improved by triangulation of sources, with a large number of participants from multiple tiers of medical training, in different hospitals and training networks, and repeated sampling until thematic saturation.

### Limitations

Senior doctor participants were enrolled by snowball sampling which may be prone to selection bias and issues of representativeness. However our results amongst this group are strengthened by a breadth of medical specialties and the fact that themes from this group concurred with other groups. Amongst trainees, we attempted to minimise bias towards participants interested in ophthalmology by running focus groups during protected break times and in convenient locations.

Medical students and ED trainees participated in the focus groups after practical training using traditional ophthalmoscopy and two forms of smartphone fundoscopy. A recency bias could therefore impact their discussion of novel technologies.

Medical cultures can be context-specific. Although we sampled participants whose patient catchments have differing socio-economic and ethnic backgrounds, our results would need replication in dissimilar health systems to confirm generalisability.

## Conclusion

The frequency of ophthalmoscopy use appears to be modulated by confidence performing and interpreting the examination, and perceived clinical utility or futility in changing patient management. As barriers limit practice by clinicians and medical students, expertise is lost, reinforcing a cycle of reducing fundoscopy utilisation. We identified important cultural barriers such as a culture of accepted incompetence, and misperceptions of senior discouragement. Emerging technologies reduce the technical barriers to fundoscopy, and could herald a paradigm shift to optimise fundoscopy uptake, without the need for traditional direct ophthalmoscopy. Therefore education should: focus on detecting pathology from digital images; clarify the role of fundoscopy in optimal patient management, and; be targeted at key career progression points. Our results identify a more nuanced picture of the interacting factors which determine fundoscopy utilisation, and which need to be addressed in order to revive this valuable clinical examination.

## Supporting information

S1 Appendix Focus group prompt guide. (DOCX)

S2 Appendix Additional quotations. (DOCX)

S1 Dataset De-identified focus group transcripts—minimal dataset. (ZIP)

## List of abbreviations

ED: Emergency department
GP: General practitioner
TDO: traditional direct ophthalmoscopy
