# Supplementary material for: Optimising fundoscopy practices across the medical spectrum: A focus group study
Source: PLoS One. 2023 Jan 27;18(1):e0280937. doi: 10.1371/journal.pone.0280937 (PMC9882965; doi:10.1371/journal.pone.0280937)
Supplement: S1 Appendix — (DOCX) [file pone.0280937.s001.docx]

## S1 Appendix. Focus group prompt guide

Introduction:

- We're here today to ask how you use fundoscopy and other clinical skills.
- What are the pragmatics of performing fundoscopy?
- How does fundoscopy fit into your management practice patterns and what the barriers are to using it?
- What things would motivate you to use fundoscopy as a clinical skill going forward?
- Just a reminder that all information from today’s discussions will be de-identified. Nothing will be reported back to your hospital or training network, and you are free to leave or ask to stop recording at any point.

Case example to commence/facilitate discussion:

- 6-year old with five sequential GP & ED presentations for headache then progressive visual loss
- diagnosed psychiatric conversion syndrome and eventually referred to ophthalmology
- actually had gross papilloedema with raised intracranial pressure, but no fundoscopy had been performed during any previous clinical examination
- final outcome was legal blindness in both eyes

Prompts:

- How often would you perform fundoscopy in routine general (or neurological) examinations?
- How do you use fundoscopy in your patient workflow/practice patterns?
- What are the barriers to performing fundoscopy?
- What are the motivators to improve your clinical skills mastery?
- What is your rationale for doing exams? Is there an underlying impetus for you in medicine, like picking up a rare condition that was otherwise missed, or screening consistently to catch a disease early?
- Do you think that smartphone or digital fundoscopy (we tried today) would change your use of fundoscopy? How? Why?
- The literature describes active discouragement of fundoscopy by senior medical staff. Do you ever notice any positive or negative senior clinician responses to fundoscopy?
