# Supplementary material for: Optimising fundoscopy practices across the medical spectrum: A focus group study
Source: PLoS One. 2023 Jan 27;18(1):e0280937. doi: 10.1371/journal.pone.0280937 (PMC9882965; doi:10.1371/journal.pone.0280937)
Supplement: S2 Appendix — (DOCX) [file pone.0280937.s002.docx]

| **Technical barriers to performing fundoscopy examinations** | | |
| --- | --- | --- |
| Quote Number | Participant details | Quote |
| Technical difficulties | | |
| 1.1 | *Female ED Trainee* | *“when I wore glasses, I found it extremely technically difficult to try and do a fundoscopy... So it was just impossible to use. And even now that I've been wearing contacts for a few years, I still don't do it because it's just too damn hard to do in ED.”* |
| 1.2 | *ED Registrar* | *“ it depends where you see them. Because if you see someone in the main arena it's bright, and you haven't got that high level of suspicion. You're probably not gonna move them over to the eye room and dilate them.”* |
| 1.3 | *Male ED advanced trainee* | *“Fundoscopy, not with our things that hang on the back of the walls, and those don't work. No.”* |
| Uncertainty of findings / Lack of confidence | | |
| 1.5 | *Medical student* | *“I think the biggest barrier for us is we're not confident in skills and we don't know how to interpret the things yet. What you see. So yes, if you made it easier to access the retina and visualise that, it's going to be easy to interpret because you can see it quicker and easier.”* |
| 1.6 | *Female ED advanced trainee* | *“I think it's very limited, it's very hard to get really good to use? Even if I do manage to get partial view, I cannot see like at the same time, macula and disc, and just to describe full, whole picture of the eye. You may see normal vessels, or some haemorrhage if you like in that view, but you cannot actually get (a) very good full picture that you could describe well.”* |
| Closeness | | |
| 1.7 | *Final Year female medical student* | “*Or to ask a patient to like sit through me being incompetent for that amount of time… I honestly think I'd be more comfortable, like if you were to right now give me the option of go do a speculum exam and a pap smear, or do direct ophthalmoscopy, I'd probably take the pap smear.”* |
| Practice patterns | | |
| 1.8 | *Female GP* | *“I do do it sometimes, but very rarely, and there's loads of reasons why not. One of them is time, and another is lack of knowledge, lack of expertise, I've lost that expertise.”* |

| **Clinical culture and expectations regarding fundoscopy** | | |
| --- | --- | --- |
| Quote Number | Participant details | Quote |
| Clinical cultural expectations | | |
| 2.1 | Medical student | *I guess we haven't been taught that fundoscopy is integral in examination of the patient*. |
| 2.2 | Medical student | *the undercurrent is unless you're going to specialise in that area, you don't really need to know it*. |
| 2.3 | Male ED Registrar | “*I've done it knowing I was going to speak to Eye Reg and I've always said, I've attempted this, I've no confidence to say one way or another what I'm seeing*.” |
| 2.4 | Medical student | *I had neurosurg say once he sucked at fundoscopy and so when he thinks that clinically the ICP is raised he just says to his bosses, "Oh yeah, papilledema," like he really can't see.* |
| 2.5 | Medical student | *I'd do it if it was clinically really important, but then I wouldn't document that it's normal, because I wouldn't be confident that I knew it was normal.* |
| Futility | | |
| 2.6 | *Male ED Advanced trainee* | *I don't think I discourage them from doing it, but it's a test that I'm gonna take absolutely nothing from, because I think in my head I assume that if I can't do it… and I assume that people above us can't do it very well, I'm not going to take the intern's* [assessment of]*, what he sees, unless he used to be an optometrist or something.* |
| 2.7 | Female ED Trainee | *The patient's come in with a headache and things like that, ... and they say have you looked at the eyes? And I've said look, honestly? It's so technically hard to do, I doubt I could see what I need to see, and even if I do see it, it's gonna be absolutely tiny, I'm not gonna be able to tell squat about it. So no, sorry, I haven't done it.* |
| Patient expectations and therapeutic relationship | | |
| 2.8 | Female GP | *So if you have somebody who you're suspecting say … brain cancer or something like that, is having headaches and vomiting, you're thinking regardless of what you see on fundoscopy that patient is being sent off for an MRI or a CT scan. They're being sent urgently to a neurologist, and part of that is I think a privilege to work where we work.* |
| 2.9 | Female GP | *Like if you had* [a] *really good relationship with the patient, I think they really do trust our opinion. But there are times when I just go, "Okay, off you go”, because I know that if you hear it from a specialist you'll do it.* |

| **The influence of fundoscopy on clinical management** | | |
| --- | --- | --- |
| Quote Number | Participant details | Quote |
| Risk stratification | | |
| 3.1 | ED advanced trainee | *Sometimes you'll have six or eight people who just pile up, one after the other, with very non-specific symptoms. A bit of headache, or tingling here, tingling there, just normal examination. So I think those ones actually need to have the fundoscopy and then probably we'll have a bit more sort of reassurance that, okay, I'm going to send you home* |
| 3.2 | ED Trainees | Male Advanced Trainee*: Chest pain's obviously a very common presentation, as is headache. The experience base I think you require for chest pain to be getting to a point where a certain portion of them, you're confident to send home without discussing with cardiology, I think comes probably - you probably need to be an advanced ED trainee*  Female Trainee*: I'd say the opposite, I think that when you start studying for your exams, you then send less patients home, cause you find out all these things…* |
| Relevance | | |
| 3.3 | ED Advanced trainee | *the consequences of calling a false negative are so potentially catastrophic as well.* |
| 3.4 | GP | *I can't really think of any situation in which it might change my management.* |

# S2 Appendix. Additional quotations.

| **Motivation to perform the examination** | | |
| --- | --- | --- |
| Quote Number | Participant details | Quote |
| Intrinsic motivating factors | | |
| 4.1 | Male GP | *I think all doctors want to feel more competent*. |
| 4.2 | Female medical student | *If I came out of my degree knowing that if I went into the ED and someone had a dodgy eye, I would be able to recognise it, for me, that would be enough. I want to be clinically competent. I want to be a decent doctor when I head into an ED. For me, that's probably the most important part*. |
| 4.3 | Male ED trainee | *I think it's just, we don't see enough, and I guess it's the decision to actually do fundoscopy in the first place. Because we don't have that sort of knowledge-base you're getting through that experience with it.* |
| 4.4 | Female GP | *I might have a look out of interest sake. I probably wouldn't see anything but I would always send that patient to a specialist as an emergency anyway, regardless of what I saw.* |
| 4.5 | Female ED Trainee | *For me, I don't want to send someone home knowing that I could have done the exam and I could have caught something*. |
| 4.6 | Male ED Trainee | *And the risk management is pretty fundamental to what we do. I mean what you do is to preserve vision. What we do is to resuscitate, the obviously extreme, and to risk manage the 90% of the other presenters to our service. Anything that helps to reduce the risk, I think we're motivated to do, and we would like to be better at it.* |
| Extrinsic motivating factors: Assessments and protocols | | |
| 4.7 | Female medical student | *I think it should be more examinable. Only because that's why we have OSCEs and we know how to do a shoulder exam, because we had to learn it for an assessment and I feel that is a main motivator.* |
| 4.8 | Physician | *If you then get the colleges to say you have to have at least done this course once and tick it, they'll do it.* |
| 4.9 | Physician | *Unfortunately I think in the scope of medicine you need barriers to make people do a lot of things. Because usually it's always when you come out in the real world, that's when you go, "Oh, actually, crap, I need to learn how this happens.*" |

| **Novel technology including smartphone fundoscopy, and the value of a digital fundus image** | | |
| --- | --- | --- |
| Quote Number | Participant details | Quote |
| Impact on patient care | | |
| 5.1 | Medical Student | *Before the registrar comes down, you will take a picture and ask them, “Do you want us to deal with this or do you want to intervene?”* |
| 5.2 | Female ED Trainee | *You now have a hard copy that you can transmit to the Eye Reg and they can tell you if the patient's safe to go home today.* |
| 5.3 | Male medical student | *I think you'd also create the expectation you'd do it more if the ophthalmologist like, was expecting to get them more frequently* |
| A patient education tool | | |
| 5.4 | Female GP | *I reckon ... just say for the diabetic, hypertensive person who has been a bit borderline for a long time, it might ... Being able to take a photo, show them those changes or explain to them those changes, that might ... Because you can actually see, it's the only place you can really see the blood vessels. It might actually persuade them to be treated.* |
| Documentation and easier interpretation | | |
| 5.5 | Male ED advanced trainee | *...and the impression you have to rely on, whereas here you can take a photograph and you actually can look at it again and say, look, I think this is actually a papilledema...* |
| 5.6 |  | *Male ED advanced trainee: “having a fractional second maybe where you sort of see something, an impression -*  *Female ED trainee: And that's assuming your patient's compliant.*  *British male ED Trainee: And you can show the other docs like an ECG, I'm not quite sure about this, what do you think? Rather than, can you come look at this eye as well.”*  *I think it's the interpretation which is the hard ... PR, it's basically putting your finger in a hole and seeing, can he squeeze or is there anything coming out. Whereas a fundoscopy, you've got - the skill's harder to do, and then what you're seeing you then need to interpret in a difficult way. I think having the photo will be so much easier, cause then we can ... you've got time to sit down, look at it, think about it, without someone breathing on you face-to-face.” (Male ED Trainee)* |
| 5.7 | Medical student | *I do like the idea that I can have instead of just a glimpse over a second, that I can bring up a picture and then go through an algorithm that I can, or a series of steps, to analyse what I'm looking at, in the same way that when you give me an ECG, I'm just going to think, you know, it's one step at a time. Right?* |
| Learning | | |
| 5.8 | ED trainee | *I do think initially when we're seeing these images, for me, perhaps for others, they'll be less - obviously we will see some pathology, happy to refer, seeing things that we think are normal and saying I'm going to send this patient away without talking to you, I think that would require a little bit more confidence, and that's probably going to be a bit slow. I think that would come with teaching*. |
| 5.9 | GP | *I think it's just about teaching an old dog new tricks again. Like just changing practise*. |
| 5.10 | Physician | *We need to get the old physicians to learn this as well. And so maybe the college would have to also run some education sessions because most of them are resistant to new technology or a lot of them will have no idea what it is and then they'll say it's cheesy like they did with the electronic stethoscope and the panoptic ophthalmoscope. I think it's gonna take some time to get this in but we're gonna have to target really all levels to get it accepted.* |
| 5.11 | Physician | *I still worry about whether people can still need a bit more training to recognise the abnormalities.* |
| 5.12 | Physician | *As long as it doesn't become a misuse of technology. I suppose that the end result of this will be nobody will be actually trying to look at fundi. They'll be taking photographs and sending them off and you guys.* |
| Likelihood of performing | | |
| 5.13 | Female Medical Student | *I think I like the new technology because I think it eliminates concerns people have about getting too close to patients and I think it provides ... You can get feedback on the technique. It gives you a record that you can then take your time, rather than bothering a patient for 20 minutes looking. Take your time to write out something that you can then hand over. Gives you an image that you can send as part of asking for a consult. I think if we had that technology now, and there were opportunities within the medical programme to practise with it and we knew the basic sort of lecture style didactic teaching, like we knew what the conditions were, then I feel like a lot of the issues that we have would become null and void in some ways. It's just that we're in this kind of almost transition period between what has been tradition and what might be the possibility coming.* |

| **Training requirements for successful clinical uptake of fundoscopy** | | |
| --- | --- | --- |
| Quote Number | Participant details | Quote |
| Theoretical knowledge | | |
| 6.1 | Female medical student | *I think it's two separate issues really with funduscopy. There's step one, which is actually getting a decently focused image so that you can see something, and then step two is knowing what that image is and being able to interpret that image. You're not really trying to teach one skill, you're trying to teach two separate skills kind of mashed together which is the hard part.* |
| 6.2 | Male medical student | *Maybe if you want to step up our education, like for our generation. It should be what's normal and what's abnormal. If we can say, "This is abnormal, let's refer." I think that's already a big enough step.* |
| 6.3 | Male medical student | *Well, what I want to be really good at as a junior doctor in ED is the same as for ENT and everything else, is be able to do a proper funduscopy and have a list of things that I want to check for and say, "This is not normal," and be able to refer properly rather than be part of the culture that says, "Oh, it's part of the eye, so let's just give that to ophthalmology and not bother with that because no one else seems to bother." But if you bother with the ear, why not with the eye? They have a system there. It all comes back down to exposure and training in medical school. If you give students very early on in their medical degree a checklist of things that they have to do and look for and what an actual fundus looks like, and you just brush them with tonnes and tonnes of normal and abnormal images, by the time they reach internship, it'll be second nature for them. Even if they can't do things properly, they'll be able to say, "This is abnormal."* |
| Repetition | | |
| 6.4 | Medical student | *“Go to the ward and find 30 patients who are normal and then try and look for pathology." So, we just practised actually doing. I think that's the best way.* |
| 6.5 | Female ED Trainee | *You went onto the wards and never did it for six months or a year, and then you came and that's what you did.* |
| 6.6 | Male ED trainee | *If it takes 10 seconds, we haven't got any excuse not to do them, really. All our excuses are at the moment that we don't do it often enough, we could do this on every patient. And that we aren't confident, we can practise on every patient.* |
| 6.7 | Male ED trainee | *I guess once we've seen enough, we'll learn enough - basically exactly the same thing that we do with radiology. Cause we see so many chest x-rays.* |
| Feedback | | |
| 6.8 | Male ED Trainee | *here's this ophthalm, he's got this look in his eye, this is what you should see. And I'm sure everyone would just say yeah, I'm seeing that, because you didn't want to look an idiot.* |
| 6.9 | Female ED registar | *Good for teaching, as well. Cause then you can say, well, this is what you're looking for.* |
| 6.10 | Physician | *I think if you can get a reliable image and a reasonable online training of some sort that you can keep accrediting and refreshing. Because I wouldn't see enough of it to really pick something more subtle. And then there's a wide application. And because you can then, I think the advantage is you can take easy videos and photo to then talking to someone about it and then gain experience that way*. |

| **Use of limited resources** | | |
| --- | --- | --- |
| Quote Number | Participant details | Quote |
| Time | | |
| 8.1 | Medical student | *As a student, you learn the right way of doing it and taking your time and doing a full examination. But obviously, there's time constraints in hospital, and you just skip it* |
