# Supplementary material for: Optimising fundoscopy practices across the medical spectrum: A focus group study
Source: PLoS One. 2023 Jan 27;18(1):e0280937. doi: 10.1371/journal.pone.0280937 (PMC9882965; doi:10.1371/journal.pone.0280937)
Supplement: S1 Dataset — (ZIP) [file pone.0280937.s003.zip › minimal dataset/ED_2.docx]

## eFOCUS - ED_2

Facilitator: So, that's recording now.

I will say why we're here today is how you guys use fundoscopy and other clinical skills. Some of the pragmatics you got, getting in there. How it goes into your management protocols and what the barriers are to using it. And what things would motivate you to use it as a clinical skill as you're going.

I want to start with a story for why I got interested in this research. When I was at the Eye Hospital at Sydney, I had a referral, which was an eight-year-old girl who was sent with a letter from her psychiatrist saying, please confirm functional vision loss. She came in with vision of hand movements in one eye and 6/60 in the other, and she presented to four ED presentations, two GP presentations and the psych team.

So, the story was, six-year-old girl, six months prior to anything she had lost her uncle and a cousin in very short succession. That was quite distressing for her. She then had her first ED presentation, which was with headache and a little bit of blurred vision. She had a full neurological exam, nothing was found. They said you're quite distressed acutely, send you home with some routine analgesia. She then had a GP review, which was reported normal, and then back to separate ED, again with headache, tingling in the fingers this time, and again a little bit of blurred vision. Had another neurological exam reported normal, and sent home. Presented to another GP with something similar, and then she was waiting for the bus outside school and a kindergarten kid next to her stepped onto on the road in front of a truck. She ripped this kid off the road, pulled her out of the way of the truck and put her shoulder, did something to her shoulder, in the process and came into ED with a sore shoulder having saved the life of this young child.

So she came into ED with headache, blurred vision, tingling in one arm and in her toes, and a sore shoulder. And they said you strained your arm, her neurological exam was reported normal, and she did have a visual acuity that was still normal at that stage. Still 6/6 in each eye, but they said this is a traumatic episode, you've had two recent deaths in the near family and your quite distressed and this is some form of conversion disorder. The psych team got involved in that stage.

She was then seeing the psych team, but had further headache and a drop in ... She was claiming much worse drop in vision, was starting to bump into things. Presented to ED again, from triage went straight to the psych team who said this is a full-blown psychiatric, full-blown conversion syndrome, this sort of psychiatric episode. Sent her eye review just to confirm this functional vision loss.

When we saw her, so she's 6/60 in one eye, hand movements in the other and she had the worst papilledema I've ever seen in my entire life. By the time she got to the kid's hospital and had her lumbar puncture, her opening pressure was 85. So they needed three manometer's to get high enough ...

Female 1: Oh, my God.

Facilitator: ... to actually catch how high the opening pressure was. She went almost totally blind in one eye and has some residual, 6/36 vision in the other eye, in a young girl.

So, that prompted me to get kind of quite interested in this area, and there's multiple issues with patients presenting through there. I just wonder, having said that story, can we ask around the room what your first response is to that? In the context of [inaudible 00:03:41]

Female 2: That could've been me. I would have sent her home on any one of those visits.

Female 1: Yeah.

And because she doesn't re-present to the same hospital or the same GP, it's far easier to miss her, but it could've been the same hospital and a different doctor every time and the same outcome.

Female 2: And doing conventional fundoscopy is really difficult, let alone on a ...

Female 1: Six-year-old

Female 2: ... on a child. Really hard.

Female 3: I think it would be harder because it's early and she still has normal vision, would you be even seeing anything worthwhile anyway?

Male 5: And would you have dilated someone with normal visual acuity? A six-year-old child.

Facilitator: I would say yeah. A child ... I think the confusing part there is the child also makes it a challenge. Would you guys ... ?

Female 1: If you had an adult come back to the same hospital a third time, you probably wouldn't think too heavily about further imaging? You'd go, okay, they've re-presented, their symptoms have persisted. Even if fundoscopy is the more difficult thing to do, I can, not cheat in a way, but I can go and get a CT scan, and then I can satisfy myself a little bit more. But you think more strongly about how quickly you CT scan a six-year-old. Or am I right [inaudible 00:04:59]

Female 2: For which you'd need sedation [crosstalk 00:05:04] big deal.

Female 1: Even if you take out the radiation, you still have to get them to lie still for 30 minutes, an hour.

Facilitator: So one of the things that struck me, she had five different documented neurological exams and none of them had a fundoscopy in it. They're all de-identified here, but how often do you guys as a ball park figure think in a neuro exam, you would put a fundoscopy ...

Group: Never.

Male 2: I certainly never have.

Female 1: Never, never.

Male 3: Unless neurology asks for it, which is not very frequent.

And even then-

[crosstalk 00:05:34]

Female 2: And even then, I'd do a cursory look and go I couldn't see. [crosstalk 00:05:38]

Male 2: I sometimes tell them to go home and see an Optometrist and get the back of their eyes looked at, and if it's eye-related thing, I do look at the ultrasound.

Male 3: I do find the ultrasound easier to in ED, then the fundoscopy. [crosstalk 00:05:49]

Aussie Male 1: I think it's not something we're used to looking at.

Male 3: If I want to, If I want to.

Female 1: I have to say, when I wore glasses, I found it extremely technically difficult to try and do a fundoscopy. Literally, I couldn't even put it up against my eye, because my glasses frames were in the way. So it was just impossible to use. And even now that I've been wearing contacts for a few years, I still don't do it because it's just too damn hard to do in E.D.

Aussie Male 1: I think the times I've done it, I've done it knowing I was going to speak to Eye Reg and I've always said, I've attempted this, I've no confidence to say one way or another what I'm seeing, I would not be happy to look and make a decision without discussing with not neuro, with eyes, to say, you know.

Male 2: These neuro cases are the most difficult as well cause they're the ones who are gonna be in a big, bright back wall area who you don't want to use extra medical drops on, really, if you don't have to, you don't have time to do everything. Whereas things like detachment, she may see around the corner in edu-care. We've got a bit more time to do those things and, I would certainly with someone with headaches be doing fundoscopy. I know we should be doing, but, I think the majority of that is because I don't trust my skills. Even if I got a good view, I'm not 100% sure that what ...

Male 3: I guess if I considered lumbar puncture, I'll try to get some form of [inaudible 00:07:19] back on the eye. [crosstalk 00:07:22]

Male 2: I look at the ultrasound, but again it's not the most sensitive ...

Female 2: But would you be confident enough, Male 3, on the basis of that to say -

Male 3: On the ultrasound?

Female 2: With your fundoscopy.

Male 3: Fundoscopy, not with our things that hang on the back of the walls, and those don't work. No. But that's why I've changed over to ultrasound.

Female 1: Would you use that in lieu of a CT brain to out-rule [inaudible 00:07:44]?

Male 3: Not in lieu but I'm looking for, instead of consider lumbar puncture, and I'll do that.

Male 2: It all sounds really -

Female 1: You'll just put them on CT anyway? If you're not that suspicious?

Male 3: Yeah. I would be in every that we've got adults, and I think CT is readily available. If I'm concerned enough the headache warrants a lumbar puncture, if it's infection I will dismiss the CT, do the ultrasound, and then do the lumbar puncture.

Female 1: Okay.

Male 2: But I think ultrasound's really good if you've got a gross papilledema. But if it's very early stages, it depends where you're putting your lines, a millimetre either way, it changes what happens.

Male 3: That helps me. Yeah, that's not very accurate. But it will help me to decide whether I can do a lumbar puncture, second of all.

Facilitator: So one of the things you were saying there was interesting, you said you're not too confident with the findings of what you get and therefore you wouldn't want to do it. Does anyone else feel that way?

Group: [general agreement].

Female 4: Or just, I think it's very limited, it's very hard to get really good to use? Even if I do manage to get partial view, I cannot see like at the same time, macular and disc, and just to describe full, whole picture of the eye. You may see normal vessels, or some haemorrhage if you like in that [inaudible 00:09:07] view, but you cannot actually get very good full picture that you could describe well.

Male 3: And the consequences of calling a false negative are so potentially catastrophic as well.

Group: [general agreement].

Female 1: Yeah the practise, it's become something we put in the [crosstalk 00:09:25] for multiple reasons.

Male 4: You get stuck in this cycle of not dilating people because you don't feel confident to look at the back of their eye, and then you're like, well I'm just going to dilate them pointlessly, and then because you don't dilate people, you don't look at many eyes.

Facilitator: And then say they're not happy with their answers.

Male 2: And even going back to med school it was quite difficult to train cause it's, here's this ophthom, he's got this look in his eye, this is what you should see. And I'm sure everyone would just say yeah, I'm seeing that, because you didn't want look an idiot. Whereas, if it's a chest, you can listen to it, everyone can listen at the same time? It's a very difficult and different skill to train.

Female 1: If that, and then you went onto the wards and never did it for six months or a year, and then you came and that's what you did.

Male 5: I got nothing.

Male 3: Yeah, I guess we've got a little [inaudible 00:10:04] aesthetic and put, having a fractional second maybe where you sort of see something, an impression -

Female 1: And that's assuming your patient's [crosstalk 00:10:13].

Male 3: - and the impression you have to rely on, whereas here you can take a photograph and you actually can look at it again and say, look, I think this is actually a papilledema, or with the ultrasound as well you can freeze the picture, measure it, and then you've got measurements.

Male 2: And you can show the other [inaudible 00:10:26] like an ECG, I'm not quite sure about this, what do you think? Rather than, can you come look at this eye as well.

Facilitator: So, do you think that would change your use if you had even just a smartphone kind of like, app [crosstalk 00:10:39] ease of use.

Group: [General agreement]`

Facilitator: You know, you get someone else to look, your med student can go and take the image and show it to [crosstalk 00:10:45] looks like.

Male 2: If it takes 10 seconds, we haven't got any excuse not to do them, really. All our excuses are at the moment that we don't do it often enough, we could do this on every patient. And that we aren't confident, we can practise on every patient; and that it's difficult, but that's not difficult.

Female 2: Yeah, and it saves a lot of time as well, cause what we've noticed, like when we were in urgent care anywhere else, usually when the interns or the residents or, even SRMO's do the eye exams or the fundoscopy, then they come and ask you, and then you have to do it again, as well. So if they can just take the image, then ... it's one less, yeah.

Male 2: Good for teaching, as well. Cause then you can say, well, this is what you're looking for.

Female 2: Exactly.

Male 3: Like in eye week, when we have teaching and we just go through all the interesting images, like we do with the radiology meetings, or [inaudible 00:11:31] radiology meeting.

Male 4: We'll know all the cases, and all the cases would be one's that we've seen, and one's that have come through, and then I guess once we've seen enough, we'll learn enough - basically exactly the same thing that we do with radiology. Cause we see so may chest x-rays, so many ECG's in a shift ...

Female 1: We're happy to peek.

Facilitator: That's brilliant, and we can just follow up with somebody from how, what happened in that patient.

Female 1: I'm sure we probably refer more upstairs than we need to out of, so many fit in the hmm, I'm not quite sure basket, whereas we look at chest x-rays and we go, that's fine, that's not fine, far more ...

Male 2: And we don't really ever get the feedback, we think we pick things up and we send them upstairs and we then never hear back what's happened to this patient and the notes aren't on the computer. [crosstalk 00:12:21]

Male 4: It's a little bit better now that it's online, but before it was online you'd send a whole bunch up and you'd know nothing about what happened to them.

Aussie Male 1: I think most of us, the one's we send up to you guys, we're always looking next couple of days, what was the finding and saying, unfortunately we're not seeing it.

Facilitator: Our protocol with part of this day would be that they'll be a report from the person who looks at it back online. Logistically, we can try and get something directly back to you, but do you think that's enough, if we just -

Group: [crosstalk 00:12:51]

Male 4: We follow up on radiology like, most of us save patients will save patients that we think are interesting, and then ...

Aussie Male 1: Yeah. Everything that we have a doubt about, where we are - well, I think. I have more doubts for you.

[Laughter]

Male 4: I've got a long list of patients that I've saved and I go back over, like -

Male 2: I really like your idea as well as doing like an hour or two in the red's teaching every month and so where you come for a few interesting cases, you'd pick a few, glaucoma's one week and we'd go through them and ... we can actually see what we're doing and how it looks on the screen rather than just reading a textbook.

Aussie Male 1: I do think initially when we're seeing these images, for me, perhaps for others, they'll be less - obviously we will see some pathology, happy to refer, seeing things that we think are normal and saying I'm going to send this patient away without talking to you, I think that would require a little bit more confidence, and that's probably going to be a bit slow. I think that would come with teaching.

Female 2: But the Eye Reg can see our image. You now have a hard copy that you can transmit to the Eye Reg and they can tell you if the patient's safe to go home today.

Aussie Male 1: That's correct, but then it seems that every image that we take in it, it will be seen by the [crosstalk 00:14:01].

Female 2: If you take it on your iPhone or on that, we can transmit it.

Aussie Male 1: Yes, I understand. But is there an intent at some point in the future that this will allow some images to be taken in ED and the patient will be sent home without whoever he refers?

Facilitator: For the purposes of the trial, no, everyone will be looked at just for safety reasons.

Aussie Male 1: Yes. Yes.

Facilitator: Long term, I would think no, just because realistically for our [inaudible 00:14:22] to accept that, they would have to set a H1 as a fine consult, meaning now that you have to be for a fine consult and I think your cost benefit would start to become less.

But, part of that would be the trial with the education package we give to you guys, how confident to feel sending normals, how sensitive is that, what is our false negative right with sending someone home with pathology and do we catch them appropriately. All that will be looked at in the trial, and then from the data we get here, we'll be able to come back and say to the [inaudible 00:14:51] Health, this is how it should go.

Male 4: And is there gonna be a plan for like, a blanket reporting system? So like, for example, with radiology now, admittedly we probably send a lot of patients home who end up having minor things that we've missed, and then it gets formally reported and then they go into a stack and we end up calling patients back or we often call people back with abnormal results, blood cultures that have come back positive, things like that. So will they all go into, like, a reporting that will then ...

Facilitator: For most of the trial, that's what'll happen, and then what we do long term, yes, we'll have something like that happen.

Male 4: So ophthalmology gonna become like the new radiology, where you just have to sit there.

Facilitator: Someone will be sitting there, clicking. [crosstalk 00:15:29]

Well, so that - [chuckling]

The big thing in this is the computer is learning stuff so, like a machine learning algorithm or just analyse a disc and spit it out, they already get 86% sensitivity for diabetic [inaudible 00:15:46] so it's coming for disc swelling and probably if you get one that flags that it hasn't got a sensitive enough result, dyes will be reviewed. This is probably in the long term goal, but in the interim, yes, it's probably where people actually work in H1.

Male 3: I know what you're saying, Cameron, like eventually this may end up being something like chest pain where like a good proportion of them we can say, no, this doesn't need this and that, and we can send them home overnight saying, this is your follow up.

Aussie Male 1: And I think that's a longer term thing here.

Facilitator: In terms of how the process happens, so this is where it's - fundoscopy has fallen completely off, and then when we speak to the physicians, it's exactly the same. Every idiot focus group I've run is like, no one does it. But say you take a chest pain, if you can think back or any of the junior guys if you're more at that point of, where do you start to feel confident saying, no, this is normal, and I can send you home without going through the entire pathway. So that's the kind of question about, when do you feel expert enough to say this is normal, and I can send you home. And what happens in that process?

Male 4: We've had decision pathways that we made, where we just follow them along? And so, that's sort of all based on the evidence and that's been through an agreement with ED and the cardiology department, usually. But there's always the other causes of chest pain and things, so I guess there's always going to be an element of clinical acuity.

Female 1: I guess there's also a safety net as well, at least from a cardiology point of view, because there's a fairly robust follow-up kind of thing, so the rapid access cardiology clinic so that we know that, well, you're fine tonight, you can be followed up in the next couple of days. So, that makes us more confident about now having to admit patients precautionary.

[crosstalk 00:17:29]

Male 4: We split them up into low, mid, and high risk stratification. High risk gets admitted, moderate risk either gets further workup or gets brought back to the clinic, low risk goes home with GP follow up based on risk factors.

Female 2: The other thing is, seeing them over and over and over and over again, from an intern? So you're building your, kind of, clinical [inaudible 00:17:49] as to who needs to stay and who can safely go from day one? Whereas the number of patients that probably should've had a fundoscopy that I saw from an intern is probably far lower, so even if you've done it as frequently as it was required, I don't know - [crosstalk 00:18:09]

Male 5: Imagine if you did it on every headache. Every change, every vague visual change ...

Female 2: It'd be every second patient.

Male 5: Yeah, it'd be like -

Male 4: Every chest pain gets an interview?

[crosstalk 00:18:25]

Male 3: Trying to see nurses, too.

Aussie Male 1: Yeah, so, I said chest pain's obviously a very common presentation, as is headache. The experience base I think you require for chest pain to be getting to a point where a certain portion of them, you're confident to send home without discussing with cardiology, I think comes probably - you probably need to be in an advanced ED training and I think you probably need to be in at least your 81 year which is [inaudible 00:19:00] for what really to be starting to ...

Male 2: I'd say the opposite, I think that when you start studying for your exams, you then send less patients home, cause you find out all these things that ...

Aussie Male 1: [crosstalk 00:19:11] [laughter]

Facilitator: Sorry guys, one at a time, just cause we want to be able to hear.

Aussie Male 1: Sorry, there is a bit of a, you don't know what you don't know, and I suspect that this will follow that same pattern, so certainly it's years of it being a predominant ED presentation.

Female 4: But I think also we've [inaudible 00:19:35] ourselves with the [inaudible 00:19:36] which you could definitely say, oh you didn't have acute coronary syndrome at the moment, and that's why I'm sending them home. Which is, I think, the biggest challenge for me, when you work in the east side, front of the house, especially on Sunday?

So sometimes you'll have six or eight people who just pile up, one after the other, with very non-specific symptoms of blurry vision, of blurred vision. A bit of headache, or tingling here, tingling there, and they're extremely non-specific, just normal examination. so I think those one actually needs to have the fundoscopy and then probably we'll have a bit more sort of reassurance that, okay, I'm going to send you home, I don't know what your symptoms were but, that's fine you can go home. So, I think this is the biggest challenge for me, personally.

Male 2: I don't think we're comparing the same thing, here. I think that a papilladema is you have or you haven't got it, every cardiology patient with chest pain, we're tossing a coin and we're playing the odds. We know we're gonna send a certain portion home who're gonna have cardiac chest pain. Whereas this, if we're trained enough and we're good enough to look at an image, they have or haven't got it, it's not they may have it and it's ...

Facilitator: There are some, so it's not quite as - even the grading scale is a 1-4, and a grade one, ophthalmologists, or newer ophthalmologists will disagree whether it's there or not. So I think there still is a scale on these. The gross ones where I showed you the image and the haemorrhaging and everything, everyone will pick the barn doors. The subtle ones I think it is a bit like the coronary syndromes, where there's your borderlines, but I think from an ED perspective if we can put them into the borderline, the story's not too bad, the vision's okay, from an ED perspective I think that safely puts you into your, okay, send home, get your MRI or EGP in the next couple of weeks to month. And they have a neurology follow up.

Male 3: And hopefully it will reduce that hand-wringing where you're like, well, the CT's, and is this equivocal, and the symptoms are suggestive, but then are we gonna go lumbar puncture this person.

Facilitator: Yeah. Or even do we need to CT or lumbar puncture this person right now, because a CT can miss significant - I had a patient in rooms the other week where a CT was plum normal and then he had an MRI because his optic nerve, his vision was getting worse in both eyes, and he had a whomping great brain tumour with mid-line shift. And it was missed on the CT. Whereas if you hauled off and do the more sensitive investigation as a one-off, not wait 4 hours in ED and not do the LP, if we can pick that safe data subset or help you pick that safe data subset, that'll save patients upset.

Male 2: I think we'll need to, if we're saying that, there needs to be a referral pathway built whatever we're using, then. Because these low risk ones who've got normal, who may have 1 or 2 symptoms, I don't think anybody would be sending those to an ophthalmologist normally, if they've got maybe a little bit of -

Male 3: Or should they see a neurologist?

Male 2: Who should they see, officially, and should they see an ophthalmologist in a month for another check, or ... ?

Male 3: Combined clinic, could be. Combined.

Facilitator: There is a neuro-ophthalmology clinic but it's currently -

Male 3: Different.

Facilitator: Well, it's fantastic for exactly that, but it's currently massively over-subscribed and understaffed.

Male 2: The cardiology clinic is massively oversubscribed, but we have a specific rapid-access one to send our patients to, and we guarantee that they get seen within a certain number of days. And there's the same for neurology, the same [inaudible 00:23:03] as well, so.

Male 4: I think a lot of them just sift and sort, so if they see them once and, they're like okay, whatever, then they refer them for outpatient. Whatever they need and then they go on their merry way.

Male 3: It's true, actually. And then the neurologists who's deciding on the clinic could then look at the report from the fundoscopy, maybe? And say yeah, this is light risk and they can be seen whenever or GP can refer, or ...

Facilitator: You guys in the back, sorry, did you have any other - I haven't heard from you, that's fine. Did you have any other thoughts?

Female 4: No, that's all right. I came a little late, sir, I'm sorry.

Male 6: I think it's the same as everyone's been saying so far. I think it's just, we don't see enough, and I guess it's the decision to actually do fundoscopy in the first place. Because we don't have that sort of knowledge-base you're getting through that experience with it ... yeah, it's the same sort of things everyone else has been saying.

Facilitator: And so, what you were saying before was interesting, and a couple of you were saying that because you don't have the knowledge base and the experience, and you're also worried about missing, sending someone home as a false negative, you don't do it at all.

Is that a common ... like, when people come, would they say they do the same thing?

Male 4: A common example that we go through where we actually progress in our training. So, before you're accredited to do the ultrasounds, or before you've had it signed off on paper, you still tend to sort of float around and do some. But you don't really record your findings because you can't make a clinical decision based on it, obviously unless there's something huge and then you sort of, it gives you a clue. I don't feel confident doing certain things, but I'll still just put it on, have a look around, and see what I see. But I won't document down that these are the definite findings. And I think that's why, even if you try to do fundoscopy, I wouldn't call it either way. I might do it secretly and not tell anyone, I might have a look, 'oh' ...

Group: [laughter]

Male 2: I've had a peek behind the curtains.

Male 4: I've had a few looks before and then just not told anyone that I've had a look because I don't know what I'm seeing, really.

Facilitator: This is something that I think happens far more often - I know through my training, compared - early on as well. We are in the closed doors, none of this goes elsewhere. Do other people think that happens early in your training, or even with other clinical skills that you're doing, you have a look and you think ah, I don't really know, I don't want to commit myself?

Male 5: Oh, very much.

Group: [general agreement].

Female 1: Really pathological, and then you kind of go find someone else to go, oh, now I'm worried.

Aussie Male 1: But this is what we're saying, where either we're talking to somebody because we've seen something or talking to somebody because we don't know. We're not not talking to somebody and sending the patient home. But we are trying ourselves to do that, which I think is legitimate in the early stages.

Male 3: Unless it becomes very evident to me that what I'm seeing in fundoscopy is abnormal, I would not base my further management on that, what I'm seeing, because I don't trust it.

Male 2: I think the neurologists and the ophthalmologists expect that our skills are very good at fundoscopy, and I think if we commit to something that would base that decision on it as well, is what I feel. If I'm saying it looks normal to me, then that decision could be made and I could just have interpreted it wrong.

Facilitator: But off the record, it's probably not safe. If you guys tell us that it's abnormal, we would definitely take that into account. If you say that it's normal, we would kind of hmm ...

Group: [laughter].

Male 2: Maybe you guys, but quite often [crosstalk 00:26:29] neurologist does say, do a fundoscopy, I ring him back saying it looks okay and they go, okay then, we'll see him in clinic in a couple weeks.

Facilitator: Off the record, that's cause the neurologists can't do any of that.

[chuckles] [crosstalk 00:26:38]

- can so the neurologists, when we speak them, say exactly the same thing, it's a nightmare even for them in a [inaudible 00:26:45] and in a brightly lit ED room with ten other people and, other stuff going on.

Female 1: I can't remember a neuro ridge ever coming down and doing a fundoscopy.

Male 3: Immediately before clinicals, they do.

Female 1: Oh, when they're studying for exams.

[crosstalk 00:27:02] [laughter].

Male 4: One of the newer AT's used to always whip it out. They've got no idea.

[crosstalk 00:27:12]

Male 3: Does anybody up here still have their pendoscope that we used?

Male 4: That one actually ...

Female 2: In urgent care.

Female 1: It doesn't work.

Male 3: But did ever anybody ...

Group: [crosstalk 00:27:21]

Male 4: That's the first time I could see stuff.

Female 4: Exactly. It was actually very good but it lasted for a very short time.

Male 4: Sometimes when the patients would come in dilated for whatever reason, from somewhere else, I'd just quickly grab them. I'd be like, let's go have another look. And that's like the only time you ever really got to play with a dilated pupil.

Male 2: Were they trauma patients?

Group: [laughter].

[crosstalk 00:27:46]

Female 1: I'm probably going to end up talking to you. You probably want to assess them before they're dilated. Do I ruin or obscure part of your assessment by dilating them and then sending them upstairs? You lose out of all of that from the start.

Male 4: And then the patient - [crosstalk 00:28:05] - it didn't need anything and then, suddenly this person is walking around like this for 8 hours.

Facilitator: Do other people have the same experience with that? Dilation?

Group: [general agreement]

Female 1: I [inaudible 00:28:15] for that, for that reason, almost than anything else.

Facilitator: Does anyone not have that experience, or is that almost 100% agreement?

Female 4: We just don't dilate.

Male 2: It's the equivalent of almost doing a spec in a female that you don't feel that I want to do this in case you then, oh and do you want to come and do it? And it's the same, I don't want to dilate in case you guys wanted to see what happened before.

Male 4: I made a point of, at some point, I was just like, I really want to learn how to spec everyone. And so whenever the [inaudible 00:28:43] even if it's -

Group: [laughter].

Male 4: - even if it's ... you know what I mean!

Group: [laughter].

Male 4: Patients who are around me, like, they were doing it, I would always go in and I'd be like, can I do that spec and you can go through it with me? And now I spec many more people than I otherwise would. But, maybe -

Group: [laughter].

Male 4: This is recorded, you know.

Group: [laughter].

Facilitator: Do you find then that you pick up more pathology -

Male 4: Yeah, because I've seen more. Because I look inside. Once you've seen, I think a lot of the residents are reluctant? Cause they're just like, if I do a spec I'm just gonna see vagina wall.

Facilitator: So this is a scary thing, but when we do this with the med students, a good proportion said they felt more uncomfortable with fundoscopy than a PR exam. Because you're face to face, you're right up to them.

Group: [general agreement].

Male 4: I thought you meant in terms of ... [crosstalk 00:29:46] confidence.

[crosstalk 00:29:46]

Female 4: I guess it's means, like when you cannot get very good to you and you just start to feel that you've been already so close for some time, and that's why you sort of back off but this is part of it, that's right.

Female 1: Yes, whereas with a PR, they can't see what they're doing.

Group: [crosstalk 00:30:07] [laughter].

Female 4: Eye exam.

Male 4: So then you don't get face on face. You're wearing a mask, it's very detached.

Male 3: Yeah, but I guess also the worst thing you're gonna miss in PR is decreased anal turn and that's really hard to miss. Like it's really obvious when it's present. Whereas eyes, I think it's a bit more subtle, and there's a lot more to know?

Facilitator: What were the motivations to learn properly to do one of those complex exams versus, if we say PR, does everyone feel comfortable that they could do their PR exam -

Group: [cross-talk]

Female 2: It's not complex.

Female 4: Exactly.

Female 1: Almost everything you're going to see is microscopic. There's either blood on your glove, or there's not. There's either haemorrhoids or tags or fissures or there is no tone ...

Female 2: It's fairly reproducible and fairly apparent findings.

Aussie Male 1: It is enforced by many teams as a condition. If you ring them to try to refer a new patient, quite reasonably, and you have not done that, you'd be criticised. Whereas your team, your unit is quite forgiving, I have to say, about - other than visual acuity and things like that, is quite forgiving about ... yeah.

Male 4: I think a PR exam is actually like a technically easy thing to do.

Female 4: Exactly, it's very simple and kind of just tactile, like I do where you just more fumes than you supposed to see.

Group: [crosstalk 00:31:39]

Male 3: The difficulty is learning to be comfortable broaching it and so on, and to be honest, you just do it. You just do it and then it just gets easy.

Male 2: I think it's the interpretation which is the hard ... PR, it's basically putting your finger in a hole and seeing, can he squeeze or is there anything coming out. Whereas a fundoscopy, you've got - the skill's harder to do, and then what you're seeing you then need to interpret in a difficult way. I think having the photo will be so much easier, cause then we can ... you've got time to sit down, look at it, think about it, without someone breathing on you face-to-face.

Female 2: You have enough time to do ...

Male 2: I'll google, yeah.

Female 2: Just google it!

[crosstalk 00:32:17]

Female 1: ... does the patient have pulsus paradoxus. And it was like 1 o'clock in the morning and I said, I can't remember the last time I checked for it. I'm happy to go and have a look and call you back, but I just briefly said to her, I could over-call it or under-call it and that would be wrong, and she said well, maybe I should just come in and check for myself. And then I was talking to the haematology consultant to admit the patient, and we were talking about the pulsus paradoxus, and she said exactly the same thing. I can't remember the last time I've done one, I wouldn't be able to pick it even if I tried. It's inactivity. We don't do it, we don't remember how to do it, and then when we're forced to do it, we're not sure that we clinically interpret what we get. And there's no embarrassment or weirdness about being face to face, it's just I haven't done it in so long.

Male 4: You can do it with an art-line, right?

Female 1: The patient did not have an art-line.

Male 4: I know, but imagine if you had an art-line.

Female 1: I did, I thought to myself -

Male 4: Cause then you could do a number, and then you'd have an objective measure.

Female 1: I did, I thought to myself, if I had an art-line, these would actually be so much easier. But I'm not gonna put in an art-line.

Male 4: Cause then you could graph it on.

Facilitator: So an objective measure that you can back up to would make you more likely to make a call on it, because you've got something backing you up, is that ... ?

Group: [general agreement].

Facilitator: One of the other interesting things that the GP's actually came up with is, because they said the med students go, I don't want to be in someone's face, it's a bright light, I'm there for a long time, so I'm not gonna do it at all. And it's no benefit to the patient, me doing it. So they're less likely to do it overall. For you guys, there's benefit for the patient doing it, so the GP's kind of tended to say, well, if I have to do it, I'll do it. And that being close to the patient is not gonna stop me because I'll go and do a PR which a lot closer and other things like that.

But what they did say is kind of, the things that motivate them to pick exams to do - and this is a complex area, because it's often things that you don't think about - but some people, the thing that motivates them about their medicine is they want to make the unusual call that, I found this diagnosis that was missed otherwise. And sometimes people will say they're motivated by, if they do enough of these exams that other people don't do, that they'll catch things that other people, like you were saying in doing MPB's, you'll catch pathology that other people won't do because they simply don't do the exam.

Does that question ... [crosstalk 00:34:37] make sense? Does anyone want to - you were nodding, do you think you're one of those two, or do you think there's another spectrum for what motivates you for doing exams?

Female 3: I think there's another. For me, I don't want to send someone home knowing that I could have done the exam and I could have caught something. For me it's a safety thing, not because I'm trying to diagnose something, cause I know that I'm gonna send them to their GP.

Female 2: It's a risk stratification.

Female 3: Yeah, exactly. Whether or not they just need to stay in hospital, I need to refer them to a specialist, it's more of a safety thing. I don't want to just send them home.

Male 3: Realistically I think my skills are too bad, I'm not good enough to pick up that thing behind the wall and look at it and make a decision based on what I see. I think that's what it comes down to, because it doesn't take long to do that, but I don't think I can base a good decision on what I see. Only because my skills are not good enough, and I've done it many, many times. Many times. But I still don't see anything.

Female 4: Yeah, like -

Male 3: And this thing is good, it gives you a picture.

Aussie Male 1: And the risk management is pretty fundamental to what we do. I mean what you do is to preserve vision. What we do is to resuscitate, the obviously extreme, and to risk manage the 90% of the other presenters to our service. Anything that helps to reduce the risk, I think we're motivated to do, and we would like to be better at it.

Facilitator: Does everyone agree with that statement? Or have any other comments along ...

Group: [general agreement]

Facilitator: ... kind of a good consensus of what ADA does?

Female 2: I'm trying not to miss the glaringly obvious, not that I'm trying to pick the really subtle. So like, if I listen to someone's heart, I want to make sure I haven't missed the grip for a systolic murmur. If I miss the grade 1 diastolic, the machine [inaudible 00:36:32] I don't really care. So if I look in the back of the eye, I'd feel pretty embarrassed if I sent someone up to you with a massive vitreous haemorrhage that I didn't diagnose, I didn't even look, whereas if I miss a really subtle papilladema ... I was never gonna see that anyway.

Aussie Male 1: Can I mention, other's may not be aware, I've always talked about one of the consultants in another hospital who went through his OSCE exam in about the last eight years. He had a fundoscopy station as a clinical exam, and that's worried me constantly for that reason.

Male 5: In ED?

Aussie Male 1: In the ED fellowship exam, he had a fundoscopy station.

Female 4: Yeah ... that's not good.

Male 2: We have actors now, more than patients.

[crosstalk 00:37:10]

Aussie Male 1: Now whether that's a possibility, I don't know, but it's stuck in my mind for the last couple of years.

Facilitator: One of the big things, when we speak to the physician trainees, I haven't spoken to everyone yet, but that exams are the big motivator for learning something properly. Afterwards, you get CPD motivation for getting things and your intrinsic motivation for doing the best by your passions. But in terms of, as a base line, getting things done well, your exam will push you to ... do you think - [crosstalk 00:37:41]

Aussie Male 1: The physicians are tested more on their examination technique and ability to take actual pathology. I think it's almost been taken out of our exams at clinical examinations, and managing situations is probably more the focus of the OSCE stations, is that ... ?

Female 2: No, they do throw in some exams, they can throw some procedural stations but, like Brad said, they're all simulated, they're all actors, there's no actual pathology to identify. So they can examine you examining a knee, but you're not being tested on whether you've found the pathology, you're just being tested on your process.

Facilitator: So for our exams, you kind of examine the patient and then you ask for auxiliary tests to go with that. Say I want to look at the ECG, they then give you an ECG to interpret.

Group: [general agreement].

Facilitator: So, we're talking to the college physicians about this at the moment, probably once we have some answers [inaudible 00:38:39] because if we want this to go in, I think the technique of fundoscopy is less important cause I can now show you 50 different techniques that will get you started for the answers, but the end result is, it's gonna be super cheap to get a picture of the back of the eye. Probably this will become like you were asking for an ECG. You'll say, this is a patient with a headache, show me the photo of the back of the eye, and ... [crosstalk 00:39:01]

Female 4: Certainly in our written exams, they've given us pictures of fundoscopies and asked us to interpret them, so there might be CRBO or something like that, but you to ... [crosstalk 00:39:10].

Male 2: Well, there's about three images which we need to know well. It's going to be a gross papilladema or it's gonna be their top-hand ones.

Female 4: Pretty much the images that you show us today. So I think like I'm not getting rewarded from exam point of view. Because this is [inaudible 00:39:28] just to review the techniques, only because they ask you like, pretty much whatever you show us today is gross pathologen and recent. But I think it's like, if you work on the floor and you're responsible for the progress [inaudible 00:39:44] then you're surrounded by junior doctors and other [inaudible 00:39:50] and students who look up to you. And you're actually supposed to one who makes the calls and the people just come to you and say, like, I cannot do it. Fix it for me or find the solution. And you [inaudible 00:40:00] in the end, so I suppose this is the biggest motivations to your everything.

Male 2: I don't know if I've ever seen a consultant do a fundoscopy.

Group: [chuckling]

[crosstalk 00:40:09]

Male 2: Maybe one or two, but I think on the whole, if you said well then, tomorrow we've got to teach fundoscopies [crosstalk 00:40:16] then ... apart from maybe one or two of them ... ?

Male 4: I've seen Dr. Unwin do it do a panoptic, she refuses to use the thing on the back of the wall.

Facilitator: If the consultant's in the room, put their fingers in there.

One of the things that the med students - in our focus groups and in all the literature - comes out that a lot of them feel there's active discouragement from doing fundoscopy from their seniors. Do you guys have that feeling, or what's your impression, if you say that you need to look at the fundus, is there any response positive or negative from your ...

Female 1: [crosstalk 00:40:50] does it get in the too hard basket? Or ...

Group: [crosstalk 00:40:56]

Facilitator: Pass the question to you guys first, and then I'll tell you about what we found.

Male 4: If a med student told me they wanted to a fundoscopy and then report it back to me, I'd tell them, well, enjoy, but I'm not going to be able to give you any guidance.

Group: [general agreement]

Male 3: And I would mention that if they said, you know, does this person need a fundoscopy, I'd be like, yeah, sure. That'd be what the textbooks would say, but I wouldn't know how to interpret it. Which would probably be read as discouragement.

Facilitator: And from you guys, speaking to your seniors, then, would you have the same experience or not? Like, if you say that this patient needs a fundoscopy but I'm not confident in my findings, or if you say -

Female 2: I've had a couple of people ask me, usually elsewhere, say - obviously the patient's come in with a headache and things like that, and they've got this and this, and they say have you looked at the eyes? And I've said look, honestly? It's so technically hard to do, I doubt I could see what I need to see, and even if I do see it, it's gonna be absolutely tiny, I'm not gonna be able to tell squat about it. So no, sorry, I haven't done it.

Male 2: I don't think I discourage them from doing it, but it's a test that I'm gonna take absolutely nothing from, because I think in my head I assume that if I can't do it, people my level, and I assume that people above us can't do it very well, I'm not going to take the intern's, what he sees, unless he used to be an ophthalmologist or something.

[crosstalk 00:42:18]

But I think if he said, I think it might be papilladema, I don't know what I would do with that information.

Male 4: I'd do an ultrasound.

[crosstalk 00:42:29]

Male 2: I think I'd have a look, but again, unless it was gross papilladema, which I think you'd be thinking about from the history anyway.

Male 3: There was one time where I asked a guy at my level to do it who's an ex-optometrist, and he was just like, yeah, no papilladema comes to 0.3 and then ... [inaudible 00:42:48]

Group: [laughter]

Male 2: Well that's like, yeah.

Facilitator: Okay, I think that covers most of my things. Does anyone have any final comments or things we haven't raised or anything ...

Male 4: One question. Just with regards to them all being reported as images, do you then want clinical information with each photo?

Facilitator: Yeah, so we won't ask you do that, our med students will go through and get it from your notes and things ... [crosstalk 00:43:12] ... I'm hoping the [inaudible 00:43:13] on you guys isn't too much for the research, cause the idea is the research just comes through, gives us data, and then we use [inaudible 00:43:21] health says we're gonna be sued more often than the cost of getting the camera in.

Group: [crosstalk 00:43:23] [laughter]

Male 4: I guess just from -

Female 2: That's some legit motivation.

Male 4: From the point of view of piloting it, like in terms of in the future if we do do it as a consistent thing, like the radiologists obviously require us to give clinical information, cause when they go to report it they need to know. Would that be something that would be on the ...

Facilitator: It probably will, yeah. So we know that the sensitivity is much higher, you go from about 80% sensitivity to 95 if you give a to-the-line, patient presented with blurred vision, or headache, BP was ... double. A-symptomatic, you'll get much higher sensitivity. So yes, that ...

Male 4: It's true, all of our [inaudible 00:43:58] patients are ...

Group: [crosstalk 00:44:04]
