# Supplementary material for: Optimising fundoscopy practices across the medical spectrum: A focus group study
Source: PLoS One. 2023 Jan 27;18(1):e0280937. doi: 10.1371/journal.pone.0280937 (PMC9882965; doi:10.1371/journal.pone.0280937)
Supplement: S1 Dataset — (ZIP) [file pone.0280937.s003.zip › minimal dataset/Medical student - Year2.docx]

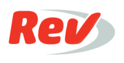


Order

| Client | Hamish Dunn |
| --- | --- |
| Order # | TC0841991331 |

How did we do?


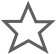

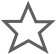

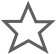

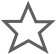

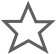


If you rate this transcript 3 or below, this agent will not work on your future orders

Speaker 1: Everybody shuffle their chairs in just a little bit, cause the mics are good, but not fantastic.

Speaker 2: We haven't started yet [inaudible 00:00:07] I think we have [crosstalk 00:00:09]

Speaker 1: I'm just kicking off now [crosstalk 00:00:12]

So basically guys, so this is part of the study that I've given you the info sheets for, and the goal of this study is to, like we talked about with that patient scenario, to pick up what the practise patterns are with fundoscopy and what maybe the barriers there are to doing it, and then what kind of things might about you do to put that into clinical practise with other clinical examination skills. Anything you say today doesn't leave this room, nothing goes back to the college, and your involvement or not doesn't go back to North Shore or anything like that. And you're free to stop recording or leave at any point. Is everyone happy to proceed?

Speaker 2: Yes.

Speaker 3: Yep.

Speaker 1: Good. So if I skip back to start with the story that I talked about before with the six year-old girl. Can I just run round, did anyone have any kind of feedback about that? Or your reactions to that as we go around?

Speaker 4: I think normally before you document anything as a conversion or psychiatric, you'd get the organic things from all that. Like we do it with abdominal pain all the time. We think it's psychosomatic, but let's rule out something surgical and that's probably the same with eyes, maybe rule out that before you go down that conversion ...

Speaker 1: Mm-hmm (affirmative).

Speaker 3: I think a conversion disorder in a child is possibly rarer than the rare eye problem they had, so that was a bit ... I mean in the UK there was a case of it actually, where there was an optometrist who went to jail for missing papilledema. So it's quite worrying because I don't know enough to cure the case, I think it was the not looking at the eyes I think there were other features that were more indicative that there was something wrong with the child. But it would've been nice if you could've been that person for the child who's blind, to say, "Yeah, they are going blind."

Speaker 2: I wouldn't have done fundoscopy on them.

Speaker 3: No, no [crosstalk 00:02:12]

Speaker 4: But I think [crosstalk 00:02:16]

Speaker 3: I think a VA should always be done if there's a symptom of visual ... Like refer them on if the VA was low, then ...

Speaker 4: For neurology to be at least.

Speaker 1: Yeah.

Speaker 4: Or an MRI application ...

Speaker 3: And even though it might be a busy night shift, you can still refer them to a walk-in eye clinic the next day and pretty much any hospital too.

Speaker 2: Yeah.

Speaker 3: And so that's-

Speaker 4: I don't think I'd do a fundoscopy on anyone below the age of about 12.

Speaker 1: Mm-hmm (affirmative).

Speaker 3: So yeah, so that's one of the barriers to that physician, was the patient's age and the time that the patient came in as well.

Speaker 1: Okay. What ... I find that really interesting, so is that consensus no one would do one in that patient? I mean it's easy in hindsight, but if you had a six year-old you wouldn't do fundoscopy as a general-

Speaker 3: Depends on that child.

Speaker 4: No, it's-

Speaker 3: I think it would probably be futile [crosstalk 00:03:01]

Speaker 2: I think that's it, the views you'd get would be-

Speaker 4: So poor.

Speaker 2: Inadequate. You wouldn't pick up a subtle change, which is what we're expecting.

Speaker 1: Hmm.

Speaker 2: And it's gonna be pretty traumatic for the patient and you.

Speaker 1: Right.

Speaker 5: Because in what we're looking for, we usually do fundoscopies if you're looking for a specific thing.

Speaker 1: Mm-hmm (affirmative).

Speaker 5: If we're thinking about raising the [inaudible 00:03:20] wanna rule out ... Like we don't wanna do CT but we wanna do LT, we do it.

Speaker 1: Mm-hmm (affirmative).

Speaker 5: Or if you have a specific question.

Speaker 1: Okay.

Speaker 5: The thing is what were we thinking about this child, have we thought about something [crosstalk 00:03:33] or not.

Speaker 1: Right, okay.

Speaker 6: It has now become less of a part of a general neurological examination, as opposed to part of the eye exam.

Speaker 1: Mm-hmm (affirmative).

Speaker 6: You know, if we see someone with a headache, something else [inaudible 00:03:46] they invariably get something with neuroimaging [inaudible 00:03:48].

Speaker 1: Right.

Speaker 6: And so it's anywhere from saying we've got acute unilateral visual loss, or acute ocular pain that doesn't appear to be traumatic, then it might go down that route. But I don't, I mean it's been a long time since I've done a fundoscopy on anyone with a headache that doesn't have any eye symptoms so ...

Speaker 5: And visual acuity was one of the indications, so I think-

Speaker 6: Yeah. I think-[crosstalk 00:04:11]

Speaker 5: Do a fundoscopy.

Speaker 4: I guess in kids, you often send them ... Like if you're thinking the visions a problem, you'd send them to get their-

Speaker 6: [crosstalk 00:04:20] tested.

Speaker 4: Vision and eye tested.

Speaker 5: Yeah.

Speaker 4: So you'd be like well it's a kid, let's not put them ... It's gonna be hard to do, let's not put them to the trial of it, let's get the eye doctors to do it.

Speaker 2: You're not the reflex [inaudible 00:04:30] you get a VA that's lower than you know, whatever, 6/6, 6/9, you call the opthemologist. And that's possibly a bit of how we work in [inaudible 00:04:40], but in that [inaudible 00:04:41] is where the [inaudible 00:04:42] starts.

Speaker 1: Mm-hmm (affirmative).

Speaker 2: That's a good safety net and I'm surprised that as soon as the patient had VA symptom, if they can't make a formal assessment at least, you'd wanna send them to someone who will.

Speaker 1: Okay. So a couple of things from there. So it sounds like, from what you're saying, that the fundoscopy kind of comes in once you've got a specific eye symptom only? And outside of the eye symptom you wouldn't do it?

Speaker 3: [crosstalk 00:05:12] Very high blood pressure.

Speaker 4: We have some [crosstalk 00:05:16] pressure. No [inaudible 00:05:16] hypertensive-like urgency they just had a single high blood pressure but no other symptoms. And then they felt better, and their blood pressure went down as it always does. I wouldn't do fundoscopy just for that, I wouldn't do it just for a headache if there's no other symptoms.

Speaker 1: Mm-hmm (affirmative).

Speaker 4: I mean I don't know about anyone else.

Speaker 3: It was a headache, so [crosstalk 00:05:36]

Speaker 2: If the patient's unwell, so they're not oriented. This one, we had one the other day, they complained of a scatoma, so then they got fundoscopy.

Speaker 4: It wouldn't be like a routine encounter, it'd have to be something else to make you go down that route.

Speaker 2: But no. If they say, "My eyes are normal" ...

Speaker 1: Right.

Speaker 5: Or neurology, like if you're thinking about optic neuritis in MS, like you've got other neurology that you're thinking about you might do it.

Speaker 2: But how many times does she present [inaudible 00:05:57]

Speaker 1: Six times.

Speaker 2: Cause even one representation for us is usually a, this patient needs [crosstalk 00:06:02]

Speaker 1: Yeah. So just going around the room then, would you say as well that if you're doing a general neurological exam, would fundoscopy be part of that in most of the patients you see, or just in-

Speaker 2: I think it depends where you see them. Because if you see someone in the [inaudible 00:06:20] it's bright, and you haven't got that high [inaudible 00:06:23] suspicion. You're probably not gonna move them over to the eye room and dilate them. But if I seem them in [inaudible 00:06:28] and it's in the eye room, then I'll be more kind of-

Speaker 3: You'll just do it anyways? Yeah sure.

Speaker 1: Yep.

Speaker 2: I think it's very situational ...

Speaker 1: Yeah, okay.

Speaker 4: We rarely do the general, [inaudible 00:06:40].

Speaker 1: Yeah.

Speaker 4: Focus neurology exam on signs of [inaudible 00:06:42], but you're ... I think if someone's [inaudible 00:06:43] you're probably not gonna do fundoscopy.

Speaker 1: Yeah.

Speaker 4: Whereas if you see [inaudible 00:06:48] or maybe hypertension, after imaging you might still wanna look at back of the eye.

Speaker 1: Mm-hmm (affirmative).

Speaker 4: You don't ... Well I don't have to do a general neuro exam.

Speaker 3: A general neuro, yep.

Speaker 1: Who's done it in the last month?

Speaker 4: [inaudible 00:07:02]

Speaker 2: [crosstalk 00:07:05] Yeah, the patient had a BP of 235 [crosstalk 00:07:07] But again, it's not even so much, I can get a view. I'm just not always sure whether it's [inaudible 00:07:14] or not.

Speaker 1: Mm. Of those people that put their hands up, can you just tell me ... So there's a case of BP 235-

Speaker 2: He has a hypertensive crisis with a scatoma and visual loss, so it was like pretty much as soon as we finished the exam, [inaudible 00:07:29] with that. But again, I wouldn't do it in a patient who didn't have those symptoms.

Speaker 1: Right. And did you find anything or did that [inaudible 00:07:38] change?

Speaker 2: There's no [inaudible 00:07:38] but I ... This was novel, but we called the [inaudible 00:07:43] they found a branch, but I don't think that changed their management. But it changed their follow up.

Speaker 1: Yep.

Speaker 5: And [inaudible 00:07:53] patient, so-

Speaker 1: Mm-hmm (affirmative).

Speaker 5: It was more basically history, but I tried to have a look at him, but I couldn't see anything that was [inaudible 00:08:00]

Speaker 1: Mm-hmm (affirmative).

Speaker 5: But basically history, I was concerned enough to talk to neurology anyway, you know?

Speaker 1: Mm-hmm (affirmative). Who else had seen one in the last month that-

Speaker 4: Floaters.

Speaker 1: Yep.

Speaker 4: Not that interesting.

Speaker 1: Yeah, sure, and could you find anything?

Speaker 4: No, I couldn't see anything but [inaudible 00:08:16] they could see.

Speaker 1: Right.

Speaker 4: The small vitreous haemorrhages I couldn't.

Speaker 7: I had a headache with hypertension.

Speaker 1: Mm-hmm (affirmative).

Speaker 7: Again, I think it's difficult unless [inaudible 00:08:34]

Speaker 1: Right.

Speaker 5: [inaudible 00:08:37] month ago. That was sudden vision loss after straining.

Speaker 1: Mm-hmm (affirmative).

Speaker 5: It wasn't normal, it was something that [inaudible 00:08:44] so I sent it to them.

Speaker 8: I had a haemorrhage [inaudible 00:08:52] I didn't know what I was looking at, but I knew it wasn't right. Then I picked up a [inaudible 00:08:58] vein occlusion about a month ago. By looking, you know, someone with a acute universal vision loss. It looked very weird-

Speaker 1: Mm-hmm (affirmative).

Speaker 8: In the eye [crosstalk 00:09:10] that it's like this is not normal. I'm [inaudible 00:09:13]

Speaker 1: Good. You?

Speaker 9: I'll sort of go ahead. He had a vitreous haemorrhage, it just looked weird. It just looked weird and abnormal, and I was also dilating him in the eye room, and the [inaudible 00:09:24] was already aware, so it was like [inaudible 00:09:28]

Speaker 1: Right.

Speaker 9: That was obvious.

Speaker 1: So some of the things I'm hearing, and just tell me if I'm wrong here, that there's some ... In the main room it's quite bright, you've got to think about dilating, and time pressure is a massive thing you need, are they some of the main-

Speaker 4: [crosstalk 00:09:40]

Speaker 2: Yeah, exactly.

Speaker 1: Right, okay. [crosstalk 00:09:44]

Speaker 3: I took one off the wall the other day, took the visors off and walked and put it into another cubicle to use it and that did worse than good.

Speaker 2: I mean I would have to just say that [crosstalk 00:09:57]

Speaker 5: Even the visual acuity sometimes, we get the patients that we don't have a [crosstalk 00:10:03]

Speaker 1: I'll show you two things after this [crosstalk 00:10:08]

Sorry guys, for the recording if you can go one at a time, we can hear what you say after.

Speaker 4: Sorry.

Speaker 2: Who hasn't spoken?

Speaker 9: I had a patient fast-track this week or last week, who now I'm sort of wondering if I should've fundoscopy ... I [crosstalk 00:10:29] this conversation. He had had six weeks of what sounded like blepharitis, and had improved and then in the last two days gotten worse again. The GP had said to me to check his visual acuity at 15/6, but with [inaudible 00:10:45] corrected to 6/6 on one eye and 6/7.5 on the other eye. So I wasn't actually concerned about the visual acuity, which the [inaudible 00:10:54] we didn't even discuss fundoscopy at any point. And then they were gonna follow up with the ophthalmologist that week, so-

Speaker 3: That's reasonable, yeah.

Speaker 6: Can I just say one of the things about dilating the eyes as well is that my general understanding, and it seems to be in some of the eye manuals to this day, is that you shouldn't dilate the eyes until you've been told to do so by an ophthalmologist.

Speaker 3: So I think it should be less than that. There's a new eye emergency manual-

Speaker 6: Yeah.

Speaker 3: Up now, we just edited it and it's just been released again now. So it's much more interactive, got a lot more detail, so I'd suggest you all download it. But the dilation, as long as I think that those two things ... Like if you've got one going neuro concerns and you're to monitor them then you can't dilate anyway. And if you think they're at massive risk of angle closure, so that's Southeast Asian, [inaudible 00:11:48] so their eyes look massively big through their glasses or they've got a family history of angle closures then I probably wouldn't. But otherwise you're quite safe to dilate outside of those. Yeah, and it's kind of the ones where you're not too worried. Like where you're thinking, "Can I get this person out of here without seeing the ophthalmologist, and they can see them in the next month in clinic", then I think dilating on your own is fine.

Speaker 7: On that note, I've been using tropicamide quite freely.

Speaker 6: I [inaudible 00:12:17]

Speaker 7: Yeah, the problem is disposition, so you usually have to ask if they drove there or not. But isn't it ... There's not really much point in doing fundoscopy if you haven't dilated them. If you're gonna get-

Speaker 3: With the direct it's pretty hard to get-

Speaker 7: Really inadequate views.

Speaker 3: Yeah, you're probably not gonna screen.

Speaker 4: And it's a time issue as well. Like if you've got four eyes in fast-track you're taking turns to use the eye room. And you're just like that I'm gonna dilate him, that's 40 minutes waiting for it to fully kick in. Also once you've dilated them then they have to come back out and sit in the fast-track chairs as their eyes slowly dilate and their slowly getting more ... And there's nowhere dark to put them to wait, you know. And then you really get four hours if you pick them up for two hours you wait an hour for the eye room to be free, you dilate them ...

Speaker 7: There's your four hours gone.

Speaker 4: Yeah. I know that's a very mechanical thing, it's not ... You're never thinking just about time, but it is a factor.

Speaker 1: Yeah. Are there any other ways time plays into things with this? Like when you were saying that you do a very focused exam, is that time playing into that, or what else comes into what you choose with your examinations that you ...?

Speaker 9: I think it's what you're looking for.

Speaker 1: Mm-hmm (affirmative).

Speaker 9: So maybe any actual vision loss I would examine the eye, but ... cause I would do it generally on a neurological exam. But yeah, I think it's just ... You have a kind of idea of a couple diagnoses in your head and things that you're looking for. And yes probably in the interest of time it's also [inaudible 00:13:47]

Speaker 1: Mm-hmm (affirmative).

Speaker 9: [inaudible 00:13:50]

Speaker 1: Okay, anyone else? Do you think time plays into ... Or has time interacted, what clinical examinations you'd do?

Speaker 2: I think with getting the pan-optic and dilating them, if there's a [inaudible 00:14:07] you are much less likely to do all of that if you've ... Where as if it's five, four, and there's no one waiting because the other afternoon teams come on, you're probably more likely to go and dilate and get the pan-optic. Which is [crosstalk 00:14:19]

Speaker 5: The [inaudible 00:14:22] locked in the tower-

Speaker 2: Oh, getting out to fix the-

Speaker 5: If you need to [inaudible 00:14:23] the number, if you need to request it and then getting it [crosstalk 00:14:29]

Speaker 6: But it's not even like that, sometimes it's not there and there's no battery.

Speaker 2: Yeah.

Speaker 6: Then you [crosstalk 00:14:43]

Speaker 4: I think if the ... Sometimes that eye [inaudible 00:14:55] in our clinics if you call them from fast-track.

Speaker 6: Mm-hmm (affirmative).

Speaker 4: I feel like I'd be less inclined to then dilate them and do all of that if they're gonna [crosstalk 00:15:06]

Speaker 1: If you're ... One of the things I was looking at in kind of decision making is just how we make those ideas what we're looking for. An ED being more time pressured you kind of either tend to go for pattern recognition. You know if someone comes in with a certain pattern of symptoms, you're already thinking one particular diagnosis and run down that. Or you have no idea what's going on so you run a more general cover top to bottom on every examination. Or you think, "I'm gonna rule out the worst case scenarios", another common ED approach to things and those help us get through in time. Do you think ... This is a complex question, then. Where would you put fundoscopy in your ways of approaching patients? Like is it in your ... It sounds like people are putting it in once I put you into the "You're likely to have an eye problem", that's when I'm gonna out my fundoscopy in.

Speaker 4: Yeah.

Speaker 1: Does it also fit in your worst case scenarios minds? Or-

Speaker 8: They rarely give me a diagnosis that I hadn't thought of.

Speaker 1: Right.

Speaker 8: It's like I look into my [inaudible 00:16:19]

Speaker 1: Yeah. [crosstalk 00:16:23]

Speaker 6: I certainly don't do it as a general thing if the patient hasn't got symptoms.

Speaker 1: Yeah.

Speaker 7: It seems to be all I could say was, "It looked weird."

Speaker 1: Right.

Speaker 7: And that doesn't really help you if you don't know what you're looking for.

Speaker 1: Yeah, sure.

Speaker 3: Yeah, I was going to say it's a rule-in, not a rule-out.

Speaker 7: Yeah.

Speaker 3: I would not call something from fundoscopy with my level of skill. I wouldn't this certainly is not [inaudible 00:16:41] based on what I've seen in their eye.

Speaker 1: Right. Does that rule-in versus rule-out ... Again, a difficult question to generalise, but as a rule-in versus rule-out thing are you more likely to do other clinical examinations because that would rule things out for you rather-

Speaker 2: Of the same tune?

Speaker 1: Well, thinking examinations first as a ... Like say otoscopy is using a similar kind of piece of equipment. Are you more likely to use otoscopy more frequently than you use-

Speaker 4: Yes.

Speaker 6: Yes.

Speaker 7: Yes.

Speaker 2: I just think it'd be easier. [crosstalk 00:17:12]

Speaker 1: Okay. So is it, going around the room again, you guys have both said it's easier-

Speaker 2: Yes.

Speaker 1: And that would probably make it more likely, would you agree that that's-

Speaker 2: It's readily available as well. It's on every wall, and just like [crosstalk 00:17:22]

Speaker 6: Probably look in someone's ear most days.

Speaker 4: Yeah.

Speaker 1: Right. [crosstalk 00:17:26]

Speaker 6: But we can competently say that they do have a thing based on [inaudible 00:17:27]. They do not have this when looking in the ear, or it's much like you said with a [inaudible 00:17:35] you might say just cause I haven't seen it doesn't mean it's not there.

Speaker 1: Mm-hmm (affirmative).

Speaker 9: And it's faster to use I guess, and easier to use even in kids and [inaudible 00:17:42]

Speaker 8: And it more often gives us an answer.

Speaker 5: In kids it's part of the neuro exam- [crosstalk 00:17:48]

Speaker 4: Yeah, you do it for every patient anyway.

Speaker 5: Every patient.

Speaker 8: Right, and it's easier isn't it? I mean [inaudible 00:17:54] but when you look in the ear you can sort of see what's going on. Is it inflamed? Is there a hole in the ear drum? Is it not inflamed? And then [inaudible 00:18:04] and there's no other way of making that diagnosis.

Speaker 1: Hmm.

Speaker 8: Like people say, "My ear hurts", and they've got maybe some gunk in their ear, maybe not. You have to look in the ear, otherwise ... That really gets the diagnosis.

Speaker 1: Right.

Speaker 8: Where as with the eye you can sort of [inaudible 00:18:21]

Speaker 1: So you're saying ... Now correct me if I'm wrong here, but it sounds like you're kind of saying three things there. So one thing is its easier to do with otoscopy. Two is you feel happier that you can competently make a call on what you're finding there, and that will change your disposition for the patient. You can rule something in or rule something out more confidently. And the third thing I've completely forgot what it was gonna be ...

Speaker 8: I guess it's also the way, the person you're referring to. I think the EMT, when you call EMT ... A, we do it anyway, but the expectation is that you have always looked at it, whereas I think we rely on ophthalmology registrars a lot more than we rely on [inaudible 00:18:58] registrars to rule out concerning diagnoses. The interior shot isn't often concerning the diagnosis that we have thought of-

Speaker 1: Already thought of, yeah. Okay.

Speaker 6: I used to get the sensation that the regs summarily discount whatever you say. I think they do assume, probably correctly, we're not skills or competent enough to look in the back of an eye.

Speaker 1: Yeah.

Speaker 6: And we say it to them they're like, "Whatever. What's the visual acuity?" If the visual acuity's normal they get seen in a few days in eye clinic, if it's not they come down and see them. It seems to be my experience of what the ... Not being rude, seems to be the-

Speaker 4: No that's, and that's fair enough, cause that's where we feel our skill set is. We feel our skill sets requires, even as a senior reg. level, requiring supervision for this skill. [inaudible 00:19:47] not competent to make decisions on it. And if I see something that's horribly abnormal and I was already thinking that anyway, in which case the patient's already gonna be [crosstalk 00:19:53]

Speaker 2: Our college [inaudible 00:19:59] learning these analyses does not imply that, it implies that by the time we finish our training we should be [inaudible 00:20:03]

Speaker 9: We look in ears more than eyes because it will change our management. So I make a call based on if I see an eye if [inaudible 00:20:13] where as an ear will change it. I think if you look [inaudible 00:20:18] we're less experienced, therefore it just ... Everything will add up.

Speaker 1: Okay. If we take, so I know you didn't get much of a chance to play with things and it was without the app so they weren't working optimally, but say I was to give you a photo of the eye that you could get in ... So we train second year med students who've never done this before. I can train them to, within two minutes, get a picture of the eye that they can send off. If I could tell you with your find I could give you a picture in two minutes that you can either analyse yourself or send to the eye reg., would that change the frequency with which you do the examination?

Speaker 3: Yeah.

Speaker 4: Yes.

Speaker 9: Yeah.

Speaker 8: Might decrease unnecessary referrals as well, to eye reg. [crosstalk 00:20:58] Sorry.

Speaker 4: No, go on.

Speaker 8: I kind of like the idea coming back to it, as well. It's sort of nice that you can prove what you actually saw and then ...

Speaker 3: But, if we have access to something you can use it more. For example the pacemaker check we have. In the district hospital you don't have the pacemaker check again. It's not as important, but when you have it here you use it a lot more, if someone's got a pacemaker I'll use it a lot more.

Speaker 1: Right, and as an example how much more frequent do you think you've found pathology with a pacemaker check?

Speaker 2: [crosstalk 00:21:36] You can avoid admissions. [crosstalk 00:21:39]

Speaker 3: The other option would've been admitting medics and then they'd have eventually done the pacemaker interrogation. But because it's point of care, we get a sample from the pacemaker company within 20 minutes.

Speaker 1: Right, okay. [crosstalk 00:21:52]

Speaker 3: Yeah, yeah. So you've got something kind of new with dizziness or collapse, and they basically got a loop recorder in 24 hours a day, you get a report, you know it's not a cardiac arrhythmia, which is a big differential you can get rid of. So it's not a case ... We've all picked up the odd case of something weird or somebody being v-fib or whatever, or the pacemaker being broken. But it's that why I use it for. Cause the patients that have pacemakers invariably have stuff going on with their heart, by definition.

Speaker 1: Yeah.

Speaker 3: So you can discount out wide differentials straight away.

Speaker 1: Yeah, okay.

Speaker 9: I think it would change my opinion, cause having a picture you can look at and study with another colleague, and take your time about is so much different from looking at the eye and doing the quick, "Oh goodness!" And then trying to process all that information yourself. Whereas now you've got a picture that ... So hopefully this will also get better at, you know we can look at the picture, then look at the eye book and compare. And if not we'll probably still be speaking to opthalmologists about the same cases, we'll have a bit more information for them and I think it will change the disposition, and it might mean they get seen in a few days or they get seen straightaway, like that might change a bit. The eye clinic might get a bit less full.

Speaker 4: Probably describe what it looks like rather than say, "It's weird".

Speaker 9: [crosstalk 00:23:00]

Speaker 8: And it's really good learning [crosstalk 00:23:05] it's like an ultrasound, everything else that we do. Then you find out later on that they've got X-Y pathology three days later, and you know what it looks like, and you've seen it there.

Speaker 1: Mm-hmm (affirmative). So one of the things the med students highlighted was as they're learning these techniques, feedback. So say for example we're doing a cardiac osculation, as you're doing it you know your registrar or whoever's watching you can watch what you're doing, say, "No, put your stethoscope here or there and listen to this", and someone else will listen to it and give you feedback. They were saying universally that does not happen with fundoscopy. Is that your experience, or do you think [inaudible 00:23:44] and feedback with learning fundoscopy is different?

Speaker 5: There is nobody that helped with us usually [inaudible 00:23:55] or there is no ophthalmology registrar for us to kind of look and say well what [crosstalk 00:23:57] usually very busy, and if you're doing stuff in a routine one, you don't get other people's thoughts supervising.

Speaker 1: Right.

Speaker 8: I think [inaudible 00:24:08] has quite a lot of similar issues. I think we [inaudible 00:24:10] the confidence of the trainer has gone up and the trainee, when you see the work that you're actually seeing. So we have a screen on one side, you are still doing the training. Earlier we were like panicking with the trainees, "What do you see, tell me what you're seeing!" We all come in that room-

Speaker 1: Right.

Speaker 8: And the whole event of [inaudible 00:24:35] has become a little bit of a pleasurable experience that [inaudible 00:24:37] It has changed. So [inaudible 00:24:42] is very important, I think.

Speaker 1: Yep.

Speaker 8: And this is what is going to blend into our world, someone is able to look at you while you're training.

Speaker 1: Yeah.

Speaker 9: Definitely it'll save some time if you have a junior who's looked in the patient's eye, the registrar has to go in and look at the eye, and then the ophthalmologist has to go in and look at the eye as well. Whereas if you have a picture-

Speaker 4: You're more like looking at an EKG.

Speaker 9: Yeah.

Speaker 4: And the patient's [crosstalk 00:25:08]

Speaker 2: And you can get a consensus from consultants. [inaudible 00:25:18]

Speaker 1: And the other thing that came out of a lot of the med student focus groups was the discomfort to the patient during the examination. They said it's a bright light, you're in their face, you're there for a couple minutes at a time doing it. Does that come into your thinking as to whether you would do it or not?

Speaker 6: We do it when we must.

Speaker 4: Yeah. [crosstalk 00:25:40]

Speaker 1: Interestingly GPs were exactly the same, the discomfort wasn't an issue.

Speaker 6: No.

Speaker 4: No. I do think-

Speaker 8: I think using these instruments including a [inaudible 00:25:54] including an ultrasound probe, brings a lot of comfort to the patient saying they're doing things for them. Not just chatting with me and making their ... They're actually physically coming close [inaudible 00:26:06] An ultrasound probe, it's been proven that the customer satisfaction is higher than [inaudible 00:26:11]

So I think having that kind of ... Even for the sake of, something where I can't see anything I still pick up [inaudible 00:26:20] and attempt to do it because I want to look like that I'm examining. And they feel it! They think that you're a good doctor.

Speaker 7: It's like physicians and all their blood tests. [crosstalk 00:26:35] I mean I'm terrible at [inaudible 00:26:41] but they're worse [crosstalk 00:26:47]

Speaker 2: They're doing undilated fundoscopy. [crosstalk 00:26:52]

Speaker 7: I genuinely think they do it to make you think they're looking, cause there's no other way-

Speaker 6: You're wrong.

Speaker 4: In case you've got a branch.

Speaker 1: Does anything about ... So the other thing that came out of the med students was the fact that it's a face-to-face examination, so they're quite ... And the funny thing is quite a lot of them said they're actually more uncomfortable doing a fundoscopy than doing a PR examination because you are face-to-face with the patient when you're doing it, and that's too much. [crosstalk 00:27:13] An unusual bunch, it's the one's who volunteer for focus groups [crosstalk 00:27:18]

Speaker 8: They don't know what they're seeing.

Speaker 4: Yeah, yeah, yeah, so they're saying any time you're doing something you don't know what you're doing you don't want the patient to know. And so if you clearly can't use the [crosstalk 00:27:29] So when you get new equipment you're like, "Just one moment".

Speaker 9: [crosstalk 00:27:35] Which foot? [crosstalk 00:27:38]

Speaker 7: You just learn [inaudible 00:27:39]

But traditional fundoscopy [inaudible 00:27:50]

Speaker 2: I think it's a bit like doing a skull x-ray. [crosstalk 00:27:56]

Speaker 9: They don't tolerate well from pain, especially if they've got a headache and you're shining this bright light right in their face.

Speaker 1: So that intersection, now correct me if I'm wrong, but there's a kind of intersection where fundoscopy drops off because several things; one, we don't get good training, we don't get good feedback, as a result we're not confident in that we'll be able to interpret and rule in or out a diagnosis on the basis of what we're seeing. And it also is currently falling into only a subset of patients where you think this is just an eye problem, and I have time in a busy department where I could take them outside where they normally are to the eye room to get things done properly with a dilated exam. Is that a kind of rough summary of stuff we've talked about?

Speaker 9: Yeah.

Speaker 2: Yes.

Speaker 1: So our thoughts about the solution for this, and any logistics ideas or ED feedback for this is good, we need to address some of the technical barriers to get around it being difficult. So if we can make it either, the patient comes in and if they have one of those things that we talked about, a headache or vision loss or neuro things they're gonna have an eye photo stuck already in their file, and you just need to look at it as a bunch of looking through things. Or, if we can't convince that it's [inaudible 00:29:13] to buy a camera then you have a smartphone that we can train you in how to use it. Making the assumption that I've trained med students I can confidently say that in two hours I can make you comfortable to get a photo of the back of the eye in two minutes per patient that you could then stick in the medical record, and we'd train you online and how to interpret that. Do you think that would make a difference in how you used it?

Speaker 2: Yes.

Speaker 4: Yes.

Speaker 3: Yeah.

Speaker 5: Yes.

Speaker 6: Yeah.

Speaker 7: Yes.

Speaker 8: Yes.

Speaker 9: Yes.

Speaker 7: I think logistically if you could get a patient ... If a patient gives a symptom of visual loss, if like you said [inaudible 00:29:47] actually could get a VA measured and a photo of the rest of it by the time you saw patients.

Speaker 1: Okay.

Speaker 7: [inaudible 00:29:58]

Speaker 1: The triage would [crosstalk 00:29:57]

Speaker 7: Yeah, that would be ... That's [inaudible 00:30:01] good disposition, if you could see them and then if you're worried you send that image onto the ophthalmologist and in terms of flow that would be the optimum flow of getting that patient through the ... Which probably inquiries turn-over and billing of the managers [inaudible 00:30:15]

Speaker 1: Yeah.

Speaker 9: Does the [inaudible 00:30:16] do the pressures as well? Cause it looks like [inaudible 00:30:20] on the picture.

Speaker 1: No, it's a separate machine to do the pressures.

Speaker 9: Oh, [inaudible 00:30:24]

Speaker 2: [crosstalk 00:30:26] Having something in the records like you said I think is really increasing medical, legal, society. It's good.

Speaker 4: And [inaudible 00:30:33] that you mentioned that picked up the 14 percent of patients that have something they found in fundoscopy that changed their management, was that when the ... That's when an ophthalmologist interpreted it?

Speaker 1: That's with the camera.

Speaker 4: So who interpreted it?

Speaker 1: Oh, when the ophthalmologist interpreted it. Yeah. I can explain that to you, it might be at the end of the focus group cause that probably answers a bunch of the questions.

Speaker 4: I'm just wondering why the-

Speaker 1: 67 percent once the ED guys were trained.

Speaker 4: Right, okay. [crosstalk 00:30:59]

Speaker 1: So, yeah. So they picked up ... You won't pick up 100 percent, but you'll pick up 67 percent of that pathology.

Speaker 4: Right.

Speaker 9: Wow.

Speaker 1: So, versus zero. So it's not 100 percent, but it's better than zero. The last thing that's come up a lot with the med students, and we'll have to ignore the fact that the [inaudible 00:31:17] So I won't mind, but is there a barrier from ... So the med students said they were actively discouraged from doing fundoscopy, or it was kind of ... When they said, "Oh, when I need to do a fundoscopy in this patient", that their [inaudible 00:31:32] you're not gonna get an answer out of that, don't worry. Do you find there are any barriers from that kind of thing [crosstalk 00:31:39]

Speaker 6: People are pleasantly surprised when you've done [crosstalk 00:31:41]

Speaker 7: I think that's probably the medicals too.

Speaker 1: Right.

Speaker 7: Cause if a med student said to me, "I won't do a fundoscopy" I'd say "Okay, well you have a look, but you probably need to dilate the eye, and the patient probably doesn't need-

Speaker 2: And they're probably doing it for after [crosstalk 00:31:51]

Speaker 7: Inappropriate dilating their eyes just being to examine them.

Speaker 1: Yeah.

Speaker 7: And that's probably maybe by their thinking.

Speaker 1: Right.

Speaker 3: But if we're doing it with a sort of cause, we're just left to get on with whatever tests we think are appropriate.

Speaker 1: Right.

Speaker 3: So then we will do them and do it. I've never had anyone tell me to not do a test, probably ever.

Speaker 9: Medical students are just saying that because they want some guidance or training. And we're all sitting here going, "Oh, well we're all full of this". We're not really gonna go, "Oh yeah great, you go look in the eye, then I'll look in the eye, then we'll talk through it together." [crosstalk 00:32:22] I think that's the problem.

Speaker 1: Okay, great.

Speaker 4: I'm from a different system where we have less access to imaging and so I think, actually my fundoscopy's dropped off the face of the Earth.

Speaker 1: Hmm.

Speaker 4: Not that it was ever any good, but since I came to this system I think possibly there is a whole shift top-down to not do fundoscopy so much, and not rely on your findings in the ED. Maybe it's a good thing, because maybe we're [inaudible 00:32:53]

Speaker 1: So, [inaudible 00:32:55] was it I'm guessing?

Speaker 4: Yeah, in the UK. Yeah.

Speaker 1: Yeah. And you were doing fundoscopy to help rule-in or out imaging?

Speaker 4: To make decisions about whether we needed to image people or not before procedures. [inaudible 00:33:06] or even just [inaudible 00:33:08] access a CT scan.

Speaker 1: Mm-hmm (affirmative).

Speaker 4: So I think we did a lot more of it and relied upon it possibly erroneously.

Speaker 1: Mm-hmm (affirmative).

Speaker 2: So they had consultants that would ... If you hadn't [inaudible 00:33:19] they would come for you.

Speaker 4: Yes.

Speaker 2: They wanted to know why you hadn't-

Speaker 4: And then they'll phone eyes or neurology and they'll say we're not [inaudible 00:33:24]

Speaker 2: Yeah.

Speaker 4: And then yeah, you probably get that as well.

Speaker 2: Yeah we do get that all the time.

Speaker 1: Would you say, anecdotally, from ... How often did that change your practise in doing more fundoscopy at home?

Speaker 4: Pardon?

Speaker 1: How often would you find something and, like a papilledema when you were doing it more back home, and would it change [crosstalk 00:33:44]

Speaker 4: I would probably go to people to ask them to look if I wasn't sure.

Speaker 1: Okay guys, that answers most of my questions. Does anyone have any other things they'd like to say, or feedback about anything?

Speaker 7: What would you have sent through the app to an ophthalmologist, the pictures we have?

Speaker 1: Yes, yeah.

Speaker 7: And is it gonna be someone that's on-call all the time?

Speaker 1: So, I'll probably stop the recording cause that's the end of the-

How did we do?


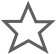

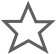

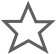

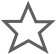

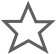


If you rate this transcript 3 or below, this agent will not work on your future orders
