# Supplementary material for: Optimising fundoscopy practices across the medical spectrum: A focus group study
Source: PLoS One. 2023 Jan 27;18(1):e0280937. doi: 10.1371/journal.pone.0280937 (PMC9882965; doi:10.1371/journal.pone.0280937)
Supplement: S1 Dataset — (ZIP) [file pone.0280937.s003.zip › minimal dataset/Medical Student - Final Year_2.docx]

## eFOCUS - Final Year Medical Students

Facilitator: So the purpose of the focus group, we'll go for about, somewhere between 30 to 40 minutes. I don't want to leave you guys over if you need to get out elsewhere, so whenever you need to leave is fine. We're mainly looking at getting your opinions about learning new clinical skills, and about your experience of fundoscopy. What I might do is start with a story of why I'm particularly interested in this thing, and then get some responses from you. All your inputs are really valuable, so anything you have to say is good. If you want to take it in a different direction or add extra information, that's really valuable.

One of the reasons why I got interested in this is when I was at Sydney Eye in emergency there, we had a referral of a six year old girl, and I had a letter from her psychiatrist, which said "Could you please confirm functional vision loss?" Which means your vision system's normal but you're just [crosstalk 00:00:53], yeah, pretending to be blind. And this was a girl who she'd lost her uncle and another family member in a short succession, and then she started developing, complaining of vision loss, and then had presented to emergency once, had a neurological exam, which was said to be normal. They'd mentioned maybe this is a conversion syndrome. She was sent away. Saw a GP in the intervening time, and then she was standing waiting ... sorry, one of her close friends at school died shortly after that. So there's three family deaths within a short space of time.

Went back to emergency, a different emergency department, second time again had a neurological exam, which was reported normal. They said probably more likely to be conversion with a third death, and referred her to the psychiatric team. Went home, she went to school, and standing in the line at the after school and a younger child stepped in front of a bus, and she pulled this child off the road, saved the child's life, but put her shoulder out doing it. So went into emergency department again complaining of vision loss, bit of headaches and tingling in the fingers, and arm pains. And they said this is all conversion syndrome. Had a neurological exam, which was reported normal, and then they sent her to us. She was 6/60 in one eye, and perception of white in the other eye, with the worst papilledema I have ever seen in my entire career. Horrible, horrible. By the time she had her lumbar puncture she had an opening pressure of 85. Which is just through the roof. And irreversible vision loss in one eye, and almost, not quite legally blind in the other eye but severe vision loss in the other eye.

That was, in the end, five presentations to emergency and a presentation to the GP, where she'd had a reportedly normal neurological exam but no one had looked at the optic disc. We know this is a challenging thing to do. It's technically difficult to do and it's hard to interpret the results. But I just might ask, what's your response to a story like that? Just want to go around briefly? [inaudible 00:03:00] you were nodding-

Speaker 1: I think- Examining a child is firstly difficult. That presents unique challenges. I imagine doing fundoscopy in kids, engaging with them and actually being able to perform the skill, it's two unique challenges.

Speaker 3: I guess one of the things is that we don't have ... with majority of us I guess going into GP later on, and you did say that she did have contact with a GP. We don't get much teaching, really, in medical school with ophthalmology, and in particular, fundoscopy. So, I guess one of the most important skills would be that. But we don't get much exposure to it.

Speaker 4: Well, I think it's just this whole case ... I think it's kind of shocking in some sense, now that she's presented five times to emergency as well and nobody actually did fundoscopy to see. I mean, if it was caught earlier, it would be reversible. Definitely exposure to use of fundoscopy would be very useful.

Speaker 5: I guess when you consider the story, it's also kind of reflective of how if you don't use it, you lose it. I feel like the fact that there were five presentations and no one decided to look at the eye, I feel like examining the eye, if you know how to do it and you're good at it, it's a relatively quick exam to do even if it is with a child.

When I've been on paediatrics, when you see kids come through emergency, when you look at their ears and nose, they're not going to ... ear, nose, throat ... they're not going to like it but you do it quickly and you have the parent to hold them down. I agree that we don't really have very good teaching as medical students, but perhaps it also reflects that when we go out into the field as doctors, whether it's GPs or otherwise, unless you use that skill a lot, you're not going to be good at using that skill. I feel like even though it's a quick exam that we can do, it's not done a lot systemically in ED or otherwise.

Speaker 6: I also think we don't practise it as part of our neurologic. We say, "Oh yes I'll do fundoscopy and look at everything, cranial nerve number one." Two, sorry. But we get used to saying that and then not actually doing that. Personally, I've never actually done very much fundoscopy, and certainly never in ED.

Facilitator: You were nodding to that?

Speaker 7: Yeah, I think I would agree with that point. I don't think I've ever seen even a senior doctor do fundoscopy on any patient. And I guess you kind of model what you see in the hospital. As a student, you learn the right way of doing it and taking your time and doing a full examination. But obviously, there's time constraints in hospital, and you just skip it. I think that's why maybe it was missed five times, because it's just not part of our routine practise.

Facilitator: It's an issue quite [inaudible 00:06:14]. Do you guys, when you do your EDs turns or other turns, do you see senior doctors doing fundoscopy?

Speaker: No.

Speaker 6: No. Slit lamp stuff is pretty much the only eye stuff I've seen.

Speaker 5: That's because they're looking for superficial trauma [crosstalk 00:06:28].

Facilitator: Okay. You were nodding in the back. To that.

Speaker 9: I see a [inaudible 00:06:35] in ED, but those [inaudible 00:06:40].

Facilitator: Right.

Speaker 3: And one of the other things I was going to say is even when we do get taught to do fundoscopy, when it's in a big group as well, you can do it and you're seeing the right reflex, or whatever it was. But you don't actually know what you're looking at and that makes it really difficult without having someone telling you, "This is what you're looking for," and in a big group, it makes it more difficult because there's not enough time often.

Facilitator: Do other people find that with learning it? Or with the way you're being taught fundoscopy?

Speaker 9: To be honest, I can't ever remember being formally taught fundoscopy, except as a theoretical, "Here's some images."

Speaker 5: [crosstalk 00:07:20] this is what you expect to see in this pathology or that pathology and then we get an exam question where they printed it out really nicely. So, we can do it from a textbook perspective but if someone asked me to do fundoscopy, I wouldn't even know if I'm doing it correctly.

Speaker 3: Like the technique involved.

Speaker 10: [crosstalk 00:07:40] fundoscopy, I actually believe I have no idea what I'm looking at. It's not like [inaudible 00:07:51] ask a doctor to show us how to do it. I think that's the culprit. Not having a learning process, I guess.

Facilitator: So, it's sort of the fact that if you [inaudible 00:08:07] someone else will listen and tell you what you should be finding, but if you do fundoscopy, that doesn't happen?

Speaker 3: Because it's only you who's seeing it. You're supposed to hear a murmur, that could put the stethoscope exactly where you're supposed to hear it. Whereas with fundoscopy, two people can't look through the [crosstalk 00:08:24] at the same time.

Speaker 11: Back in my university, what happens is that they're very fond of fundoscopies, even the senior doctors, they do that, but teaching the undergrads with fundoscopy is not so prevalent. Undergraduate is not actually expected to do fundoscopy there. But if you ask me a way to teach how to fundoscope a person, I guess it would be better to have a picture of the fundus beside, showing ... even if a senior consultant has seen once, he must take a picture of the fundus and then allow his students to see and tell them how to ... because it changes focuses along with the depth. You have to change the focus along with the depth as [inaudible 00:09:13] you need to change that part of the lens.

That was what I gained by experience, but I think that it would be better if some consultant would have told that to me earlier.

[crosstalk 00:09:28]

... doing that on a routine basis in other departments, it's not so prevalent.

Facilitator: Is that in your guys' university or medical school experience as well?

Speaker 12: Yeah, it's quite similar in here, in my university, usually, the end of grade, student is not expected to do the fundoscopy. When I was taking the exam in eye department, when I do the fundoscopy, the examiner is like, "Oh yeah, you do well." And just like that ... It's like I'm acting. I'm not doing it in a real situation.

Speaker 4: And even in OSCEs and stuff, for us, it's more just going through the moves when we get tested, if we're able to do it. Especially in second year when we have OSCEs. Because that's probably the last time we do. And even in your final years when you do long case and all of that, even if you have a neurological patient, the way our exams are structured is such that doing a fundoscopy as part of a neurological exam would probably be the little addendum at the end, being like, "I would have ideally, but I don't have enough time."

Speaker 6: It's said more than done.

Speaker 11: The only two people whom I have seen doing fundoscopies are the ophthalmologists and the neurosurgeons. I guess that application of fundoscopy is actually applicable everywhere, especially in medicine and paediatrics because these two are the areas where people can come with a lot of complications. A lot of problems. They could be then and there assessed and managed accordingly, but they have to wait for the consultation for the entire day, also. And then they get that and then they start managing that person.

If an undergraduate is actually trained to do fundoscopy, he would ... whatever he specialises in, he would be able to do and see that.

Facilitator: That kind of idea of picking up pathology early, I suppose that's one of the things we, in other focus groups like that, we picked that up as perhaps one of the motivators for why people might do fundoscopy. If you guys think of the other clinical skills you learned, you mentioned otoscopy or listening to heart sounds, are there particular motivators for you learning different clinical skills? Do you think what things drive you to spend more time practising one clinical skill over another?

Speaker 6: Second patient in GP, practising looking at their ears.

Facilitator: So, ears to do it, or?

Speaker 6: Ears and also commonality. If you're examining a child in GP, you're pretty much going to look at their ears.

Speaker 7: I think it's also exposure. If you're using those skills every day, you'd feel like it's relevant. You'd want to be good at it.

Speaker 4: The other thing is equipment. Using a stethoscope becomes second nature to us but using a fundoscope and just turning it on, if you haven't done it for a while, you get confused.

Facilitator: My OSCE, I shined the light into my own eye and I still passed.

Speaker 4: And that's the thing with ophthalmology. Generally, even when we do slit lamps and all of that stuff, it's fine when you're shown to do it. But if you were to come back a couple of weeks later to do it, I can't even figure out where the on button is half the time. Because it's quite complicated technology as opposed to just putting a steth in your ears.

Facilitator: Do you think that ... complicated technology point an interesting thing. Is there anything comparative examination skills you have to do that have complicated ... there are a lot of buttons even on the ophthalmoscope and different elements like that. What about an otoscope? It doesn't have a focusing dial but you still have to get the light and the setting right and that kind of thing.

Speaker 6: It's probably comparative. It's just because we use it more so it's less daunting.

Speaker 5: I think it's also willingness to be taught by clinicians, that affects whether we use an examination [crosstalk 00:13:36]. I think I've never really come across any clinician other than an ophthalmologist that would be even remotely willing to even talk about what is involved with fundoscopy and detecting the [inaudible 00:13:50], how to do it properly.

As someone mentioned before, we model our clinical learning according to what is modelled for us. I think, again, because it's not routinely done by senior clinicians, we don't do it either.

Speaker 7: I think one of the other things as well is if you compare something like a fundoscope compared to a steth, again, you're at a distance from the patient. When you're doing fundoscopy, you're really right up in their face and it takes some technique, the position of your hands and all of that stuff.

If you're nervous, it often comes across that you're not very confident and that sort of trips you up. Your concerned about being in someone's face for too long or putting the light in their eyes for too long because of the patient comfort. And if you haven't done it too many times, I guess you're less likely to be smooth in doing it. It's just plain irritating for them to have something in their eyes. With a student doing it again, and again, and again.

Facilitator: That's come up a couple of times when we've run these focus groups. Does that run through your head? Some of the ways other people have phrased that is saying they're doing an examination and they think, "Well, this is uncomfortable for the patient and I don't know how much I'm going to learn from this if I don't necessarily do it." Is that the kind of things you guys are thinking? Or are you thinking something else as you're picking the examinations you'll do [crosstalk 00:15:11]?

Speaker 7: The other thing as well is I've been aware of that, and I've just done it, I've got the right reflex and the doctor will be like, "Can you see this?" And I'm like, "Oh, yeah, yeah, yeah. I can." Just because you're mindful that you're taking time.

Speaker 1: And the doctor's going to have to go back and redo it anyway.

It's like doing an abdominal exam on someone who's got peritonism. The least amount of people that poke their tummy is probably better.

Facilitator: We compared to doing an abdominal exam on somebody else, you're still putting your hand ... It's not like we walk around putting our hands on random people all the time. Putting your hand on someone's abdomen is a bit uncomfortable. If you compare that learning experience of having to examine someone's abdomen, it's a bit uncomfortable for you and the patient, versus the fundoscope, is there similarities or differences there?

Speaker 7: Doing an abdominal examination becomes second nature I guess. Just because we do it all the time. It's not as invasive [crosstalk 00:16:13].

Speaker 6: ... the time to people who don't really mind.

Facilitator: You were nodding your head in the back, there? Just in general?

One of the controversial things that came up previously was several people said that they found doing a PR exam was less confrontational that doing a direct ophthalmoscopy exam.

Speaker: No, definitely.

Facilitator: No? Okay. Good.

[crosstalk 00:16:48]

If you were to put that kind of scale of what exams you feel are most comfortable versus what you feel least comfortable doing, or say, most confident or least confident doing. Let's do a confidence thing, we'll say most confident or least confident. What are the ones you feel most confident in doing?

Speaker 4: Cardio.

[crosstalk 00:17:10], abdominal.

Facilitator: And why is that?

Speaker 4: Because you do it on pretty much every patient that comes in.

Facilitator: And what ones do you feel least confident?

Speaker 6: I'd probably say PR because you don't do that as much as a student. And I guess fundoscopy, because I never do it.

Speaker: PV.

Speaker 6: Oh, yeah. Vaginal exam. Or pap smear.

Facilitator: If you pick the differences in those, so the PV, the PR, and the fundoscope exam-

Speaker 6: I think we've also been told the fundoscopy [crosstalk 00:17:45].

Facilitator: What is it about those that makes you less confident in doing them? Frequency?

Speaker 6: Frequency, yeah. You just don't have the skills in doing it. You feel you're going to cause discomfort for the patient.

Speaker 5: Obviously, socially, doing a PR or PV exam, it's quite confronting for the patient as well. I guess on top of that, whether it's indicated or not, some patients, I find, are quite happy to have that. If the clinician suggested it and they're like, "You have to learn somehow."

I think with fundoscopy, you don't really get that opportunity to look again. I think.

Facilitator: Sorry, that opportunity to look again?

Speaker 5: Oh, sorry. As in with fundoscopy, I feel like it's never really offered as students to us as another learning opportunity in addition to examining the patient. I think other doctors don't really view that as something that you need to learn how to do well. Whereas general surgeons will say, "You need to know and be confident about doing a PR exam because if you don't put your finger in it, you'll put your foot in it."

Whereas obs-gyn doctors will say ... you always ask about PV bleeding and if they're having any issues with that. And you always do a speculum exam if that's their presented complaint because ... Well, there isn't a quote for that one.

It's integral for them. I guess we haven't been taught that fundoscopy is integral in examination of the patient. But your story that you told us tells us otherwise.

Facilitator: In terms of those, I suppose coming back to the motivation and things, motivating people to do fundoscopy, what things do you think would change that so that it did feel that it was something you walk out being confident in doing?

Speaker 5: I guess being told stories [crosstalk 00:19:45] being told stories about how a simple fundoscopy can prevent so much complications to a patient and I think as medical students, we aren't taught the value of sight and how easily it can be lost. I can't speak for everyone's university life, the teaching around eye medicine is quite lacking and perhaps the undercurrent is unless you're going to specialise in that area, you don't really need to know it. That's the growing feel that I have.

Speaker 4: Because we all to obstetrics, we all do some sort of abdominal surgical term. But even PR and PV, we get the mantra of, "You don't put you're finger in it, you put your foot in it," drilled into us. But ophthalmology, we don't generally do rotations in. I know Sydney University does but we don't.

Facilitator: Where are you guys from?

Speaker 4: Western Sydney.

Speaker 3: I think one of the other things that I found useful from today's lecture was that how you mentioned, the eye signs, are really important in terms of systemic ... as an indicator of systemic cardiovascular risks and stuff. And if you explain that to students. I've never really heard that framed. If you think about high-yield targeted examinations, theoretically, doing fundoscopy would be really useful but we don't necessarily make the connection.

I think that if you were taught that, then you may use fundoscopy as routine in your cardiovascular examination. For example, you've got a diabetic hypertensive patient to monitor severity of their microvascular disease, I think that's really, really useful.

Facilitator: In terms of then doing the exam ... We've talked about how it's technically a difficult exam to do, if you take out [inaudible 00:21:47]. You take out the technical difficulty, do you think that would be more done? Say, otoscopy is an easier exam to do. Do you think that plays into why it's done more frequently, or? It's a bit of a vague question.

Speaker 1: I think the biggest barrier for us is we're not confident in skills and we don't know how to interpret the things yet. What you see. So yes, if you made it easier to access the retina and visualise that, it's going to be easy to interpret because you can see it quicker and easier.

Speaker 4: The other thing is availability of instruments. Often, in the ward, if we ever needed a ... not that I ever have, but I can imagine just running around everywhere looking for one. Whereas in an OSCE exam, everything is in front of you and you still don't use it. But it just triggers you to say, "I would use it."

The availability of the device itself is quite restricting. Even if you thought of using it, you could never find it unless you were in an optha clinic.

Facilitator: If you think of just in terms of timing of how often you do an exam and when you feel comfortable with an exam, when do you think was the turnaround point when you felt comfortable with things? You do cardiovascular term in year one or two and then you've rotated multiple times, when do you think you've started feeling comfortable with the exams that you do?

Speaker: Third year.

Facilitator: Third year for you guys?

Speaker 11: It was ... We have actually differences in the rotations there. It's quite difficult to compare the two. Should I tell you about that?

Facilitator: Yeah, tell me about how does it work. [crosstalk 00:23:32]

You're from India? Which part of India?

Speaker 11: Yes, I'm from India. Northern India, [inaudible 00:23:34].

What happens there is we have four and a half year course in which the first year is just theoretical, concentrating in anatomy and physiology and biochemistry. And in the others, we have the clinical rotations as well, starting from the second year onwards and we are posted at all the departments each year. Each year.

We have medicine rotation for three months in the second year, then two months in the third year, then again, three months in the fourth year. We have that sort of a thing. I guess after second year, we were quite confident enough to do that.

Facilitator: How many hours? Let's say, abdominal exam, how many abdominal exams did you have to do, as a ballpark, do you think, before you felt comfortable?

Speaker 11: I guess three or four times.

Facilitator: Three or four times? [crosstalk 00:24:27]-

Speaker 11: Once you find the split, you know how to look for it. Once you feel how it feels like, you know how to look for it.

Facilitator: That's a good point. Do you guys agree with that? Once you've found it three or four times, maybe not feel comfortable with doing things, but if you found [crosstalk 00:24:44]-

Speaker 11: You found three or four times, liver. You found three or four times a spleen, you're comfortable with that.

Speaker 1: It's true. Even if it's over 10 exams [crosstalk 00:24:53].

Facilitator: There's a bit of people looking a bit dissent here. What did you think of that?

Speaker 9: I guess it depends on how many times you can actually [crosstalk 00:25:05]-

Speaker 11: That's exactly the thing, you feel for that three to four times and you're fine. You find the fluid very well, I guess in two or three times? And you're comfortable with that.

Speaker 9: [crosstalk 00:25:13] but half of the times, [inaudible 00:25:17].

Facilitator: Right, 100 times and it was normal, you wouldn't necessarily feel comfortable. Is that what you're saying?

Speaker 9: Yeah.

Facilitator: If you're seeing the pathology a couple of times [crosstalk 00:25:29]-

Speaker 11: Yes, yes. That is what I meant.

Speaker 5: I guess another part is learning how to do exams on different patients, so being able to do an abdominal exam on a combative patient or being able to do an abdominal exam on a confused patient who's continuously trying to bat your arms away, or trying to do an exam in a ReCell setting where there's a lot going on around you but you're still able to focus on the task at hand.

I think the number plays a part but being able to take that skill and put it into a whole bunch of different scenarios and reproduce it at the same time. I think I was on my ED rotation last and I hadn't really done much clinical medicine in a while. When I was getting back into the swing of doing it in a whole bunch of different contexts, I think that's when I was feeling more confident because I was able to rely on myself to do it regardless of what the situation was.

Facilitator: Is there a difference than say ... They talk about the stages of learning and then maybe if you see it up to three or four times of finding something, you can say, "I know I can do that exam," to then feel like there's a level of confidence in your expert ... say, you're in her ED term and the [inaudible 00:26:42] is not going to reexamine you, you would feel comfortable in your examination? Is that another level again, or?

Speaker 3: I think it's different because if you have an abdominal examination, you're looking for masses, you're looking for skin colour changes, and tenderness. Just looking at even a normal fundoscopy exam, there are so many elements. And even when you pointed out on the screen all the different [inaudible 00:27:06] this and that, technically, it's really difficult to interpret.

Speaker 11: It's actually a direct ophthalmoscopy is technically confronting because you have to see the pathologies and you have to focus that in the same point.

As you trace a vessel, you need to change the focus along with it. And that's actually confronting.

Speaker 3: I don't think I've ever been confident enough to say if I saw something first, and then to report my findings and the consultant would check, maybe I could pick ... Especially because you don't see the whole field, I could pick maybe one. [inaudible 00:27:42] for example. Something like that, which is quite obvious. But then they'd say, "What about this, that, and that?" I was like, "Oh, well, I haven't really gotten to that side of the field in there first place."

Facilitator: And in terms of learning a new clinical skill, so if you pick one of the ones. Let's say, otoscopy. I jumped back to because it's quite similar kind of thing, how did you go about learning otoscopy? Do you read textbooks first and then go do it or do you think watching YouTube videos? [crosstalk 00:28:17]-

Speaker 4: From memory, the ENT was like, "Go to the ward and find 30 patients who are normal and then try and look for pathology." So, we just practised actually doing. I think that's the best way.

Facilitator: Yeah, good. Does other people have that experience in training? Do 30 normals and then come look for pathology?

Speaker 11: Exactly. It's always [inaudible 00:28:41].

[crosstalk 00:28:43]-

Facilitator: For you guys? Where abouts are you from, sorry?

Speaker 12: From Indonesia.

Facilitator: Was your training over there similar?

Speaker 12: Yeah, it's quite similar. Actually, we have many patients as an outpatient clinic, especially in the neurovascular room. We try to do the fundoscopy in that room and we also ... When I do the fundoscopy examination, the first thing that I look for is the [inaudible 00:29:25]? The optha ... the disc? And it's just about that. I didn't compare the normal disc ratio and the abnormal one.

I just look for the [inaudible 00:29:44] and the optic disc. And the macula.

Facilitator: But you could find those things, they did enough training in that kind of clinic setting that you felt like you could find those things?

Speaker 12: Some patient, I found it. And some patient, also I cannot find.

Speaker 13: For me, myself, I am not confident enough to interpret what is the result. [inaudible 00:30:14] model to study, I had an experience when I was in my first year ... first month of my first year as a medical student, we trained to measure the blood pressure and vital signs. We have a [inaudible 00:30:30] course, [inaudible 00:30:33] theory and then after that, we do the real thing when the people get to the .... In the weekend, they usually ... The people go to the park and we have a place that ... We are medical students, we offered you to check this [inaudible 00:30:55]. They know we are a medical student and we're still learning and each of the student have their own senior to be the supervisor.

So, we're sure and if we are not sure, we can ask her just by our side and we discuss. The people there also, they are happy with that. We are still learning, they understand it. When we come up with the result and this is the normal and in some cases, we found that there is a particular [inaudible 00:31:36] we should go to the doctor next time.

It can be useful for the fundoscopy that you can do that.

Facilitator: You have enough volume [crosstalk 00:31:46].

Speaker 6: We've had two models, ENT in particular. We had the Scorpio sessions, where there was five different rooms in the clinical school and a registrar in each of them and they went through different parts, so otitis media and this and that. That was one way. But there were about seven of us in each room and it was sort of like a mini lecture.

The other way we did it was in paediatrics and we had actual procedural sessions where there was three or four of us. Each of us were given an otoscope but in the beginning, there was an otoscope connected to a projector, and so the consultant got someone to stand up. He was like, "Can I have a look at your ear?" And then he showed us the procedure and it's all projected so we could see it all.

The second half, 10, 15 minutes, we practised on each other.

Facilitator: Do you find that ... The feedback component things, that's one of the other big things that's come up multiple times is that getting feedback on seeing something and then the consultant or someone saying, "This is what we should be able to show you," that's challenging with fundoscopy because you can't see what someone else is seeing.

And you're saying the ENT thing, that kind of watching someone else go through it live, makes it ... Is that a big component you're learning about in other clinical skills, you think?

Speaker: Definitely.

Facilitator: Having feedback?

Speaker 6: Anything with scopes that you can project it is really helpful because you can point at the screen and be like, "This is 'blah'."

Speaker 4: And you can also look at the person doing it to see what their technique is, what their angle is to get that view, especially when you're looking at ears and seeing the [inaudible 00:33:24] and all of that orientation and stuff like that. You learn which ear is which. If you're always just looking at the drum, for example.

Facilitator: The last question I would ask would be just about ... Are you guys final year at University of Sydney as well?

Speaker 6: I'm from Adelaide.

Facilitator: You're from Adelaide? So you guys are elective students over here? How come you got here?

Speaker 6: Snuck in. [crosstalk 00:33:53]-

Facilitator: And you guys are final year as well?

Are there any clinical skills ... Sounds like most people across the board are not quite confident in fundoscopy. Are there any other clinical skills like that where you think, "I'm at the tail-end of my medical degree, there's things I'm going to walk out not being super confident in the skills"?

I might just go around the room and everyone can name what ones they do or don't. What ones they don't feel ... Two that they feel very confident in and two that they [crosstalk 00:34:23]-

Speaker 12: I think ABG, I wouldn't confident in that because it's [inaudible 00:34:27] and you're so afraid of hurting the patient. The patient's comfort. And PV. But some of this is just lack of exposure. But otherwise, cardiovascular examination, oscitation, everything that would be almost second nature by now.

Speaker 4: I think for me, the most confident, doing an ECG, placing leads. Just getting a result in ED. Another one would just be cardio-resp exam. Two I'm not confident would be definitely PR exam because I haven't done any. We're supposed to do one in our surg term now. And probably PV.

Speaker 7: I would say also PV, I would not feel confident in. I feel like I'm growing. Otherwise, most of the systemic exams, I'm confident in.

Speaker 5: I agree, the cardio-resp and abdominal examinations are second nature because we've all done it since [inaudible 00:35:23]. I guess one that I'm less confident in might be a focused hand examination just because there's so many structures and so much anatomy there that I yet know almost nothing of. But it's an important area to be cognizant of.

Speaker 6: I definitely reckon a PV. Speculum exam, in particular. Just being able to see the cervix and all of that.

Speaker 9: Some weird stuff like hand exams, I've never really done before.

Speaker 3: I think I agree with everyone. PV. I'm pretty confident with the cardio-resp sort of stuff.

Facilitator: For you guys?

Speaker 12: I agree with all.

Facilitator: Same?

Speaker 11: I'm particularly not so good at hearing heart sounds. I just can't appreciate when you add an extra sound. Whether it's [inaudible 00:36:22], but I can't say [inaudible 00:36:28]. And ABG, also. It's difficult. We didn't actually have that much hands-on in ABG.

Speaker 10: I think for me, probably PV or [inaudible 00:36:43] exam. [inaudible 00:36:47] we don't actually get to see the [inaudible 00:36:51], really.

Speaker 9: My confident one's pretty much the same, cardiovascular and [inaudible 00:37:02] exams. The ones I am less confident in would be fundoscopy and otoscopy as well because I can't appreciate those signs, even though I've actually looked into them but the doctors just ... I can't just [inaudible 00:37:23].

Facilitator: Does anyone have any other particular things they want to add in there?

Speaker 7: We did a whole day at the Sydney Eye Hospital second year and that was really helpful when we went over the eye exam, how to do fundoscopy. And I actually felt quite confident after that but then I didn't really repeat the skill except for a couple of days in ophthalm that I had last term. Now I've forgotten.

Speaker 4: We have ophthalm notebooks and it doesn't really help us. Because you're literally just sitting there and it's really difficult teaching in a clinic when they've got so many patients to see in your one week that you're there and it's very arbitrary just getting signatures.

Honestly, just a workshop for a day or two or something-

Facilitator: I run the Scorpio at the North Shore Hospital and that model tends to run [crosstalk 00:38:25]-

Speaker 11: There's one [crosstalk 00:38:28] retinoscopy is also a bit daunting. At times, it's a bit daunting. At times you don't know the shadow is actually moving like this.

Facilitator: You guys have to do retinoscopy as a general medical student, yeah?

Speaker 11: No, not as a general medical student.

Facilitator: Alright.

Speaker 11: But yes, in ophthalmology, yes. We go. We do that, yeah.

Facilitator: Jeez, that's impressive as a medical student. Took me quite a while as an ophthalmologist to learn retinoscopy properly.

Speaker 11: That's sort of daunting.

Speaker 10: It takes a while.

Speaker 11: Especially the split one. I don't actually know where the split is. It's straight or it's curved. So even if it moves this way, it seems as if it's moving that way.

Facilitator: That's the end of the filming stuff then. We can organise a Scorpio here, is one of the things we were talking about doing. Do you think there would be interest for doing that if we did it? Alongside our colleagues?

Speaker 4: Probably after exams. [crosstalk 00:39:32]-

Facilitator: After exams? Yeah, not now.

Speaker 4: Print.

Facilitator: Print time?

Thanks very much, guys. Thank you for your contribution. You probably see-

How did we do?


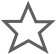

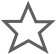

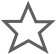

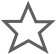

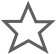


If you rate this transcript 3 or below, this agent will not work on your future orders
