# Supplementary material for: Optimising fundoscopy practices across the medical spectrum: A focus group study
Source: PLoS One. 2023 Jan 27;18(1):e0280937. doi: 10.1371/journal.pone.0280937 (PMC9882965; doi:10.1371/journal.pone.0280937)
Supplement: S1 Dataset — (ZIP) [file pone.0280937.s003.zip › minimal dataset/GP_2.docx]

## eFOCUS - General Practitioners_2

Facilitator 1: Is everyone happy to be involved in the focus group part of things? Any objection to us using [inaudible 00:00:08] has kindly agreed to help me with this project, so he's got far more expertise in running the focus group things, and he's also not got the ophthalmic hat on, kind of blindsides me to some of the things that otherwise people say that are quite interesting.

So I suppose having started that off, maybe we could just go around and I'll ask you your experience of using fundoscopy or stories you've had about it.

[crosstalk 00:00:51]

Speaker 1: I try and use it, and I don't think I use it very well. I think it'd be a lot better if I could dilate, without a doubt.

Facilitator 1: Mm-hmm (affirmative).

Speaker 1: Partly it's time, partly we just don't have the drops, I never thought of getting it. If someone would show [inaudible 00:01:15]

Speaker 2: Yeah, I think I pretend, if I ever do it. I'm trying to follow the vessels, and get to the disc. I think what you were saying before, if I'm looking for something it's probably something acute like papilledema. I don't find myself looking for [inaudible 00:01:35] changes.

Facilitator 1: Mm-hmm (affirmative).

Speaker 2: Partly because we're kind of trying to get other people to do that anyway, with the cycle of care.

Facilitator 1: Yeah.

Speaker 2: But I'm definitely not confident to do it. Probably the reason I would dilate someone's pupil is because if they're going to the ophthalmologist they're expecting to have to wait afterwards and they come to the doctor with a headache or whatever, and they think, "Oh, now I'm gonna dilate your pupils and you're gonna hang around forever and can't go pick up your kids or whatnot", then that's I think an inconvenience, which it's not. So the expectation ... yeah, trying to meet their expectations. Yeah, and it is that closeness, that physical closeness [inaudible 00:02:25] Am I gonna touch their nose with my nose?

Speaker 3: So I very rarely do it. I do do it sometimes, but very rarely, and there's load of reasons why not. One of them is time, and another is lack of knowledge, lack of expertise, I've lost that expertise. Another one is I can't really think of any situation in which it might change my management.

Facilitator 1: Mm-hmm (affirmative).

Speaker 3: So if I had someone with a history say of a detached retina, I might have a look out of interest sake, I probably wouldn't see anything but I would always send that patient to [inaudible 00:03:00] as an emergency anyway, regardless of what I saw. Then there's a dilating isSpeaker 3, so I had my eyes dilated just recently by an ophthalmologist, and they said it can last 12 hours before they get back to normal, and until I get to normal I shouldn't drive. So that kind of thing's not really very practical in general practise. Also, I think our patients would also drive, regardless of that. Then if they had a crash I'd feel terrible, and on it goes. We used to have the drops, I didn't have the drops, that just shows how often I dilate pupils.

Yeah, so for many reasons, there's so many barriers, and time. Yeah, and getting the room dark enough, and not knowing what I'm looking at. Particularly for papilledema, I guess if it's obvious, it's obvious, but the subtle changes. We see patients, they usually come here very early for stuff, they don't come here with florid pathology usually. So picking up early changes and subtle things would be hard. So for all those reasons.

Facilitator 1: Yeah.

Facilitator 2: [inaudible 00:04:16]

Speaker 1: Yeah.

Speaker 3: Yeah, we've got [inaudible 00:04:21] thousands of them.

Speaker 1: And you can get them in the same day.

Speaker 2: And I think the optometrists have good relationships with specialists too. So if someone goes [inaudible 00:04:30] go to the optometrist and they get sent on in. I think I do [inaudible 00:04:46]and corneal abrasions.

Facilitator 1: So you said it wouldn't normally change your management pattern?

Speaker 3: No.

Facilitator 1: Would you other guys agree with that?

Speaker 1: There's not much that would change.

Speaker 2: I think I've gone for the ... Kind of know what I'm going to do by then.

Speaker 3: So if you have somebody who you're suspecting say an inter corneal lesion, brain cancer or something like that, is having headaches and vomiting, you're thinking regardless of what you se on fundoscopy that patient is being sent off for an MRI or a CT scan. They're being sent urgently to a neurologist, and part of that is I think a privilege to work where we work.

Facilitator 1: Mm-hmm (affirmative).

Speaker 3: But it means that we get lazy, I guess. But it's also partially patient expectations, you know? They say, "I think I've got something terribly wrong with my eye. I've got to go see my doctor, then they refer me to a specialist."

Facilitator 1: Right.

Speaker 3: Yeah, and we do.

Facilitator 1: Do you think that the expectation of patients coming through is to see the specialist as well, is that what you see?

Speaker 1: I'd probably want that for myself too. A problem with my eyes, I wouldn't see myself. I'd see a specialist.

Speaker 3: Yeah. [inaudible 00:05:57] is that your expectation as a specialist? I've got something terribly wrong with my eye-

Speaker 2: [inaudible 00:06:00]

Speaker 3: Are you gonna do fundoscopy and have a look?

Speaker 2: No, I don't think so. Alright, okay.

Facilitator 1: [inaudible 00:06:04] maybe I'll make some comments at the end. Yeah, I'll make some comments at the end [inaudible 00:06:19]

Facilitator 2: So it's not my expertise at all, how many patients would you have thought in your practise would be ... It would be a reasonable thing to do [inaudible 00:06:38] fundoscopy?

Speaker 3: Many of them.

Facilitator 2: One a month?

Speaker 3: Oh, no. Much more than that.

Facilitator 2: Much more than that?

Speaker 3: Yeah, many-

Speaker 2: Hypertensive, diabetic-

Speaker 3: Anybody with hypertension, anybody with diabetes. Yeah, anybody with [inaudible 00:06:49] and stuff like that-

Speaker 2: Macular degeneration.

Speaker 3: Though they aren't managed usually by ophthemol ... They're always managed by ophthalmology if they've got glaucoma, but any type 2 diabetic or hypertensive patient it's a reasonable thing to do, yeah. Like the symptom.

Facilitator 2: So it's kind of like you've got a utility belt, and you've got your ophthalmoscope there and you don't pull it out very often. Is that because it's not ... What are the barriers to actually pulling it out and having a look?

Speaker 3: For all those reasons.

Facilitator 2: All those reasons, yeah.

Speaker 2: But also I think the way it's designed is because the way the whole, let's say diabetes, the care of diabetes is designed so that we are evolving. So that people are having their eyes checked, their kidneys checked, so looking for any small organ, small vessel disease. So that's meant to be happening annually, anyway. So it gets outsourced, I think.

Facilitator 2: Yeah. We do a couple of workshops each year with [inaudible 00:07:48] cancer, diabetes, arthritis, and invariably these specialists who will start off saying, "Oh, we can't get the GPs involved in this patient's care", invariably they come to a point where they say, "I wish the GPs were here at this meeting, cause they know this sort of stuff." So I wonder do you think you have, because of your knowledge of the patient over a long period, you actually have a more powerful position to get say a diabetic patient to take their retinopathy seriously and maybe change the way they look after their diabetes?

Speaker 2: I think [inaudible 00:08:40]

Facilitator 2: Yeah, that's what I want to know.

Speaker 2: No, it would depend. Like if you had really good relationship with the patient, I think they really do trust our opinion. But there are times when I just go, "Okay, off you go, because I know that if you hear it from a specialist you'll do it."

Facilitator 1: Okay.

Speaker 2: I don't know, one of the-

Speaker 3: But equally as much, sometimes I [inaudible 00:08:50] I'm coming to you to see what you think.

Speaker 2: Yeah.

Speaker 1: Yeah I get that too.

Speaker 3: We never go, "Well I actually think something different."

Speaker 1: Yeah. I think that with fundoscopy you don't have to send them to an ophthalmologist for their diabetes cycle of care if the GP is confident in that.

Speaker 2: Right, yeah.

Speaker 1: It's just that I tend to like you refer that on because I don't feel confident in doing it.

Speaker 2: Yeah. So we won't ... We'll stay not confident.

Speaker 1: But you don't have to [inaudible 00:09:27]

Speaker 2: Okay. Excuse me.

Speaker 1: As long as you do it within the certain-

Speaker 2: Time frame.

Speaker 1: A year.

Facilitator 2: I'm sorry, I don't know your name.

Speaker 1: It's Speaker 1.

Facilitator 2: Speaker 1. Oh you're Speaker 1, okay. That means you must be ...

Speaker 2: I'm the practise manager Speaker 2.

Speaker 1: Speaker 2.

Facilitator 2: Okay, Speaker 2. I didn't have you.

Facilitator 1: So I suppose two things to throw in, coming from the screening point of view with the fundoscopy. The two things we find that we can pick with just a random screening fundoscopy is bad glaucoma, cause the patients don't tend to pick it themselves until it's really, really bad, and most of those referrals will come randomly through optometrists when they've kind of gone to have their glasses checked and they come through. But occasionally we get a GP who's had a look, and then it's normally GP, optometrist, and then around to us is the normal cycle of things. The other thing is like what you're saying, the diabetic check. Sometimes people pick up the diabetes as the first time by screening from the back of the eye, and actually in terms of the damage from the hypertensive disease for example, the retinopathy that you can see at the vessels around the disc like you just saw on ... Not that you have any, but that level of viewing around it is actually your best predictor for your cardiovascular mortality, better than a lot of the other scales for things.

So the other kind of screening things that you can get from it. But interpretation of those things is quite a challenge. Are there particular ... Do you think if doing fundoscopy was easier or interpreting the results was easier, would that increase the likelihood that you would do it? Or knowing now that you can screen for those other things, would that increase the likelihood that you would do it?

Speaker 3: Yeah.

Speaker 2: Yeah I think so.

Speaker 3: Are there guidelines surrounding fundoscopy for people as a screening?

Facilitator 1: So they don't currently have it as a screening guideline for ... No there's, glaucoma screening guidelines recommend that once you hit the age of 35 to 40 that you have an optometry screening anyway, and in family history that's that young. When they're just general they suggest 50 as an optometry screening, but there's nothing currently recommending routine fundoscopy. For the diabetic checks you were talking about there is an item number for GPs to do a [inaudible 00:11:56] of the diabetics who aren't yet in the cycle of seeing a specialist.

Speaker 1: Yeah.

Facilitator 1: So it kind of gives a rebate for doing that, so you can get a [inaudible 00:12:05] camera, which will give you a bigger view. Kind of offset the cost of doing that.

Speaker 3: I reckon it probably ... just say for the diabetic, hypertensive person who has been a bit borderline for a long time, it might ... Being able to take a photo, show them those changes or explain to them those changes, that might ... Because you can actually see, it's the only place you can really see the blood vessels. It might actually persuade them to be treated.

Facilitator 1: Hmm. If you had that access do you think that would change your patients as well, Speaker 1?

Speaker 1: Maybe. I think the big thing for me is how confident I am in what I see. [inaudible 00:12:51] skill, I've gotta do it more often for me to do it. It's like skin checks, you know I used to do a lot of skin, and I got pretty confident doing it. Then when you don't do it for a while you lose that confidence [inaudible 00:13:03] So if I was really confident with the eyes, I would probably do it again, but I don't feel confident in doing it so I'd still send them off.

Facilitator 1: So we asked the same questions to medical students, and some of the things they were saying before was that the fact of being that close up to the patient, like you mentioned, is a bit of a barrier. Then difficulty interpreting it is a barrier. You guys already mentioned ... Would you agree they're two of the major barriers to doing things? Or they get in the way-

Speaker 2: I don't think it's that. For me getting up close to a patient isn't a barrier to doing it.

Facilitator 1: Right. [crosstalk 00:13:44]

Speaker 2: But I do notice it. I do notice it when I'm doing it, yeah. I notice it more than when I'm doing say a pap smear.

Facilitator 1: Right.

Speaker 3: Yeah, for sure.

Speaker 2: Cause you're really close, face-to-face, and you can often feel their breath. You often think, "Oh my God, I have this [inaudible 00:13:59] [crosstalk 00:14:02]

So I often will hold my breath when I'm doing it, like this.

Speaker 5: I mean I worked for an ophthalmologist and they used to just put a mask on.

Speaker 2: Yeah.

Speaker 5: And that was years ago.

Facilitator 1: I don't know what the practise pattern is here, but do you guys do pap smears as well? Is all the women's health with you guys?

Speaker 7: [inaudible 00:14:22]

Facilitator 1: If we compare you guys though, would pap smear versus direct opthtalmoscopy, [crosstalk 00:14:33] would you say that's ... Do you find that more confrontational, or the closeness of being to the patient, you're more aware of that with one or the other of those?

Speaker 7: About equally.

Speaker 2: What about breast check?

Speaker 7: No.

Speaker 5: But there's, hang on, then it works in the other cycle is that you have the male that comes in and doesn't want to see another male to have a-

Speaker 2: Prostate check.

Speaker 5: Prostate check. So it is really up to the individual patient, because we have the selection of both, they swap and change.

Facilitator 1: Right. So that's interesting, though, because the medical students [inaudible 00:15:10] but quite often, especially the junior ones were saying that closeness was definitely a barrier to them practising fundoscopy. But to you it doesn't feel like that's getting in the way particularly of doing it?

Speaker 3: No, cause I guess I'm quite used to putting up a clinical shield, yeah.

Speaker 5: But yeah, just while doing it, don't wanna lose my balance.

Speaker 3: So you've got your doctor brain on, but actually when you're doing it you don't actually notice-

Speaker 1: There's a lot of things that we do that we gotta get very close.

Speaker 2: Yeah.

Speaker 1: Even just listening to the heart, you've gotta ... I don't put the stethoscope that far away.

Speaker 2: Do you?

Speaker 3: But don't you find that fundoscopies, I think that's the most kind of-

Speaker 5: In your face.

Speaker 3: The closest, yeah.

Speaker 5: If you think about looking in someones ears it's probably the same, isn't it? Because it's [crosstalk 00:15:56] And if you're not confident, you're not gonna do it for ages.

Facilitator 2: In your training, [inaudible 00:16:12] using fundoscopy is not reinforced by their consultants, or their tutors in the hospitals, so that even in the diabetic clinics the students will tell us that their consultants say, "Oh, don't bother looking in the eye." They'll send them off to ophthalmology for that. So they got the impression that it's not a valued skill amongst the consultants. Is that something you remember back from your own training?

Speaker 2: Dementia's set in [crosstalk 00:16:37]

Speaker 3: You don't remember everything.

Facilitator 2: Oh, come on. It's not that far ago.

Speaker 3: It is! It's like 40 years ago.

Speaker 2: I can't remember being taught to use an ophthalmoscope, I can remember being taught how to [inaudible 00:16:47]

Facilitator 2: Okay.

Speaker 2: For my GP week. Like [inaudible 00:16:55] how to use an ophthalmoscope. Doesn't mean I wasn't ...

Speaker 5: That also says something about these endocrinologists, is that they maybe don't value it. They just say, you know how that's increasing increasing segregation of specialties. You know, we'll leave that for this person, and the kidneys for the renal people, and the feet for the podiatry people-

Speaker 2: And we've seen that. Yeah, because like often it's like the specialist won't go ... Like we have to be a bit more general, and we get a letter back saying, "Well they asked me that [inaudible 00:17:25] it's so specialised that, and that might reflect like a [inaudible 00:17:33]

Speaker 1: I wouldn't be surprised if the endocrinologist wasn't that confident [inaudible 00:17:36]

Speaker 2: Oh, absolutely. [crosstalk 00:17:38]

Speaker 1: They probably feel deskilled as well.

Facilitator 2: Yeah. [crosstalk 00:17:48]

Another question I wanted to ask you is one of my students [inaudible 00:17:53] GP placement up at [inaudible 00:18:01] and he said the GP there ran a boot camp three mornings a week down on the beach at 6 a.m., and he invited all his patients to come into the boot camp, and he said he gets about 20 a week turn out. I thought that's a pretty interesting commitment to help promotion. So the question is just thinking about the way you were talking about it then, are you aware of shifting from, "Okay, now I'm diagnosing, now I'm monitoring, now I'm screening"? Are you aware of shifting the way you're thinking about the patient in front of you at various times? And the question on the back of that is, so where does fundoscopy fit into that? If at all?

Speaker 3: Gee, that's a complicated question.

Facilitator 2: Yeah, sorry.

Speaker 1: I've just had a team down [crosstalk 00:18:46]

Speaker 3: How awesome though, that he does that.

Speaker 1: That's why I like it.

Speaker 3: Yeah, I'm not-

Speaker 2: You would do it.

Speaker 3: [inaudible 00:18:52] boot camp?

Speaker 2: I'm not like ... When someone comes in for their check up and there's not like a healthy check up, I'm very aware that I'm screening. Like thinking, "Okay, have you had [inaudible 00:19:07], pap smears, breast check" so I'm thinking-

Speaker 1: I know when you have the screens, like short screens, you don't even actually request fundoscopy.

Speaker 2: But I never think of doing fundoscopy. [crosstalk 00:19:21]

Speaker 3: Normal, abnormal, something like that.

Speaker 2: Yeah, maybe visual acuity?

Speaker 3: Yeah.

Facilitator 2: Mm-hmm (affirmative).

Speaker 1: You do the [inaudible 00:19:26]

Speaker 5: Yeah, I don't think I'd do fundoscopy, and I wouldn't think of it as a screening test, I must say.

Speaker 2: No.

Facilitator 2: Mm-hmm (affirmative). So when you would think of fundoscopy, what approach does that come up at?

Speaker 5: It would be more a diagnostic-

Facilitator 2: A diagnostic.

Speaker 5: Like for papilledema, that sort of thing, that's when I would generally have done it.

Facilitator 2: Okay, okay.

Speaker 5: But I would say the reason that I tend to not do it so much is that I find it hard without dilating a pupil. That's usually my reason for not doing it.

Facilitator 2: And do you recon the camera and those sorts of apps make it ... You get a better image with an undilated pupil?

Facilitator 1: Oh, definitely. The dilated pupils [inaudible 00:20:02] Well, sorry, that versus the normal. So these are easier with an undilated pupil, because you can see what you're doing and just rotate until you get the right spot, and then you've got a picture to look at afterwards.

Speaker 5: Yeah.

Facilitator 1: Otherwise you get this kind of-

Speaker 1: Well even then I thought the quality wasn't-

Facilitator 1: Yeah, it's not, it's a lot better when you go through a [crosstalk 00:20:23]

Speaker 2: The window, wouldn't it be more beneficial?

Facilitator 1: It is, and the darker the room the better. And there's one other one, so I think one of you guys ... Did you have a pen ophthalmoscope? Or one of your clinicians had a pen ophthalmoscope.

Speaker 2: Yeah, who was that?

Speaker 1: Oh, that was Davis.

Speaker 2: Davis. Yes. [crosstalk 00:20:38]

Facilitator 1: Yeah, and there's a clip-on to that that actually gives you a bit wider field of view again, so that's a bit easier.

So I suppose then, cause the thing we're hearing is different from the medical students, as their barriers were not feeling comfortable being that close to the patient. That's not really an isSpeaker 3 for you guys. The second thing was the techniques. A challenging technique to do, and that is a bit of an isSpeaker 3, especially with not practising it so often. Then the third thing there might be interpreting the results is a bit of a challenge once you've seen something.

I wonder, does [inaudible 00:21:18] come into it for you? As you were saying, you know, you don't feel confident about saying it's definitely right or wrong. Like there is or isn't some pathology there when you look at it. Are you aware of thinking about, if you're ruling something out or ruling something in when you have a look, does that ever cross your minds?

Speaker 5: Probably not as much as it should.

Speaker 3: Yes it would if I was doing that, but I'm not doing that.

Facilitator 1: Right.

Speaker 2: So if we were ruling something out or ruling something in based on using it as a tool to clarify some symptoms, and I was ruling something in or ruling something out, yes it would.

Speaker 3: But also, you want to do the right thing by your patient too. It's not just that.

Speaker 2: Yeah.

Speaker 5: More than anything.

Speaker 3: More than that, yes.

Speaker 2: I find it hard as a screener, picking up ... Yeah, if I wasn't confident with interpreting it saying, "No, everything's fine." If that was the only thing I was doing, like [inaudible 00:22:11] blood pressure as well, and checking all those other respective.

Facilitator 2: Mm-hmm (affirmative).

Speaker 2: I'd find that ... But I don't think I'd ever just use the one. That feels artificial, to just use a single thing. But I guess that's what often screening tools are.

Speaker 1: How do you mean artificial to one?

Speaker 2: Well, so if I'm thinking, "Okay, I'm gonna rule out hypertension in someone", I'd be looking for risks, like history, actually what is their blood pressure, the cardiac respectives. But if I was just go, "Okay, I'm just gonna look at your fundus and say you're fine without anything else ..." That feels artificial.

Speaker 1: Okay.

Speaker 3: It is artificial.

Speaker 2: But I guess that that's what out-screening does, and that's-

Speaker 3: [inaudible 00:23:00] Yeah.

Speaker 2: I mean breast check and mammogram is different I guess, but ...

Speaker 3: Yeah.

Facilitator 1: Yeah, I suppose so the ... Quite a few things from my perspective actually. So there are things I think from training our medical students who are often looking at kind of the emergency things, so it is the ruling in and out of papilledema is the thing we'd like people to be able to see. It would be challenging I suppose for you guys to have someone with a headache and then do the fundoscopy if you know that someone where you're particularly concerned you're gonna send them to emergency anyway, whether doing the fundoscopy would change the time frame for doing that.

I suppose the way we use it for that is there's some things like something called spontaneous venous pulsation, so when you look at the [inaudible 00:23:54] 90 percent of people, the veins will pulse with the normal, cause the CSF pressure gets transferred across to the optic nerve, and so you'll see the veins pulse. If you see that it's highly, highly sensitive that they have normal intracranial pressure. So that's the kind of thing that you could see with your fundoscope if you think someones got a headache that might be raised intracranial pressure, but if you see that then you can say, "Okay, this person has normal intracranial pressure, now they probably don't need to rush to the emergency department right now. They could get their outpatient MRI and come back to me in the next week and see things", to give one example.

I suppose the roundabout question I'm asking there is if we were to clarify with you guys what the, and where the doing a fundoscopy might change your management of a patient, would that be something that would increase the use of fundoscopy, do you think?

Speaker 5: Yeah.

Speaker 2: And then I think it's just about teaching an old dog new tricks again. Like just changing practise.

Facilitator 1: Mm-hmm (affirmative).

Speaker 2: You know actually this is important, I'm not used to it, I'm gonna have to get used to it. Which is probably a good skill to have if you're a doctor.

Facilitator 2: Students talked about their unwillingness to, pretty much what you said, their unwillingness to conduct an investigation, which they think they're incompetent at and they don't know how to interpret. Which is the problem they get all the way through their hospital training, and community training for that matter. You want them to actually say, "I've never done this before, I'm not competent", and the ones who don't say that you worry about. They thought that having a more robust way of measuring, like the camera might provide, would make them more confident to at least say, "I suspected there might be something that required fundoscopy, here's a photo. Send it to the ophthalmologist in the hospital, do you want to come down and see this patient?" As a kind of minimalist position would make them feel, "Okay, that's a skill I would like to have." Does that translate into general practise? The notion that being able to say, "I'm not skilled at this, but it might actually save a referral that's not appropriate, or relevant, or not timely." Yeah, I just wondered how you felt about that.

Speaker 5: I think it'd be nice to have that option, if you could do something like that and you thought, "Oh, actually I don't know if that's good or bad", if you could send it on to someone and get their opinion. But yes, that would ... I'd be cool, confident.

Speaker 2: Could be halfway, yeah.

Speaker 5: Yeah.

Speaker 3: Could you think of any other situation in which it might change our management in an acute situation apart from the venous pulsation?

Facilitator 1: So the ones we tend to do ... So the other big trial I'm running at the moment at Westmead and is screening patients who have hypertension, neurological symptoms, or vision loss when they come to emergency. There was a big trial in the US using a similar, like a more advanced camera than this, but takes a kind of 35 degree photo, so that you're getting five degrees ... This gives you 35 degrees. So you've got the whole maculate, you've got the blood vessels, you've got the disc in, a nice photo that you can see afterwards. They found that picked up pathology in about 14 percent of people who came with those kind of things, and fundoscopy was done in all of those patients but emergency physicians, and they picked up zero percent of that pathology-

Speaker 3: Wow.

Facilitator 1: Going through.

Speaker 3: In turning the pictures, looking at the pictures, they didn't pick it up?

Facilitator 1: No, no. When they looked at the pictures they picked it all up.

Speaker 3: Yeah.

Facilitator 1: When they tried to do the direct ophthalmoscopy-

Speaker 3: Oh, right, okay.

Facilitator 1: They picked up zero percent. So probably it's not ... The direct ophthalmoscopy is probably not that strong a technique as we'd like it to be.

Speaker 5: Did it show where it actually changed management?

Facilitator 1: Yeah. So in all of those, it was out of 350 patients, so roughly 50 of those patients it would have changed management. So it was things like they would need more rapid ... It's evidence of end-organ disease, so they had vascular changes up there, or they had papilledema so they needed more urgent coronal imaging and then [inaudible 00:28:18] or they had normal and no papilledema so you could deescalate their therapy.

Speaker 5: Okay.

Facilitator 1: And I suppose from a GP perspective it is that education thing, that if you can show them the photo and say, "Your hypertension is bad, here it's already doing this to your blood vessels, so that's what it's doing." I find that useful for patients I see, because I say, "Look, this is your blood vessels, this is what's happening in your brain, and your kidneys, and your heart. So we need to get things better"-

Speaker 3: And what do you do? Do you send them back to us?

Facilitator 1: I at least send them back to you. Some of the ones that have come back to you maybe with the diabetics and ... most of [crosstalk 00:28:51]

Sorry?

Speaker 5: That's cause you don't feel confident managing [inaudible 00:28:56] isn't that your job?

Facilitator 1: Yeah, it's the reverse. Also it's an interesting reverse, if I could [inaudible 00:29:07] doing the blood pressure management, compared to doing the fundoscopy.

Speaker 2: Okay if it's about the tool, and then you know we've got something better now than we used to have, like a doppler instead of a [inaudible 00:29:16] I don't know how to use a [inaudible 00:29:16] stethoscope, or whatever they're called on a pregnant woman. So why would I go back and do that if I could just get the doppler.

Facilitator 1: Yeah. The other advanced one they have, so the one we're using for the ED study is a little handheld camera that does the whole photo, about 45 degrees, in one go, and then it actually comes out with a computerised print-out saying they have diabetic retinopathy or not, and refer in this time frame. They cost about $6,000 at the moment.

Speaker 2: Is that the one that uses the images, matches images from-

Facilitator 1: Yeah.

Speaker 2: The cloud or something.

Facilitator 1: Yeah, oh that's these things. That's what our PhD students do. So he's building one of these, these are currently about $500, he's building one for a dollar at the moment.

Speaker 5: Wow.

Facilitator 1: And then it should match to the cloud and give you an answer on the basis of that. But the more expensive one gives you a far higher sensitivity for screening, so for example you could do that, and then it's giving you the answer to say, "Yes, I'm confident that my screening with this is giving you a reasonable standard of screening." So I suppose they're the things where we might see in the future, might change practise of things.

Speaker 2: Or even something the practise nurse could be doing.

Facilitator 1: Exactly, yeah, so the practise nurse could do [crosstalk 00:30:35]

Yeah, we've kind of answered most of the questions-

Facilitator 2: Well I just wanted to ask Speaker 1 if, from what I'm hearing, what would need to change for you ... Cause you said you don't do fundoscopy?

Speaker 1: No, I do it, but-

Facilitator 2: Rarely, yeah.

Speaker 1: Once or twice a year.

Speaker 2: It's pretty poor, really.

Facilitator 2: So I'm presuming the thing that would-

Speaker 1: Yeah, it really is [crosstalk 00:30:58]

Facilitator 2: That's what I wanted to ask, the biggest barrier is your competence?

Speaker 1: Yeah, probably is.

Facilitator 2: So if there was a way you could quickly get up to speed?

Speaker 1: So if I was using it for screening, [inaudible 00:31:09]

The only changes are [crosstalk 00:31:15]

Facilitator 2: So if we had ... Our approach to that is these things make it easier to get around the technical barriers to it, and other ones make it a bit easier again. So if that got around some of the technical aspects, and then we had an online training module, which we're building for free that says, "This is what you'll see, and this is ..." Yeah, actually CPD points, we have to give you CPD points for it, yep. And then if we were to build that, do you think that would ... So if that took out the technical aspect of doing it and the comfort level of analysing the screening of what you're doing, do you think you would then be more likely to ...

Speaker 1: Yeah, I actually didn't think that that made it any better than just-

Facilitator 2: Than what you would get in a direct, okay. Good. [crosstalk 00:32:05]

Speaker 1: Yeah. But then that could be because when I do it, I do it in the other room where it's really dark.

Speaker 3: Do you?

Speaker 2: Yeah. [crosstalk 00:32:09]

Speaker 1: I take them to a different room, so I do all of it there, cause once it's [inaudible 00:32:17] it's dark.

Speaker 3: Cause that's another intimacy you turn the lights off as well. Turn the lights off, yeah.

Facilitator 1: It does need this though, it needs a proper dark room to do it, yeah.

Speaker 3: I'm still not convinced about it changing management.

Facilitator 2: Okay.

Speaker 3: Enough for me to want to commit to doing a learning programme on it when there are competing priorities.

Facilitator 2: Sure.

Speaker 3: That's my concern.

Speaker 2: And I think I've got my head, got to move from diagnostic to screening I think.

Speaker 3: Yeah.

Speaker 1: Yeah, me too.

Speaker 5: Which is why we don't think to do it more often.

Speaker 2: If you felt confident in doing it, then you'd do it more?

Speaker 5: [inaudible 00:32:56] Do you mean, you're talking about screening diabetics? Or screening hypertensive patients?

Speaker 2: Just the average.

Speaker 5: Or screening-

Speaker 2: Just the general population even [inaudible 00:33:09]

Facilitator 1: So for me, I mean obviously I'm glaucoma oligoplastic, I'm a specialist. So glaucoma's my [inaudible 00:33:16] with these things. So I guess one way to phrase the question would be ... Glaucoma once you get over about 60 it's about 10 percent of the population. It's only about four to five percent of the population overall, but above 50, 10 percent of the population. Of which we only pick up about two to three percent, because everyone else is undiagnosed until they get this much field left over.

Speaker 2: Or if they've got a history, and then it's [inaudible 00:33:39]

Facilitator 1: The family history and it comes through, exactly. Yeah. But routine screen, so that is sensitive enough. I don't think that's good enough for picking up your diabetic maculopathy at this stage until it advances a couple of steps, but for picking up glaucoma with disc changes it's definitely sensitive enough for picking up that.

Speaker 2: I don't even ask about eyes when I'm doing screening [inaudible 00:34:01]

Facilitator 1: So I suppose the question I'm framing there is are there other things that are around, like something's 10 percent incidence in the population. Are there other things that you're screening, are there other things that are about 10 percent. I mean hypertension's probably a bit more-

Speaker 3: Yeah we screen for things [crosstalk 00:34:17]

Facilitator 1: That we screen for. Bowel cancer, prostate cancer, [inaudible 00:34:19]

Speaker 3: Yeah, we screen for things like certainly [inaudible 00:34:24]

Speaker 2: Cervical cancer's [crosstalk 00:34:26]

Speaker 3: And less than 10 percent [crosstalk 00:34:33]

Speaker 2: They're always underestimated, the window to the body.

Speaker 1: Yeah.

Speaker 3: Isn't there an isSpeaker 3 about dilating pupils if they have glaucoma?

Facilitator 1: So dilating them if they have glaucoma, the isSpeaker 3s, there's two things. If they've got a neurological thing, you lose access to assessing their pupils for the next eight hours or so. Tropicamide's the one you should probably have in clinic if you have one, cause it's got the shortest duration of action and it lasts for about eight hours total. So you've got to do all your pupil assessments before you put it in. If they've got a risk of angle closure, so that tends to be Southeast Asians, dark brown eyes, family history of angle closure, or anyone who's farsighted, so the glasses make their eyes look bigger, that means they're farsighted. It's the easy way to remember, if their eyes look smaller they're shortsighted. If their eyes look bigger, then they're farsighted.

Speaker 3: It's like coke bottles.

Facilitator 1: Exactly. So coke bottle glasses are the prime people for getting angle closure, because the eye is small so they need a big magnifier to make it work, and then everything's crowded. So those are the one's who are going to angle closure. Realistically, and I tell the med students this, although [inaudible 00:35:50] if you send someone into angle closure you've done them the best benefit you possibly could, because if you put the dilating drop in and they say, "Oh I've got really bad pain" and if you tell someone, "If you get pain you need to go straight to the hospital with it, then we can treat it acutely and they're gonna reverse out of it. But that means they were gonna go blind from it within the next year or two, and sit at home or go into the neurologists-

Speaker 3: Isn't it contraindication though? Is it-

Facilitator 1: So if they've got a history on there like that, then I would be ... They're the ones who I'd say no, it's fine, don't dilate, do what you can, refer on. If they don't have those high risk factors, then I think-

Speaker 2: And then consent to the procedure and known risk.

Facilitator 1: And they consent to it. And the only thing you warn them is it's difficult to drive for a couple of hours, and if you get eye pain in this eye you need to go straight to an eye specialist or the emergency department. But yeah, if you send them into angle closure, best thing you could've done, cause they would've sat at home with it for three days and then been blind, which is what the majority of my public patients end up with it at Westmead.

Speaker 3: Cause that is a barrier to me as well. The idea that-

Facilitator 1: In dilating you get an amazingly better view, with dilating.

Speaker 3: You do, yeah.

Facilitator 2: Can I just ask quick, we haven't asked before, it just occurred to me every once in a while you hear of a general physician, a general practitioner who's just a wiz at picking up one thing. You know, they get a reputation for being really good at spotting it. Do you have anything in your histories ... You know, in the past I've picked up something I'm very proud of, cause I could have easily missed it?

Speaker 5: I've had a subarachnoid haemorrhage-

Facilitator 2: Yep.

Speaker 5: And Addison's disease.

Facilitator 2: Okay.

Speaker 5: And those were two things.

Facilitator 2: Speaker 3?

Speaker 3: Well I picked up a Parkinson's disease very early, and I'm pretty good at things like genital herpes.

Speaker 2: Oh my God, Speaker 3! [crosstalk 00:37:50]

Facilitator 2: And so you're saying [inaudible 00:37:57] I'm interested in what actually gets you into a position of actually being a champion of picking up [crosstalk 00:38:03]

Facilitator 1: Have you got that on the website? [crosstalk 00:38:09]

Facilitator 2: Have you done something like that? Something you've been very proud of over the years?

Speaker 1: Temporary arthritis I picked up, acoustic neuroma ...

Speaker 5: We love these things, don't we though? It makes you [inaudible 00:38:23] when you pick up the poor patients.

Speaker 2: You feel like you've actually saved, like altered the course of their lives.

Speaker 1: Yeah.

Speaker 5: Yeah.

Facilitator 2: Yeah, what I'm just interested in is, is it a particular system that fascinates you, that takes you there?

Speaker 1: No.

Facilitator 2: And if the ophthalmologist wanted to actually just upskill people in screening, or in fundoscopy, what would be the things that are missing? Because presumably there's some GP out there who's just a wiz at picking up glaucoma, and know when all the signs say I better have a look at the back of the eye.

Speaker 1: Well I know of a GP who over at [inaudible 00:39:00] who does glaucoma testing. Just shoots some air in, he does it.

Facilitator 1: Yeah, [inaudible 00:39:09] pressure, yeah.

Speaker 1: I think there are a few GPs who would be doing that.

Speaker 2: I think there was a personal experience of them, like someone who's got a close history with something. So if my dad or mom had glaucoma or something, I'd probably be-

Facilitator 2: Be a little more alert to that, yeah. What about you Speaker 1, anything you're very proud of, you're good at picking up? You've picked up in the past?

Speaker 1: Melanomas.

Facilitator 2: So that's a screening thing.

Speaker 1: That's probably screening.

Facilitator 2: So that's screening, this is diagnosis. Yours is diagnosis.

Speaker 3: I've picked up a melanoma on-

Facilitator 2: Yours is frequencies.

Speaker 3: omebody's inside thigh, during a pap smear. Sort of, what's there? "Oh it's nothing, oh it's just a mole", she said, and it was a melanoma in situ.

Facilitator 2: Speaker 2?

Speaker 2: I'm trying to think. I've picked up an endometrial cancer. [inaudible 00:40:08] stuff more than anything, main cause it's-

Speaker 3: Cause you see a lot of it, yeah.

Speaker 2: But yeah, it's probably a [inaudible 00:40:14]

Facilitator 2: Yeah, yeah. [inaudible 00:40:20]

Facilitator 1: I think that answers my-

Facilitator 2: I think so, yeah.

Facilitator 1: This was very helpful, thank you, cause the medical student stuff we generated all those things, and then it's really useful. Cause we spoke to some second year medical students who haven't done any ophthalmology yet. We've spoken to some fourth years who are about to get out and they're very nervy about getting out, and then hearing from you guys is just really interesting to tie some of the things that we thought were trajectories from the second and fourth year medical students on. It's really clarified a lot of that information for how we should teach them, I thank you very much. Do you guys have other things you wanna ask me about ophthal-
