# Supplementary material for: Optimising fundoscopy practices across the medical spectrum: A focus group study
Source: PLoS One. 2023 Jan 27;18(1):e0280937. doi: 10.1371/journal.pone.0280937 (PMC9882965; doi:10.1371/journal.pone.0280937)
Supplement: S1 Dataset — (ZIP) [file pone.0280937.s003.zip › minimal dataset/Physicians.docx]

**eFOCUS Physicians**

Speaker 1: We analysed that as is ... do they get a correct response versus incorrect response, but I didn't take account of the fact that there was a 50 percent non-response rate for the direct ophthalmoscope and they just couldn't see anything, so they didn't answer any questions. Whereas there was about a 15 percent non-response rate for the [inaudible 00:00:17]. So if you take that as a screening test, it should be analysed as either correct response versus incorrect or no response, which is what the statistician told me. So we're rerunning the stats. It should be better than that. And also that was with second and fourth-year med students who weren't used to answering things, so I think that we're about to rerun it with position trainings next year, is the plan, so we should get some more interesting data.

Speaker 2: Probably won't be any better than this.

Speaker 3: I don't know that it will be that much better, because I don't know that we get that much more training between medical student days and... Even if you happen to have done an ophthalm term, it's usually a really small ... I did it as a resident and we did very little exposure.

Speaker 1: Yeah.

Speaker 3: And then you don't do it at all afterwards. I'm neuro and ophthalm, and I still feel like I can't. I wouldn't have been able to do very much at the finishing, or even now.

Speaker 5: I think the main thing that is shows it's one thing to recognise, but in the actual exam they can't take their phones in. [crosstalk 00:01:14]

Speaker 3: Yes, but I think it's in real world, like in ... So I guess that ... I mean, the idea is that we diagnose things earlier than, awe we've gotta go and get you to see the super busy eye clinic, and if you've got a photo that you take in on the ward that shows haemorrhages, then you send down to the eye reg, hopefully they'll come and see them straight away. I guess it's that. I still worry about whether people can that still need a bit more training to recognise the abnormalities.

Speaker 1: So the other [inaudible 00:01:46] is we're building an aligned training course in interpreting it. So there's ... we built it for interpreting the disc, but we're expanding it to interpreting the rest of the fundus. So that will come. It's pretty, like, within a year I'll have that ready and tested, and ACI's supporting it, and Atlantic Eyes are supporting it, so we should have a well built thing for doing that. But I think part of it would be I think ... I'll tell you the things that the med students said and I'll be interested in your feedback as to how you find it in training.

So, some of the things they said were that they were initially interested in direct ophthalmoscopy but they find they get no feedback when they do it. So, compared to say, they said if they're listening to a heart they know that their consult with the registrar is gonna listen and then tell them, or they can look in over their shoulder and say, no, you need to put your stethoscope here or no, you should be listening with the bell for this. But the direct ophthalmoscope they kind of feel like they go and have a look somewhere and no one can tell them whether they're right or not.

Speaker 5: Because we can't see what they're seeing.

Speaker 3: Yeah, yeah, yeah. That's right. But also, so I do the medical student teaching and we liaise with the ophthalm and you've heard from them here as well. They do them in second year 'cause they start their log book in second year. And really in second year, it's the start of when they're doing neuro so they're still reasonably junior and I think they're still just going through the motions when they do exams. They're never actually trying to think about looking for signs or abnormalities. They're just going through the motions and stuff. So they're supposed to do it through second year and third year. And third year they are meant to be looking for signs or abnormalities but they tend not to do that. And I think they're very passive when they do go to the eye hospital or come to eye clinic. And therefore as a result they don't really know how to pick abnormalities. There is the Scorpios at the ophthalm department does run for them, but it's like a one-off. Once in second year, that's it. And it's very right at the beginning. I mean, it's good that you show them this is the equipment, this is how you use it, but then ... We were trying to run another one in third year but timing wise I think it's going to be a bit difficult to keep running those. Yeah, they're ticking a list off the page and essentially that's all they do.

Speaker 2: Is there a teaching ophthalmoscope at the eye hospital? Because that's how I learned a lot of fundus was sitting with another ophthalmologist and they look through the lamp and then you can look through and see exactly what they're seeing and they can tell you ... Which is a bit like what it's gonna be having photos, I think. But, do they have that? Do they get exposed to that?

Speaker 1: They have that but not a lot. They'll have some photos that they'll look at that generally say the problem is that they'll very briefly have a look [crosstalk 00:04:20].

Speaker 2: And photos are nowhere near the same. [crosstalk 00:04:20] Like in [inaudible 00:04:21] at the moment compared to trying to assess a patient in real life.

Speaker 1: Yes. How do you think it is with physicians? So, the physicians going through ... As consult physicians, in a ward round thing, how often do you think you would use [inaudible 00:04:35] fundus?

Speaker 4: Zero.

Speaker 2: I do it every time but that's different.

Speaker 4: But in the office, rather than in the ward, I used to try and do it and I was able to do it using a direct ophthalmoscope. But in more recent years I've stopped doing it because I realised that I wasn't actually doing it very well. And without dilating the eyes I had a very limited view. When training in the hypertension clinic, we always had to do fundoscopy and so I think that's where I learned to do it. And recognise the sort of thing ... [crosstalk 00:05:17].

Speaker 3: I agree because I think the patients then go, "Oh cool, they've had a look at my eye.". I do diabetes clinic and it's actually more important to get a really proper view of the peripheries, whereas you really can't do that in an undilated eye. So if I've seen them they'll go, "Oh cool, she's had a look at that and that should be fine.". Whereas they really should have a proper wide view photograph to look at that and say I think then ... So mainly I do it if they've got sudden onset really bad decrease visual cue to make sure I'm not missing some massive haemorrhage or something obvious that they now have to call someone about then and there.

Speaker 1: But as a screening test you would ... [crosstalk 00:05:51].

Speaker 3: But it's not often [crosstalk 00:05:53]. Because I think that we really do ... I do a really poor job in the clinic. There's ophthalmoscopes everywhere but then I think it's just the outcome you get from it and the false reassurance for the patients in the screening clinic patient. And in the diabetes clinic we've got the option of sending them to have eye photographs down the track so I try to do that or get them to go and see the eye specialist, optometrist, etc. That's what we tend to do. But then it's really not great either because of the fact that they often don't go and do that. Optometry views I think are quite limited. And we get limited feedback on them about what they screen for. So that's where I try to ...

Speaker 4: I went to the optometrist only the other day. And he got ...

Speaker 2: The retinal scan?

Speaker 4: Yes. He had the most extraordinary photographs to show me after quite a brief examination. And they were fantastic. Un-dilated. Right to the periphery.

Speaker 3: We were talking about this. We were trying to get a new camera in for the diabetes clinic. At the moment the one we use has to be dilated and so that's very restricted. People don't want to use it because they can't drive, [inaudible 00:07:06], they've got to have someone to go home, da da da da da. But we wanna try to get funding for a new one that's a non-mydriatic fundus camera that has a reasonable view and it will be a lot quicker, self-focus and all that. Easy for the operator to do it.

Speaker 4: So my guy was using the $30,000 one.

Speaker 1: Yes.

Speaker 2: Maybe, yes.

Speaker 1: Or even more they had the Optos which is the [crosstalk 00:07:26] look a bit green so that ...

Speaker 4: Well the frames are expensive enough to help [crosstalk 00:07:31].

Speaker 3: [inaudible 00:07:33] about trying to combine and do something together. The one that she wants the Optos and I want ... It's very expensive. 'Cause we're looking at something like $30,000-$40,000 camera. And that one is $150,000-$160,000.

Speaker 1: And the Optos is actually not yet well clinically proven. [crosstalk 00:07:53]. The value of the pathology is closer in to the posterior pole.

Speaker 5: I think for us it's also related to the conditions that we see. So for us, I don't usually send people to the ophthalm clinic because of the vasculitis, the GCA's. I mean, it would be great. Again we probably need more equipment and list all that purpose which is a difficult task.

Speaker 2: Probably the main role in getting physicians good at it is not for the screening and the non-urgent stuff. It's gonna be in particular the emergency setting [crosstalk 00:08:25]. When you've got the acute headache and is it IOH or is it just a migraine and/or is it malignant hypertension or just high blood pressure. Those kinds of things.

Speaker 5: Yeah. The more general stuff. Yes, definitely but when you get to the special stuff ...

Speaker 1: So there's a lot of screening but in future there's some other applications that could come into it. I'll just tell you some of the things that have come up [inaudible 00:08:46]. So some of the things are things like, so the hypertensive changes that you were talking about are actually a stronger predictor of cardiovascular mortality than any of the other. So the actual blood pressure, their actual cholesterol levels. If you look at their arteriovenous calibre and the AV nicking and things, those are actually a stronger predictor of cardiovascular mortality that should drive your [crosstalk 00:09:07].

Speaker 4: We used to say nipping. You said nicking.

Speaker 1: Yeah, same thing. Right, yeah.

Speaker 4: We say AV nipping.

Speaker 3: I think if you can get a reliable image and a reasonable online training of some sort that you can keep accrediting and refreshing. Because I wouldn't see enough of it to really pick something more subtle. And then there's a wide application. And because you can then, I think the advantage is you can take easy videos and photo to then talking to someone about it and then gain experience that way. Exactly like Kate says, you've gotta train with someone. You can't just sort of see and have a look and you're not sure what you're seeing.

Speaker 1: So it ended up being a dual thing where to start with they'll be on one training how you interpret the photos but at the same time you effectively Watson will pick it up and you'll take a photo and it'll come back to you like an ECG does with a guideline. And the ECG spit out at the top is not always correct but it gives you the guide of what to do. So that's probably where it's headed.

Speaker 5: I think if you do want to take this out in the future to actual clinical cardiologists and things you actually need some form of accreditation [crosstalk 00:10:17]. That will be the main, I think.

Speaker 2: Even if they can just take the picture, that would be a good start. [crosstalk 00:10:21].

Speaker 3: Yes, that's already better than [crosstalk 00:10:22].

Speaker 5: [crosstalk 00:10:22] to start with but if they want to interpret a thing in the rooms, like in their private room they go, Oh, this is a blood pressure issue. They need to be trained and have some form of accreditation.

Speaker 2: Once it gets easier, because I've got the feeling that from when you trained Rubric compared to when we trained that it's actually gone downhill in how much people are using [crosstalk 00:10:43].

Speaker 5: Yes, I'm sure it is actually.

Speaker 2: [crosstalk 00:10:44] You say that as a general physician you could use a direct ophthalmoscope. I bet you the general physicians training these days will have no idea how to actually interpret anything. So if we make it easier I wonder if people will start to get a lot more interested in it again. And even the cardiologists who are very surgical. Given that you know that looking at retina can be helpful in assessing [crosstalk 00:11:04].

Speaker 1: Well this is the only time you've got in vivo view of the vessels [crosstalk 00:11:07] and the brain. [crosstalk 00:11:08]. So you're looking at the brain ...

Speaker 2: But there's no medicare item number, is there?

Speaker 1: Currently no.[crosstalk 00:11:18].

Speaker 5: We can't make it attractive by having a medicare item.

Speaker 1: If you do it outside of [crosstalk 00:11:16] there's a tele-medicine phone number. Oh sorry, the type 2 diabetics with a vision better than 6/12, if they're seen by a non-ophthalmologist, they have a medicare rebate with a non-redriatic camera.

Speaker 2: So that's the one you [crosstalk 00:11:33].

Speaker 5: How much is it?

Speaker 1: I think it's $50 or $100.

Speaker 2: And it's reasonable and it was meant to ...

Speaker 1: Enough that if the department did [crosstalk 00:11:42] the volume that they see it would pay for itself.

Speaker 3: Yes. So that was part of the reason why [crosstalk 00:11:45] to get [crosstalk 00:11:46]. Because at the moment how many are we doing? Like, seven? Or eight? And that's crap because it's no one wants to do it. [crosstalk 00:11:51] But if we get a better camera, they can do heaps a day and then it's easy [crosstalk 00:11:58] improved partials. So that's basically as easy as an OCT, is that right?

Speaker 1: Yeah. The $30,000 ones can be auto focus [inaudible 00:12:06] so like in one of our rooms where I'm at, we have one where a non-trained secretary comes in. All you do is you point it at the eye and the machine goes zooming, Fido and an ICT. Swaps to the other eye automatically and takes [crosstalk 00:12:21] ICT. So they're just sitting in front and you can have an admin person pressing the button.

Speaker 2: Our educators do it. We don't do it. But she's actually pretty good. She's been doing them for ages [crosstalk 00:12:35], but yeah, yeah, yeah, exactly.

Speaker 3: But they're probably more trained than the average admin person. Still, yeah.

Speaker 1: Two of the things you said there were interesting. One of the ones was, sorry ... Back to the other one which is that you were saying that you thought there'd be a false reassurance for patients [crosstalk 00:12:52] coming through. Can I give you a case example which is why I actually got interested in this whole thing and I'll just get your feedback from it. When I was at the eye hospital we had an eight year old girl come through who was sent with a referral from her psychiatrist saying please confirm functional vision loss in this girl and she was hand movements in one eye, 6/60 in the other eye. And the story was four months prior she'd presented to her GP with a bit of a headache and into St George emergency with headache and some blurry vision. She had a full neurological exam which was normal.

In the history she'd had an uncle and a cousin who died in short succession before she presented and they said maybe this is some kind of conversion syndrome and she was sent home. Then she had a worsening of the headache, saw the GP again with another neurological exam, which was reported normal and then back to the same emergency department with the same headache, bit more blurry vision, which was she was still 6/6 in each eye at the time but complained of blur. Some tingling in her fingers and neurological exam documented there was again reported normal but no fundoscopy. So she went home.[crosstalk 00:13:57].

She was then waiting outside her school and a girl, a kindergarten girl stepped onto the right in front of the bus and she reefed this girl off the road and saved the girl's life but put her shoulder out in doing so. And so presented to a different emergency that night with pain in the shoulder, tingling in the fingers, some worsening blurry vision and a worse headache. And they had a neurological exam again, no fundoscopy, and still 6/6 vision. That even recorded visual acuity and it was referred to the psych team with a conversion syndrome because she's feeling very unwell because this is a near death experience and association with other stuff. She then had one more A and E presentation when the visual acuity did go down and she went straight through to the psych team, didn't have a neuro exam at that point. [crosstalk 00:14:42]. And then the psych team referred to us to say confirm visual loss. Functional vision loss. And she had the worst papilledema I've ever seen in my life. And when they finally did her lumbar puncture over at the kids hospital, her opening pressure was 80.

Speaker 2: Oh my God.

Speaker 1: And so she had permanent vision loss in both eyes.

Speaker 2: Oh, that's awful.

Speaker 1: But there was actually six successive neurological exams conducted there and none of them had a fundoscopy.

Speaker 5: [inaudible 00:15:09] always complain of blurry vision.

Speaker 3: Would they have ... especially in peds [crosstalk 00:15:14].

Speaker 1: Yeah, it's an eight year old so I think a lot of people [crosstalk 00:15:16].

Speaker 4: Thank [crosstalk 00:15:16] psychiatrist, that's something ...

Speaker 5: Isn't it easier to [crosstalk 00:15:20]

Speaker 2: Two year olds are hard but eight year olds will sit down for a ...

Speaker 3: That's not an excuse anyway, it's just when that's the primary complaint. And did a paediatrician actually see during this?

Speaker 1: It was ED so peds did see her.

Speaker 2: Even if anyone had bothered to just get one of these pictures. At least someone would have [crosstalk 00:15:42]. Yeah.

Speaker 4: What was the diagnosis?

Speaker 2: Not sure.

Speaker 4: So, what was the diagnosis?

Speaker 1: It was [inaudible 00:15:48]. So that's slightly out of the way [inaudible 00:15:51].

Speaker 5: That's very unfortunate.

Speaker 2: Did she get any of her vision back?

Speaker 1: She got some vision back but she's still 6/60 in one eye and 6/18 in the other so [crosstalk 00:16:00].

Speaker 2: Are they suing?

Speaker 1: I don't have the follow up notes from Royal Kids to know.

Speaker 2: It's actually justified in that case.

Speaker 1: Very well justified. What's your feedback on that process of the case? Because the interesting thing ... So when I said this to ED, I've done focus groups with three separate ED groups, consultant physician groups, they said they all would probably have skipped over the fundoscopy in their examination. [crosstalk 00:16:27]. So it's that they wouldn't be confident in the answer of what their getting. I said if they came with a headache, the incident of blurry vision would make them concerned but they said the visual acuity's 6/6, they came with a headache. In their headache routine they said no they probably wouldn't do a fundoscopy in this [inaudible 00:16:48].

Speaker 5: Look, I can say the neurological consult even done in ED is pretty crap half the time. Adding on fundoscopy ...

Speaker 2: I can't speak because I don't do ED. [crosstalk 00:17:01]. If you're a physician trainee you would say alright I'd do everything but whether they would pick something is a different matter but they should go and a physician. Like if there was a med reg seeing this person, or a peds reg. They should really at least have a try to look at a thing. And if they can't, I think they really should ask a physician and/or ask someone to come and see them that might give them a little [inaudible 00:17:29]. Because it is a major differential. And it's also the fact that they thought oh, it's psych to begin with when they first thought that they went, "Oh no, it's crap. Let's not bother.".

Speaker 5: A lot of places don't have an ED medical registrar.

Speaker 2: Yeah, exactly. I can't comment on ED. I can't comment on A and E. But all I can comment on if they come through to our ...

Speaker 5: It could be an ED resident doing a neurological exam going, "That's fine, go home". Unless [crosstalk 00:17:49].They normally get a proper neuro reg review.

Speaker 2: But I think ED's being negligent. I think that in a headache situation you must look at the fundus. And there is no excuse. And if you can't do it then you find someone that can do it. [crosstalk 00:18:03].

Speaker 1: Just as physicians, if you had, like on the ward if you had someone with a headache, do you think the fundus gets examined?

Speaker 5: No. We look at GCA's, we don't see a lot of fundus.

Speaker 2: Really?

Speaker 5: Well, yes.

Speaker 2: No, I don't mean that in a creepy way.

Speaker 5: Don't look at me like that. It's the truth.

Speaker 1: Everything's de-identified so that nothing [crosstalk 00:18:21].

Speaker 5: No one looks at it. We just go based on helix. We don't have ophthalmoscope with us. We don't dilate the eyes. I mean, [inaudible 00:18:29].

Speaker 6: See I don't know if I was on the ward [crosstalk 00:18:29].

Speaker 5: There is but we're just gonna go ... [crosstalk 00:18:36].

Speaker 2: But it's missing most of the time and the problem is with that direct ophthalmoscope no one can see anything. [crosstalk 00:18:41]

Speaker 6: Like in your example, even if there was [inaudible 00:18:43] and one of our registrar's went to see them. So, six ED presentations. Even if she had early papilledema people may not recognise it, won't be willing to call it. People will still ... You want to get an earlier diagnosis until the disc is really blurry and someone goes, "Yeah, I really can't see like this much now.". Now I'm really worried. So, but the skills and the technique, like the recognition is still not gonna be there. Even if someone mentions [crosstalk 00:19:11].

Speaker 2: But I think it can be trained on. Like, for example, in ED there mostly would be a slip arm and you can't actually get a reasonable view of the slip arm or the back of the eye. I recall when I was doing training and stuff.

Speaker 6: My new special lens with the [crosstalk 00:19:27].

Speaker 2: Yeah, yeah, yeah. When I was doing it in the eye clinic I went, "Oh cool, you can just sit here and do it and potentially that could be done in ED but people wouldn't have the skill to do that.

Speaker 1: Yeah, that's quite a challenge.

Speaker 7: Which is where that kind of thing is gonna be there all the time.

Speaker 2: So I think it would help a little bit in that circumstance to, at least in the short term, take a picture and send it to someone who ... I could be concerned I wanna ... 'Cause then you just cut out, if the eye reg has to come in, they have to have a special req. They can't be there in ED the whole time. As the state of tomorrow how can we change clinical practise is that we take a photo that they can see all urgent cases and it will pick up abnormality in 10% at least if we play it that.

Speaker 5: I mean, it will become [inaudible 00:20:10]. The dermatology way now days is take a photo of the rash, send it to the derm reg ...

Speaker 3: Well that's what surgeons do. "Can you take a picture of the CT Scan and show me. Oh yeah, no, that's fine.". No but this is done a lot [crosstalk 00:20:20]. At least you're picking up something like that. That's like a big bad [crosstalk 00:20:25].

Speaker 4: As long as it doesn't become a misuse of technology. I suppose that the end result of this will be nobody will be actually trying to look at fundi. They'll be taking photographs and sending them off and you guys [crosstalk 00:20:42].

Speaker 7: But they are looking at it, aren't they? They still have to find it, they have to enlarge it and send it off to their boss so ...

Speaker 3: But then, they are looking at it. Because they're not looking at it now.

Speaker 4: Well as long as it becomes a routine part of neurological assessments.

Speaker 7: But that's what we need to bring back, isn't it? Just like doing the power and the sensation you do the fundal [crosstalk 00:20:59]. Which is what it should be really.

Speaker 5: Something may be starting off in ED and saying hey, make sure that all patients who come in with a headache, using that, they own ED specific phone and camera. And get them to take a photo. Maybe link it up with the eye reg. As a start of something, link it up with the eye reg to go hey, does this patient need to come upstairs or not from ED? [crosstalk 00:21:24] No, it's fine.

Speaker 1: We're actually just starting that next week ...

Speaker 2: Oh, you are?

Speaker 1: [crosstalk 00:21:27] with that camera. So that's a portable non-redriatic camera. That's the hand-held one that's $6000. And it gives you a 45 degree view so instead of the 25 degree, it's everything in the one photo. So everyone comes in with those things and it gets linked to Power Chat on Medilease. So when you look at the patient you'll have their BP and then you'll have a photo of the fundus with it.

Speaker 6: With the $6000 ones do you literally just put it on the eye

Speaker 5: Put it on the eye. Find the easy way.

Speaker 1: [crosstalk 00:21:57] As you get coming with this you follow a red reflex and it auto-focuses and goes click and takes the image.

Speaker 2: It sounds like it's not terrible to be honest.

Speaker 1: It's a bit of a learning profile. It looks like it takes about two hours to learn to do it. So, we're doing it with a C and C and it is for C and C's. And one of them is always on so they're gonna be the people that take the images for all of this. And there's about 20 of those presentations per day at Westmead so that's very doable. I'll tell you at the end of the trial in two months and we'll see how it goes.

In terms of integrating it, is it reasonable to say you all think this is a reasonable idea to get images of the fundus and we've got to try and bring it back in some form.

Speaker 2: Yes, it is.

Speaker 1: In terms of how we bring it through to clinical practise, 'cause that's where it's gonna be. The crux of things. You were talking about having some kind of accreditation. What are the motivators that we get physician trainees and other physicians interested in this.

Speaker 2: You should get them before they go on to physician training. They're always want to have an extra line on their CV to get onto various programmes. They're so keen to learn stuff. [crosstalk 00:23:11].

Speaker 5: But you'll at least get them to do it so their CV's look better now.

Speaker 3: I know, it's true though. Once you pass that they're on, motivation drops. It's true.

Speaker 7: That and the exam. I think they're the two big motivators, aren't they?

Speaker 3: Then the exam. If you then get the colleges to say you have to have at least done this course once and tick it, they'll do it.

Speaker 1: So, if Atlanta, who did this photo ED study which is amazing what they've been doing. That thing, exactly what you suggested, that's what they did in ED for the last four years and it's consistently 10-14% of those patient presentations have pathology and it will always be missed except with the photo they said. But for their medical school, they've stopped particularly, they've dropped down all the time that they spend training you on the direct ophthalmoscope and they instead train you on how to interpret a fundus photo so you can pick a subtle optic disc swelling.

Speaker 6: That would be very useful.

Speaker 1: And because they thought, and I think this is correct, is that the technology's getting better and better. This is where we're at already. We built it for $1.28. People are building the $6000 one ... There's a paper out just last month that they built that $6000 one for $100. And it just clicks on to the back of a smartphone.

Speaker 5: That's a very heavy smartphone.

Speaker 1: So I think within the next ten years you'll just hold your phone up and go click and it'll take a decent photo and interpret it for you and spit the answer out. So, it's coming, we just need the people to do it.

Speaker 6: If you can combine it with an education session at the same time and then it could go, this is the technology. Go and look for the minor changes of papilledema or the changes in diabetes and hypertension. Then people may have more of a buy in and willingness to actually practise because they got a quick refresher with all the residents and fundoscopy pictures that they will look for. Because otherwise they haven't seen a bad retina for two to three years since med school potentially.

Speaker 5: Unfortunately I think in the scope of medicine you need barriers and to make people do a lot of things. Because usually it's always when you come out in the real world, that's when you go, "Oh, actually, crap, I need to learn how this happens.". Maybe not for my specialty but maybe for the endocrine, maybe make an actual course that the registrars have to do.

Speaker 3: What about neurologist registrars. Would most have gone and done extra training?

Speaker 2: No, I think most neurologists are terrible at fundoscopys. In the eye clinic we see lots of referrals from neurologists query papilledema, discs are normal and vice versa. They're just not good at it.

Speaker 3: I think certain sub-specialties in particular. Neurology, endocrine, diabetes, rheumatology, I reckon ...

Speaker 2: For GCA's I think that could help.

Speaker 3: It's the main, yeah, picking up the things specific to your specialty, people would like. So if I had a course looking at hypertendency of diabetic chambers, I think people would quite like doing that. The other area potentially is can you do measurements and things off the photos and then, so to follow people but also people would be interested in doing research and things off this much easier [crosstalk 00:26:15].

Speaker 2: What if you did like an OCT combined with that camera? It's not possible?

Speaker 1: With the $30,000 ones they do.

Speaker 2: Oh really?

Speaker 1: Yeah. So you can get actually for $60,000 there's that auto focusing one that I told you it does everything for you. $60,000, Top Connor can put you in touch with a rep that can trial it for you and see what you think. And they told me that for a department would they push it cheaper. So basically just be pushing them all to give me them for free or cheap. So for about $45K you could have one that does an OCT at the same time. The cool thing at some of the conferences now, they're building OCT's that are the size of a microchip. So you may well be able to have your phone with an extra thing that just clips in to [crosstalk 00:26:57].

Speaker 7: That will be very amazing.

Speaker 1: I'm a glaucoma sub-specialist, so my other bug bear on these things, like [inaudible 00:27:08], 50% of the blinding will come as is completely missed in the community. From some of the big studies like Blue Mountain's Eye Study and things. So as an opportunistic screening thing, if you're getting a photo of the disc, will pick the people or at least a computer will pick for you, the people that have glaucoma and need to be picked up in advance. So in terms of a screening protocol, if we were to say to, it's more for GP's I suppose as a screening protocol rather than physicians. If you say a GP was meant to do a prostate exam and your pick up rate for that, it's a fairly basic examination and the pickup rate for that is like 2-3%. [inaudible 00:27:49] is 4%, about 40. It should have a reasonable pick up rate.

From a physician point of view, is that kind of opportunistic screening, is that part of the plan behind examinations that you're doing or is it more targeted to the presentation that [crosstalk 00:28:09].

Speaker 6: I think it's targeted.

Speaker 7: I think it's becoming more and more targeted.

Speaker 3: Ours have been way more targeted. They should be screened but because of the time it takes to get, especially fundoscopy time, it takes to get and the result that you get where is often sub-optimal. That's why people don't do it. But if it's easy and quick and someone else that's trained in the office, like say in GP practise they've trained one practise nurse to do the role. Everyone walks in the door gets it. You'll pick up a lot of stuff I think.

Speaker 2: I think we'd have to have three potential targets for teaching and one would be at the medical student level and start it there. And then for the physicians you'd want it like Vanessa said, maybe do it in the resident years, like a master class like we were talking about. And then have another master class type thing before the physician exams because that's another time when they're very motivated to learn. But I think if we're gonna do that and if we're going to get the college to agree to think about some of this new technology for the exams, we were thinking maybe instead of just fussing around with the direct ophthalmoscope saying I can't view the disc, that you actually either get a photo to look at that's from one of these devices or you can perform a photo during the exam. Or something like that, we need to speak to them a bit further.

But if we're gonna do that we need to get the old physicians to learn this as well. And so maybe the college would have to also run some education sessions because most of them are resistant to new technology or a lot of them will have no idea what it is and then they'll say it's cheesy like they did with the electronic stethoscope and the panoptic ophthalmoscope. I think it's gonna take some time to get this in but we're gonna have to target really all levels to get it accepted.

Speaker 3: Do you think it would be easier, i mean you're doing this already to show the clinical applications at the end, which is that in ED, when I use this and I've taught these people with a one hour session, we've picked up all of this extra stuff, therefore, we've gotta start training. Because if there's no end corners yet to say well how are we gonna use this, why do they need to learn this skill, they won't agree to do this.

Speaker 1: That was something that the ED guys particularly wanted was flow charts. Okay, someone presents with headache, I have disc swelling or not, what do I do with that information. Because part of ours would be from a neuro point of view, if they have a headache but no disc swelling, and it doesn't sound like a subarachnoid, you may not scan straight away. You might wait and if they need an MRI as an outpatient. You know, when appropriate, they might not sit there for beyond the four hours waiting for their CT scan that they don't need. Or you might consider not doing the lumbar puncture in the ED, in an appropriate patient. Same with haemorrhages at the disc or if it's a hypertensive severe hypertension, if they've got retinal changes then that suggests they've got end organ damage and need admission and treatment. Versus if they don't, they can probably be treated on the spot and go home. So, we don't have that data from the photo ED trial in the US. That's the data I'm trying to get from our, it's Westmead and RPA at the moment that are doing this study. That's what we're trying to get from [crosstalk 00:31:06].

Speaker 4: If this became an accredited skill that was used in the wider medical community, then you would have to link that with a medicare item, I think. So that would encourage people to actually use it. And I'm not talking about hospital practise, I'm talking about practise out there. So, an accredited skill, with the college being involved in some way, and a course, and then a payment for using it.

Speaker 7: That has budget implications though [crosstalk 00:31:45].

Speaker 1: 'Cause they started with that with the telemedicine and it rapidly, 'cause they got on board very early with the telemedicine IDA and then suddenly people were billing it for a lot. Suddenly there's this map. Because I looked at this when we were doing all this, as to how we get people to come on. There's this map that basically says Greater Sydney plus about 50k's where any specialist in Sydney could easily drive to in the day, you're not allowed to use telemedicine. And outside that you are. So I think we will make ... I definitely agree because you see too many practises, a lot of GP's, the financial constraints of their practise is important. That they can pay the salary of the nurse who's gonna take those images or whatever in order to make it.

Speaker 2: But OTC doesn't have a number still and it's used on, basically every ophthalmology patient all have an OTC and we just have to bill the patient at the moment but obviously at the public hospital there's no payment for that so, I think, 'cause these could be basically done on everyone in just about every presentation. I think you'd have huge resistance from the government in trying to get an item number for it.

Speaker 5: I think to get uptake in ED and things you may need to think about coming out with a headache protocol. I don't know if there's an existing ...

Speaker 7: I don't find protocol, I mean ...

Speaker 5: They do. They should [inaudible 00:33:06]. We have back pain protocols, you've gotta kind of, you're low risk, medium risk, high risk. Chest pains and things like that but if they have it I think at least it will make them, especially for the junior doctors, they will actually get them to start going through it.

Speaker 7: But it's part of the cranio-nerve examination. I don't understand why it's been just completely ... I know why, it's too hard. We need to just get it back into ... [crosstalk 00:33:34].

Speaker 5: That's the training.

Speaker 3: Here, med physician goes I'd like to do a fundoscopy at this point if you like, and they always look at you to say don't ask me to do it, okay cool I'll do the field one. [crosstalk 00:33:40].

Speaker 5: Unfortunately I think people are better if their protocol ... if they say, "Here, you just have to follow this and go through this chart of how would you go through a headache.", and they would just go tick, tick, tick, okay.

Speaker 7: But what percentage of ED patients have headache and it's often in the context of an infection or all sorts of other things. I just think it's slightly different to say chest pain when they're either basically cardiac or non-cardiac and I don't know how well it would work. It will be complicated.

Speaker 5: It will be but ...

Speaker 3: I reckon if it's easy like this and we show them and start gradually getting a few key people to use it. You've gotta just get them to realise how easy it is and then have some sort of easy online training for them which is what you're doing.

Speaker 1: So one of the things you said there was the figure of medical students, so interestingly a lot of the papers around the world plus the focus groups I've had of medical students here, so they feel like they're actively discouraged from doing fundoscopy.

Speaker 3: Yes, they are.

Speaker 1: When I spoke to ED about this, they said they weren't really actively discouraging it but maybe unconsciously they were like, "Well, I'm not gonna be able to interpret what you do so, sure go for it. If you like, do it.". What do you guys think about the med students?

Speaker 3: Yeah, they'll say, "I'd like to do ophthalmoscope show, okay, don't worry about it.". [crosstalk 00:34:56].

Speaker 7: Same with the physicians.

Speaker 3: Again, they'll pick it up and say [crosstalk 00:35:00] exam, let's look in your eyes, no, I won't bother. They've got a short time in a long case, if they've got an hour per patient they're not gonna waste ten minutes of it trying to look for the eye when there's nothing.

Speaker 5: Ten minutes is the exam time, physical exam time. Top to toe.

Speaker 3: Yeah, but I'm saying in a long case they wouldn't wanna ...

Speaker 5: Do you do top to toe in ten minutes in a long case?

Speaker 4: In a short case, I think they might in the short cases.

Speaker 5: At least you make up the whole seven minutes.[crosstalk 00:35:23].

Speaker 7: You've seen the [crosstalk 00:35:24] they would do the same thing, don't they?

Speaker 5: But you don't learn to do the exam in seven minutes.

Speaker 3: This person's had decreased visual acuity, do an eye exam.

Speaker 5: Then they have to do [crosstalk 00:35:40].

Speaker 3: They still would ask.

Speaker 6: Actually I had eyes in my exam [crosstalk 00:35:45], real one.

Speaker 5: Did you use your ophthalmoscope?

Speaker 6: Actually, the lead in was you had to start with the eyes, so the cranial nerves two to six ...

Speaker 5: Was it dilated?

Speaker 6: Yeah, I think it was.

Speaker 5: Okay so it actually really [crosstalk 00:35:54].

Speaker 6: [crosstalk 00:35:55] like in Brisbane. And then in my long case I had to look at the eyes again. I was like, [crosstalk 00:35:59].

Speaker 5: How unlucky. How unlucky are you, like it never happens.

Speaker 7: And you were like, "I'm gonna fail" ...

Speaker 6: All in the same day. And the guy has HIV so I had to look [crosstalk 00:36:07].

Speaker 2: Your eyes are not uncommon, I don't think. But they're more likely to be eye movement problems I think in the short cases, then actual [crosstalk 00:36:14].

Speaker 1: So some of the options would be to just say if they ask for the eyes you give them a fundus photo and your interpret it, because there's so many different variations of this, I think the college rightly might say we don't want to accredit this thing because that's a [crosstalk 00:36:32].

Speaker 6: Two things. I think maybe they can do the fundoscopy. But if you just show them the photo it takes away the actual [crosstalk 00:36:39] using the ophthalmoscope.

Speaker 1: The clinical skill.

Speaker 2: But if it's so easy then that doesn't matter. Because that thing was so easy to do so then ...

Speaker 7: Way easier than the other way.

Speaker 2: Maybe they still have to have it because of their long cases and things like that and you can still ask them, but you could potentially have the photo to show them as well. It's better than what we get now. Really we get nothing at the moment. [crosstalk 00:37:00].

Speaker 6: No, we don't.

Speaker 5: Even the smartphone's better than what we get.

Speaker 2: Yeah. And even when you ask them to do it you can see that it's not being done hardly ever.

Speaker 6: Well, everyone just goes through the motions.

Speaker 2: That's right.

Speaker 6: [crosstalk 00:37:13] I can't see anything from an undilated pupil. You can't fail the joint case by saying that.

Speaker 5: Because usually we can't see either, that's the problem.

Speaker 6: At least you tried, so you can't fail them for not trying.

Speaker 2: At least you tried. That's right.

Speaker 4: And in your programme, in all the research you've looked into, in clinical practise say, in North America, which is the thing that is most likely to be used routinely.

Speaker 1: Most valuable is probably these non-redriatic cameras, but they're expensive. Most of the ones that are on big trials are these fixed things, so it's on a tabletop and that has the challenge that it's difficult to get people there. So, interestingly, in those big trials they report very good things. When you speak to the people ... Actually they had a medical student who was doing all of the photos. So someone interested was doing everything. And they tried at an RPA having one of these on the neurology ward but they only got something like four percent of the patients to there because you had to have a nurse and then someone else carrying them around to get to the photo and then someone had pressed the wrong button on the camera. So I'll be able to tell you at the end of our pilot because we're using this portable handheld thing. We're also doing it in diabetics at Westmead and Black Town on the back of that Glen Maberly's [crosstalk 00:38:29] stuff ...

Speaker 4: So there'll be a lot of different ways.

Speaker 1: A lot of different ways. So that's more of a point of care, I was thinking for this and physicians, this maybe a bit more valuable because you'd have it in your pocket. And as you're doing your ward round or whatever you could do things. But by the same token, if you had a neurology ward around and you're going around with a computer on wheels thing now, there's no reason why you can't have the neurology ward round having that on the computer wheels and you get a 45 degree fundus image on every single neurology patient as you go through because ...

Speaker 2: And that would be ideal for physician training because you don't have to buy an ophthalmoscope thing either, which is ...

Speaker 1: Even if that thing is $500, that's less than an ophthalmoscope and you're getting [crosstalk 00:39:09] ...

Speaker 2: Exactly. And it's already better.

Speaker 6: I've gotta go, sorry.

Speaker 1: No, thank you very much guys. Do you mind just briefly filling in one of those?

Speaker 4: Can I have one as well?

Speaker 1: We have some here.

Speaker 4: Thanks.

Speaker 5: We're not supposed to sign something?

Speaker 1: Should I wrap it up there then because I think that covers most. Does anyone have any other big things they've thought about?

Speaker 4: I can't imagine this not going forward as a clinical tool. In whatever guise. It's terrific, really.

Speaker 2: The clinicals would be pretty interested in getting a session. The students would love it. So it would be interesting talking about getting that for them at some point. Or whether they do it through the ... because they get lectures through the eye hospital [crosstalk 00:39:55].

Speaker 1: Yes, I do stuff with them at the eye hospital lectures in the second year and I've done the Scorpios [crosstalk 00:40:01].

Speaker 4: Have we been audio recorded?

Speaker 1: We have been audio recorded.

Speaker 4: Oh, you didn't ask us.

Speaker 1: Sorry.

How did we do?


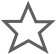

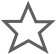

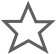

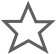

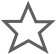


If you rate this transcript 3 or below, this agent will not work on your future orders

[Rate this transcript](https://www.rev.com/transcription/rate/5D99E92136C3BE012FA6CD80D83F708094BF24D86B0DD156E608E096?source=2)
