# Supplementary material for: Optimising fundoscopy practices across the medical spectrum: A focus group study
Source: PLoS One. 2023 Jan 27;18(1):e0280937. doi: 10.1371/journal.pone.0280937 (PMC9882965; doi:10.1371/journal.pone.0280937)
Supplement: S1 Dataset — (ZIP) [file pone.0280937.s003.zip › minimal dataset/GP.docx]

**eFOCUS General Practitioner**

Speaker 1: Okay I'll keep this running in the background. Well the main thing I want to ask you about ... actually I'll start with a story, which is why I got interested in this whole project, and this was a particular presentation. There was a young girl I saw at Sydney Eye Hospital who was an eight year old girl. She had come with a note from her psychiatrist saying 'Please confirm functional vision loss'.

At that time she was hand movements in one eye, and about 6/60 in the other eye, and this girl had presented to her GP, initially, saying she had a little bit of blurry vision and a headache, and she'd had a full neurological exam, was normal at that time, and she was sent home and then she had in a short period of time had lost her uncle and then her cousin. Both died from different causes. And she was quite distressed about that and then a short time later she presented to an emergency department with a headache and a bit of blurry vision and some tingling in her fingers. Had a neuro exam that was reported normal, and was sent home.

They talked about it and this was some bereavement issues. And then she was waiting outside her school, and a kindergarten kid stepped in from of a bus and she reefed this kid off the road and saved the kid's life and put her shoulder out.

And then that night went into the same emergency department and said, "I've got headache, blurry vision and a sore shoulder." They did another neuro exam but not a fundoscopy and said, "Oh this is a conversion syndrome because this is a near death experience and you've just had these other losses."

So she was referred to a psych team. She had only had one other GP presentation in the interim and then another presentation to an emergency department in a different spot. And each of these does have a neurological exam recorded but no one looked at the back of the eye. And then the last one, which just said vision's really bad, and visual acuities recorded these as kind of hand movements and 6/60 and saw the psych team and they said, "No, this is full blown conversion syndrome." And so she just came to us to cross the T's and dot the I's, but she had horrible papilledema, the worst papilledema I've ever seen in my life, and she had a lumbar puncture, she had an opening pressure of 80 and really bad idiopathic intracranial hypertension it was in the end, and permanent loss of vision in both eyes as a result.

So that was kind of the scary case for me where I kind of said, "I'll ... you know ... " but I wanted just your responses about a case like that, is there anything that comes to mind with ...

Speaker 2: I, well obviously with a case like that I would have sent her to a neurologist, but we actually have a neurologist that works with the [inaudible 00:02:35] package, and Jay's the Head of Neurology at Bankstown Hospital and he does one day a week with us and so I would have sent her along, I wouldn't keep pushing for something like that. But I mean, I've got to admit when it comes to fundoscopy, I'm not good, and never have been, but yeah I would have certainly pushed for that. Honestly if we give one of these I'd imagine Jay would use it as well, because he only does fundoscopies on everybody there. Yeah I would have pushed that because a big litigation thing there, isn't it?

Speaker 1: Yeah, absolutely yeah. I think the thing that interested me is the neurological exam, do you think, I mean we're obviously off the record here, how often do you think GP's in general are doing a fundoscopy as part of their neurological exam?

Speaker 2: Very rarely.

Speaker 1: Right.

Speaker 2: Very rarely, because I think they find it technically hard and so it's not done, and it's a skill, it's a skill that you'll want to do very well. But doing it this way with new technology is going to make it easier, but probably wouldn't have. I would say that probably one out of 100 might have done it. Probably most of them would have said, "Oh you need to see a neurologist because of the headaches" and that, but they probably would not have done a fundoscopy, and if they had, whether they would have interpreted it, that's the other thing too.

Speaker 1: Yeah, absolutely yes.

Speaker 2: That's the other thing, particularly a child like that, it's not easy. Hard enough on adults sometimes and doing this on a child ... I think Jay, from memory he did pretty well with his neurological exams and he always does a fundoscopy. You don't know him?

Speaker 1: No.

Speaker 2: You don't know He's the Head of Neurology at Bankstown Hospital. Sorry I couldn't stop it in time.

No, but maybe in the New Year, if you've got the time you can come around one lunch time.

Speaker 1: Yeah, that would be good. Yeah, absolutely.

Speaker 2: At this stage everyone's there, and they all can show it to them, because if you ask them the question how often they do it, they will tell you not very often.

Speaker 1: Across the board that's the answer I'm getting.

Speaker 2: Every surgery is equipped with one, they've got equipped with them there but I don't think they use them and I certainly would be one of the guilty ones too. You're with James, aren't you?

Speaker 1: Yeah, yeah.

Speaker 2: I send a lot of patients to James. And I mean, when you've got someone that's got a problem, you're always pretty reasonable, it's no trouble getting somebody ...

Speaker 1: In ...

Speaker 2: ... that's the good thing, some of the specialists you ring up and they can't see you for two weeks and all this, but then they did and of course it's great having somebody in the room, so if you ever get kicked out of here, you want to come and have a look at the medical centre, because there's over a thousand square metres that can be still used. The club itself has got 70 000 members or more. They advertise you up on their screen, you're going through the pub all the time and a lot of those people think they've got something wrong with macular and that, they're having to go over to Hurstville to see Downey, or Miranda to see him, because he's got time, Downey. You haven't been to his rooms?

Speaker 1: I haven't been to his rooms, no.

Speaker 2: He's got two computer screens up, so he can actually show you-

Speaker 1: Everything as he goes, yeah.

Speaker 2: Yeah, I don't know if you have that sort of thing?

Speaker 1: Yeah, we do in the patient rooms, yeah.

Speaker 2: Yeah, yeah, [crosstalk 00:06:18] which is really, really good. And I often still send a few people to the Emanuel and Gregory. Emanuel's probably mid-seventies now,[inaudible 00:06:26] but he'd been a brilliant specialist. And he's still practising , he still does cataracts and things like that. And he had to it the hard way, because I mean he came out from Sri Lanka, fully qualified eye specialist, and they made you do the exam and everything again-

Speaker 1: Yeah, and then when they have to convert [crosstalk 00:06:50] ..

Speaker 2: And he ended up with a teaching position at New South Wales Uni. Yeah, well I gotta admit, I put my hand up, I'd be one of the guilty ones, I'd probably really, really under do that part of the examination.

Speaker 1: Is the availability of the specialists one of the things that motivates that, do you think? I just, so if it was technically easier like this, do you think people would do it or are other things gonna get in the way-

Speaker 2: No, no they would do it. Because everybody uses computers and I-phones and I-pods and, of course it is. It doesn't actually give you an interpretation, does it?

Speaker 1: No, no. Not at the moment. That's coming.

Speaker 2: Because that's going to be the ideal thing. Because one thing's doing it and the other thing's interpreting what [crosstalk 00:07:33]

Speaker 1: What you see.

Speaker 2: Yeah. But I mean, at the moment you can do something, you can say to the patient, "Well this is what it looks like, to me it looks okay." But something would go like that, even if I'd thought that, I'd still be sending him to you to get it looked at.

Speaker 1: Yeah, sure.

Speaker 2: We've found [inaudible 00:07:52] specialists. The specialists have found it good too, because they're running just down the corridor. You've got a patient, you run them down half a way to [inaudible 00:08:00]. Mind you, while they see him, and you can come up and they can change [inaudible 00:08:07]. ...

We've got 20 000 patients, that's a lot of patients.

Speaker 1: Yeah, wow. Yeah.

Speaker 2: And I imagine, here, eventually, all these places will get knocked down.

Speaker 1: Absolutely, yeah. [inaudible 00:08:16]

Speaker 2: Yeah, the [inaudible 00:08:19] in the process now, of leasing out the rest of it. If James is ever interested, you should come maybe, [crosstalk 00:08:28]

Speaker 1: I think they're looking around, yeah. I think they're actually looking.

Speaker 2: Are they? You should come down and have a look. Maybe in the new year. January, February. Come down [inaudible 00:08:35] and I can show you and maybe you can get back to James.

Speaker 1: Yeah.

Speaker 2: It's got parking for 480 cars.

Speaker 1: Right.

Speaker 2: Well, it has. See, we've got Carl's underneath us and we're on top of Carl's. But the way the land's sloped, you're actually coming in to the ground level. You're not actually having to go upstairs or anything. There's a lift that'll take you down to Carl. And under that, there's two levels of parking so there's parking for 480 [inaudible 00:08:59]. Cause, there's all other different shops, there's sushi shops and chicken shops and all these sort of things and... Cause the club just won the award for the best effort ever for health care for any club in Australia.

Speaker 1: Oh, right?

Speaker 2: It's 40 million dollars in putting this whole complex up.

Speaker 1: Wow, yeah.

Speaker 2: That's good. But, we actually this year, we won the silver award for the best medical centre. Then we won the gold award for the best business.

Speaker 1: Wow! Cheers. That's amazing.

Speaker 2: Yeah. [inaudible 00:09:35] cardiologists here. We've got [inaudible 00:09:39]. You know Michael Carr, [crosstalk 00:09:39].

Speaker 1: Yeah, I've heard of them, yeah.

Speaker 2: Yeah, well Michael and Andrew are with us. [inaudible 00:09:44] our cardiologist. We've got Mohammed Hassini, podiatrician. Mohammed is with us. Unfortunately, his mom just died. She lived out [inaudible 00:09:54],who I have [inaudible 00:09:56]he was over there for about two weeks, he was sure she was okay. Was back here one day, and she died. So he had to go back over again. [crosstalk 00:10:04] ...

Yeah, really. So we've got podiatry, psychologists, the whole lot. But, would love to have an eye specialist, because the people have to travel a far while and the club's good enough that they'll use their bus and keep them in the area. They'll pick them up and bring them over for consultations and if they can drive themselves there's no problem parking. It's very disappointing that doctors don't come to these things.

Speaker 1: Yeah. What do you think of the other things, so that, I mean, if we made it technically easier for them to do these things, what other things do you think ... I mean we were building an online training system in how to interpret the [inaudible 00:10:48] so you kind of see [inaudible 00:10:48] what it is to accredit you and how to interpret the back of the eye.

Speaker 2: That would be great.

Speaker 1: Now, and we were looking at a kind of master class format of kind of you know, doing an evening session, dinner and you do training and how you use this stuff and how do you interpret what you find. I'm hoping that would get ...

Speaker 2: You saw the interest you had that night. You probably want to have a look at it and have a go at that. I mean, they would probably be more interested in the mom with [inaudible 00:11:17] they'll probably bore for six thousand dollars.

Speaker 1: Yeah

Speaker 2: Yeah, if you've gotta be in that practise, it's not a bad [crosstalk 00:11:20]

Speaker 1: Yeah, a lot of doctors using, or a lot of practise nurses using it and a lot of doctors interpreting it.

Speaker 2: Medicine is not all about making money. I mean, there's some things that you're better to spend the money and have really good equipment and stuff and give people a good service. You know, you just can't be based on getting a fee. It's probably the way the government loves to hear you talk, but still, I don't agree with that aspect of it, personally.

Speaker 1: Yeah. Is there a comparable thing, I mean ... One of the things, it's a similar skill to otoscopy, which you guys are fantastic at and you're doing it all the time in kids and things that are coming through, but what do you think is the difference with kind of, you know that looking at the ear as the prime of scope.

Speaker 2: The difference between what?

Speaker 1: Well, just, you know, looking in the ear everyday base kind of pretty comfortable ...

Speaker 2: Every child I see, I look at their ear. Every adult that comes in that's got a cold, I have a look at their ear to see if they've got some sort of eustachian tube disfunction. So you're really doing that all of the time, you know. Where, I probably should do it more often, but you're probably only gonna get one or two people or three people a week that you really need to do [inaudible 00:12:39] on. I found that really handy demanascope, we've been using that, and that's made a big difference. We do a lot myops stuff. I mean, I think this sort of thing is good that people can store it on the record. But, I think we need training. Because its no good being able to do and not know what you're doing ...

Speaker 1: Not knowing what you're looking at. Yeah.

Speaker 2: And, you know, we didn't really get much ... I'm nearly 70 now, but when I went through, you didn't get much training on eyes. No real skill in using a funduscope. And of course they were the old fashioned funduscope. I think all the doctors have got to be very interested ... and as you said, you saw the response you had.

Speaker 1: Yeah, I saw the response in that. Yeah, seem to be quite a lot of interest in that.

Speaker 2: I don't know why I didn't come today.

Speaker 1: Yeah, I think you're absolutely right. I think a lunch time thing that's not on site for them is the wrong way to do it.

Speaker 2: Yeah, [inaudible 00:13:38] I don't know. I just know what they like. I mean, most screenings you have with a GP division, 50% of those who accept don't turn up and half the people that turn up that night didn't even accept, they just decided they'll turn up. Really hard to plan it.

Speaker 1: To nail it down, yeah.

Speaker 2: I think it's cool. We actually go through Vital. And when you gave the talk, I asked Vital about these, they said they didn't have these available yet.

Speaker 1: True.

Speaker 2: Both of these ones are TJ approved and available in Australia.

Speaker 1: Yeah, but Vital didn't ... Vital is just a medical supplier, were you guys [crosstalk 00:14:16]

Speaker 2: Oh, no so I don't think the suppliers had them, I could send you the details for when you need them.

Speaker 1: Okay, cause I will definitely get one of these.

Speaker 2: Well, Have you got a pan optic in your department? Do you have one of the pan optics in your clinic? Like this one.

Speaker 1: Yeah, yeah. Yeah, we have one of those.

Speaker 2: Yeah, okay. Well at Wershallen, for that, that's a 60 dollar add on, so you just, and it works on an I-phone 6 or I-phone 4, it doesn't work with any of the other ones, so it is kind of different, straight on.

Speaker 1: I think we will do that. Maybe give me [crosstalk 00:14:59]

Speaker 2: Yeah, I'll give you the details and I'm building this master class should go ahead in kind of March or something next year. So I'll send through to the division when it comes.

Speaker 1: We've got [inaudible 00:15:06] down the road from us. We've got quite a few GP's in the area, we won't be able to sort of tell [crosstalk 00:15:16]

Speaker 2: Yeah, I know that's the [inaudible 00:15:17]

Speaker 1: You can try to get 10 or 15 people there.

Speaker 2: Yeah.

Speaker 1: Not all that much [inaudible 00:15:23] in this area. But, yeah, let's do that. Cause, it's a shame. You're doing something to make our life a bit more easy. And, now they're both at [inaudible 00:15:42]. Understand that.

Speaker 2: Yeah, all these happen. The other things, some of the things medical students said, and I want to mention this to some other GP groups that were teams here ... but the medical students said that they were worried because it's a face to face thing, and you know, coming up close to someones face is a confronting thing to do. Do you think that's a medical student thing, or? [crosstalk 00:16:08]

Speaker 1: What? No.

What do they have to worry about? Suddenly they're gonna pop off on the patient under them or something or getting cursed? ...

Speaker 2: Yeah, so they said things like they were aware that they weren't too [inaudible 00:16:20] and that they were aware that the patient had to be looking kind of close to them and that they were a long time close to the patient's face. So the discomfort factor was - [crosstalk 00:16:27]

Speaker 1: You explain it to the patient. You know, this is a technique that I gotta get close to you, it takes a little bit more time. Now, if you've got something like this, you can actually show them the picture of what you've taken. But I can understand [inaudible 00:16:48]. See a lot of students don't want to do general practise now, they want to specialise and have the opportunity to be specialists.

Speaker 2: Yeah, not enough GP's, yeah.

Speaker 1: Yeah, you know, we're doing quite well. And the opportunity we've taken is the club wanted this for people. They wanted something special for them and that's what we've gone to the trouble of providing ... I'll get Karen, our practise manager to give you a ring and find out what lunch time is suitable for you.

Speaker 2: Yeah, absolutely.

Speaker 1: And, I'll let a few of the other doctors in the area know that you're gonna be there.

Speaker 2: Yeah, that would be fantastic.

Speaker 1: And, they're gonna get 10 or 12 people there.

Speaker 2: That sounds good.

Speaker 1: And they're happy with all this sort of stuff. They all like sushi and that sort of thing. And that's easy, there's a sushi shop down the block.

Speaker 2: Right.

Speaker 1: And Carl's. Yeah.

Speaker 2: In our beach town, remember, back then they had master classes in dermatology. And, when I had masterclasses in this sort of thing, you can get a certificate that says, you know, that I have done a course. Maybe I've done 20 or 30 funduscopies, and I've reported on these ... and I can't see a problem.

Speaker 1: What tends to work best with them? Cause we've gotta fund the patients coming through and things. Do people normally pay something when they go to those master classes with [inaudible 00:18:19] accreditation that comes with it? Or do they kind of, support it by ... [inaudible 00:18:23]

Speaker 2: No, no. They pay something for it. I mean, they got the high to sort of say that they're skin specialists. But they're not ... no they're happy to pay. But they usually do, they grade them. They might have a lower one, medium and upper one. So people can do the lower one. If they find it interesting, you can include more things in the next one. And then, you're still not going to be an eye specialist, right?

I think all doctors want to feel more competent. My days of training, it was good, but you just got [inaudible 00:19:02] when you're at the Bankstown Hospital, obviously, as the senior resident, no one would come in. You had to handle it. No matter who you rang up, they all over at Sydney, however, we can't come in till tomorrow morning. You've gotta handle this patient, you've got to do it. Be how you want, from experience.

No. I think they'd do it. And I think they would be very interested in doing it. I mean, the dermatology ones have it taught through the observer and the doctor's newsletter, I think. But no, I think they would.

Speaker 1: And the motivation for learning this ... I mean dermatology, I suppose they're doing all the time, but like almost all GP's, you're going to be seeing a lot of skin stuff coming through.

Speaker 2: Well, I do a lot. And we're lucky now, we have Icon across the road. It's an oncology/radiotherapy, and we've got a lot of patients and older patients with skin cancers on their knees and those, which is gonna be really debilitating if they're operated on, then we can send them over to them.

I don't know that you learn all that more than you did, but you get that satisfaction. But this is important too. This is probably more important, because there's doctors that are gonna be very poor at it.

Speaker 1: And getting them to come in ... I mean, some of the things the other GP's were saying is you know, the things that motivate doctors are going to be the things that get them into it, like you know, some people are motivated by picking up a rare diagnoses in things, or some people are motivated by doing enough screening that you will catch the 1 in 100 cases of something coming through. Do you think that changes people's or GP's motivation?

Speaker 2: I think it does. I mean, casually can be pretty bad sometimes. I mean, they have a look a lot ... I just had a lady recently that went in because she was feeling weak and she had some PR bleeding. She had a haemoglobin of 87. So they did a sigmoidoscopy. First time, they didn't find anything. Second time they did the sigmoidoscopy, allegedly found some haemorrhoids, which they banded. But she had no further bleeding. But after giving her iron and everything. Infusion. Came home, I did haemoglobin on her, it was 80. That was two days after she was discharged. I did no other tests. First thing, cause she was reluctant to have a colonoscopy, I sent her up for a CT scan. Leaking aortic aneurism. Being operated on, on the 16th. Anthony Freedman is doing it. That's bit of a [inaudible 00:21:40].

Speaker 1: Wow.

Speaker 2: 60 centimetres.

Speaker 1: Geez. That's amazing. I didn't even know they could get that big.

Speaker 2: They don't. She's lucky, that's [inaudible 00:21:50]

Speaker 1: That's unbelievable.

Speaker 2: Yeah. Usually they're around the 40-45, now, cause they stent it now. They don't cut them out like they used to. Yeah, that's where the bleeding was coming from, the aortic aneurism was leaking.

No, I think it's great of you ... Do you want me to sign one of these things?

Speaker 1: Yeah, do you mind? Does that kind of cover ... I mean that kind of covers from my aspect the kind of questions I was thinking about how do we integrate this into GP. So, my things are archioplstics and glaucoma so from a glaucoma aspect, if people are incidentally looking at the news as well as other things as part of a screening test ... the only other question I would have was, say for example, a prostate exam ... The catchment for a prostate exam is what, two to four [inaudible 00:22:33] in or something and the [crosstalk 00:22:37]

Speaker 2: Yeah, I guarantee most doctors don't even put their finger in there. We get PSA's, and it's interpreting that as PSA's ... My best man, when I got married, he sees me as a patient now, but he didn't til about four years ago. He said, "I won't see you cause I'm frightened you'll find something wrong with me and I would never want you to have that responsibility." And he's a doctor, retired. He came down and he's due for his blood tests. And I did some blood tests on him. And he's PSA out of the three years before, so me being rising, it's still in the normal range for him, 2.3 to 2.9 and it was about 3.3 when I saw him, so, I said to him, "I want you to get an MRI done." It showed some suspicious areas, which are subsequently biopsies, at worst, cancer.

Yeah, most doctors ... And there's talks like that all the time. I mean anytime we ever get a talk on anything to do with eyes, it's with James and you. And James is a little bit like a locomotive, he goes too fast.

Speaker 1: Yeah. He's a powerhouse.

Speaker 2: Well he is, but you can only take so much in. And he tries to cover a really big area. It's better to concentrate on ...

Speaker 1: A couple of things and get them right.

Speaker 2: Yeah, that's right. There was a huge interest in that. Maybe they thought you were going to be selling them today, so they didn't come. I don't know. I don't know. As I said, I was chairman of the GP division for when it started, for 10 years, and we always had this kind of trouble with people attending.

I would go out ... At that price, they've all got [inaudible 00:24:27], so we'd just buy one for every room.

Speaker 1: Yeah, well the thing that's coming ... so I'm working with a biomedical engineer and he's building one of these things for $1.28. So it's just going to become ... yeah, it should be about pretty easy, pretty soon.

Anyway, I'll shut off the recording and we can go have a bite to eat.
