# Supplementary material for: Optimising fundoscopy practices across the medical spectrum: A focus group study
Source: PLoS One. 2023 Jan 27;18(1):e0280937. doi: 10.1371/journal.pone.0280937 (PMC9882965; doi:10.1371/journal.pone.0280937)
Supplement: S1 Dataset — (ZIP) [file pone.0280937.s003.zip › minimal dataset/Medical Student - Final Year.docx]

Facilitator 1: Basically the purpose of this is to help fill in the gap with the e-learning stuff that we're putting online. And to just get a better impression of what are the ways you guys learn clinical skills, and how you go about implementing them with patients, and about funduscopic in particular. So nothing, your involvement, or not. Doesn't impact on your senior medical school curriculum with any of the people involved in the study or anything like that, so you're free to be involved, or not as you like. I want to actually, at the end, grab your emails and I'll just send you the consent form, because I've left it in my car.

And for the purposes of just the recording, I'll get you to just say your name and if you're happy to be involved in the study? Any of the things we use will be de-identified, so your names won't be involved. We might put a gender and an age in there, in publication, but otherwise nothing to identify you and nothing to identify any patients or anything that you talk about. We'll probably go for about 40 minutes or as long as people can handle going for. If you need to go, let us know.

You all met me before I think, so Facilitator 1. You know Facilitator 2 then, and I want to hand over to Facilitator 2....

Facilitator 2: Yeah. Can we just runaround and get your names? Get everyone's names here?

Facilitator 2: Okay. So Hamish is the expert on ophthalmology. I know nothing about ophthalmology except it must have two eyes. But we've done lots of focus group research over the years. A focus group is a good technique for fleshing out a new research area. There's some stuff written about why people are shy about using fundoscopy and, and looking at patients' eyes.[inaudible 00:02:35] It's kind of a bit superficial, it doesn't dig into the detail of why people might be concerned about looking at a patient, an NAD or if you're a GP, or wherever. So what you would do... is we actually get your words, your thoughts about it, and then you turn it into more objective data by writing down on a questionnaire, some questions about, you know, I find fundoscopic and I never know which end is which, and that might be [inaudible 00:03:07] and then you ask people do you agree with this on a five-point scale, and then you develop an objective measure.

Then you can give that to lots more people, and get a sense of how common those sorts of feelings are. So what you're doing is fleshing out the detail and then turning it into an instrument for collecting more objective data. That's where we're at, at the moment. So there's no right or wrong answers to this, as I say, with a focus group you're interested in the words, you're not interested in who generated the words, so I'm sorry, if you say something brilliant, it's going to be lost in the ether. I mean I want you to be honest about, you know you were dying for ophthalmology training, you may find it the most boring subject ever, but that's not [inaudible 00:03:47]. So just to list generally about looking in peoples' eyes, and the way you've learned to do that.

F3: I find it intimidating because I'll, when I look in someone's eye, I might think it looks terrible, but then I'm told it's normal. And so I ... And so when I'm looking in the ophthalmoscope, if I can see everything, I can't then, you know, take a photo, stop and point to things, and ask about them. It's really difficult to get feedback. So I think that's ... I don't ... I've really struggled to understand what's normal and what's abnormal, because the spectrum of normal seems so large to me.

F2: Especially if they've had treatment for stuff before, and you're looking at it like "What?" And it's like, "Oh, they had laser."

F3: Yeah.

M1: It's the same thing, you can't be taught, because the person doesn't know what you're seeing. The person who's teaching doesn't know what you're actually seeing down the microscope. Or the ophthalmoscope. Like, if you've got a good view of the optic disc then maybe they can tell you about it, but a lot of the time when you're starting I don't think we can even get that far?

F3: Yeah.

M2: Two things. One, the times when I've tried it and I've just blatantly failed. And you know, it's that moment where do I keep wasting, do I feel like I'm wasting time just pursuing something that I'm not really going to know if I'm seeing anything or any pathology, I don't even know if I'd recognise it, or do I just spend more time on the things that I'm more confident I'll get.

F2: I think also just not being very good at it, and I think even though we have our ophthalog book, and we go and try to do as many as possible, it's still like not, again, a natural action? So sometimes I feel like the same thing like, when you've totally failed, and you can't see the back of their eyeball. YOu're just kind of like. Well we'll star the patient. Like if you're going to be able to keep your eye open for a limited amount of time, why don't you do it for the person that knows what they're doing?

F3: Yeah, that's what I feel like. I don't like just shining a light in their eye and it's uncomfortable for them.

M2: So far as I can tell, really nobody looks. If we do it and we've got a supervisor standing on top of us, that would be you saying why bother?

M1: Right.

M2: You know, it's like you don't look into it.

F3: Even when I do the endocrinology, and they're doing like a diabetic assessment.

F1: Yeah.

F3: They're like, "Oh, no, no, we don't do that. We just send them to the optometrist. Or the ophthalmologist, sorry.

Facilitator 2: So what's the difference, say, you've talked about not being able to see anything really, so why waste your time and why waste the patient's time? Plus inconvenience to the patient, discomfort for the patient. I guess if you ...if you're taking a blood pressure, or listening to a heart, maybe it's taking a blood pressure, the cuff might be uncomfortable for a patient, so other interventions can be uncomfortable, and it may be difficult for you to hear heart sounds early in your training. Can you just talk a little bit about is opthal...looking at the eye different from those sorts of investigations?

M2: I think in the acute setting, then we can reliably get a blood pressure, it might not be 100% accurate, but pretty accurate, and if needs be we can intervene or we can call for people to intervene quickly, whereas looking into somebody's eye, I'm not going to be able to really intervene if it's anything serious. We're still going to call for help.

F1: [crosstalk 00:07:39] won't necessarily even know that what you're looking at is a bad thing. Like sure, blood pressure might be a bit inconvenient to a patient, but at the end of the day, you know you're going to get an answer. And listening to a heart, that's not that difficult, as in you put the stethoscope on and it's not that uncomfortable for the patient, you know, it's not a big-

F3: I think the eye is very sensitive. Some people just don't like having things over their eyes. But also like compounding that with the fact that we don't feel like, well I don't feel like I'm going to do a good job. And then we don't see other consultants doing it, and so it's like, well they're not going to do it, I don't know what I'm doing, I don't know what I'm looking at, so ...

M2: Well, without the modules we know the parameters, and we-

F3: What should be normal.

M2: We know immediately what's outside of that, whereas for the eyes, unless we keep doing those modules and we can recognise it quickly.

F3: Yeah, like you were saying, like it's hard to know what's normal, like with heart sounds you know what sounds normal and you know what sounds abnormal, you might not be able to tell what murmur it is and you know, explain it very well, but at least you can say, "Oh, that doesn't seem right," and then get further input.

F2: Because I feel like I know like the words, I'm thinking like ... "Ah, yes, cotton wool exudates, and there's some AV nipping/nicking," and so you know like in theory, but "Ah yes, the cup-to-disc ratio," and then when you're actually there looking at it, you're like, "I don't see any of these thing!"

F3: I had neuro surg say once he sucked at fundoscopy and so when he thinks that clinically the head raise, RCP he just says to his bosses, "Oh yeah, papilledema," like he really can't see. Which is really bad.

Facilitator 2: Just following on a bit more, the notion of difficult to pick up what's important, what's wrong. So when you first listen to heart sounds and you didn't hear things, do you remember the same sensation of I'm on a learning spectrum here and then I'll get there, or was there an experience of, "I can't get this, I don't understand it"?

M1: I found that with heart sounds, there's like a culture where you know at some point you'll have to know or you'll be called up on it. Whereas I feel like [inaudible 00:09:57] ophthalmology, it might not. But like I will need to learn how to look for papilledema, but why would I need to look for diabetic retinopathy when I'm going to send someone with risk factors to someone who knows it better regardless of what I see?

M2: With every single patient, we'll listen to the heart sounds, and every doctor from the intern up to the consultant is going to listen to the same thing and discuss it.

M1: Yeah.

M2: Whereas, I haven't seen the same thing with ophthalm.

Facilitator 2: Do you just want to talk about that case that you wrote in your project overview?

Facilitator 1: So one of the reasons I got interested in this was the case of a young girl I saw at CVI Hospital who was a six-year-old, and she'd come to us via six presentations to a couple other emergency departments, and the first of those was she'd had a couple of, two family members die, in short succession, and then she started developing headaches and was complaining of blurred vision. She presented to the emergency department and had a neurological examination, which was reported normal. They sent her home.

Things got worse. She presented another two times, and then each time she had a neurological examination, which was reported normal, and the vision level was just declining slightly on each time. And then she was standing after school in the bus line and one of the kindergarten girls stepped out in front of the bus in front of her and she pulled this girl off the road and hurt her shoulder doing that. Saved this little girl's life.

Hurt her shoulder doing that, presented to an emergency department for the fourth time, and with a sore shoulder and tingly fingers, and progressively-worsening vision. She said it was getting a bit darker, she couldn't see. Had another neurological examination, which was reported normal and then they got the psyche team involved, said this is a conversion syndrome and you had multiple losses and so you're getting functional vision symptoms.

And then she was sent to us for confirmation of functional visual loss. She had the world's worst discs I have ever seen. It was, you know, you could look in there and it was haemorrhages everywhere, massive disc swelling, and on none of the five occasions when she'd seen the emergency department had anyone done fundoscopy even though she was complaining of those things. And we know from other research elsewhere that it's just not done because people don't feel comfortable doing it.

So I suppose one of the things we're interested in is that thing that you were saying, like early on with the heart sounds, you don't quite know what you're interpreting, but you would press on knowing someone's going to do it, and with eyes, I suppose, you're not getting that feedback of whether what you're getting is correct or not. The other interesting thing that you were saying on finishing is if you think they might have this, you might be tempted to say the kinds of things that you know technically might be there. Have you had other clinical scenarios like that, where you think, "Oh, this is what I'm supposed to be hearing, or supposed to be seeing"?

F4: Heart sounds.

Facilitator 1: "Am I tempted to kind of say it's...

F4: Like if you say on ECHO report you know they have like aortic stenosis.

F1: Yeah.

F4: You're going to say ejection systolic [inaudible 00:13:18], regardless of what you hear, like.

F1: [inaudible 00:13:22]

F4: Like something in or around those heart sounds, and they look like someone that maybe has aortic stenosis, then you might also be like, oh ejection systolic murmur. Well done!" And they really don't or you're not exactly sure. So yeah.

Facilitator 2: My reaction to that story that Henry's told me was allied to when I was studying diabetes. I did my [inaudible 00:13:44] in diabetes and you'd often see somebody would come in with all kinds of retinopathy because of their diabetes, and if the ophthalmologist had looked at their eyes and said, "Your eyes are really shit, and you really need to think about managing your diabetes better," that might be the turning point, it might be truly intervening and the patient will start to take it seriously, whereas in the past they haven't.

And I know some brilliant diagnosticians will pick something up that other people have missed. So it's kind of like, you have a whole panorama of stuff you can do, listen to hearts, whatever, that are routine, and if you actually saw somebody like this girl, and you were the [inaudible 00:14:29] well, let's just have a quick look in the eye, "Oh, yeah," and you go, "Hey hey, look what I know, that other people missed!" So it's kind of like you've got a tool in your toolkit you don't use very often, but if you need to, you'd be enormously pleased with yourself if you actually used it at the right time.

F4: Yeah.

Facilitator 2: Is there anything like that, that might actually change the way ophthalmologists teach students, to make them think, "Okay, this is something I won't use very often, but it might be worth knowing how to do it." [inaudible 00:14:56].

F2: Does the thing exist? When I was on rural placement, one of the GPs had an otoscope that was connected to a screen, so I could see what he was seeing, and that was so helpful for learning that. Like, does that exist for an ophthalmoscope? Because that would be how...that if we could actually see what they were looking at, in order to kind of get a better appreciation of-

M2: I thought the smart phone devices were very good and would make me more comfortable to do it, knowing that I don't have to in terrible, terrible ophthalm language try to describe what I'm seeing, I can just go, "Listen, I'm not sure, [crosstalk 00:15:36]" send a photo to the REG and they can say "Yes" or "No," as opposed to [crosstalk 00:15:44] I think, and for me to even look at it in slow time.

F4: Yeah.

M2: Whereas you know, just getting a quick glimpse in, I'm not sure what I'm looking at, I'm not sure that I could go through the checklist that you were making us do the other day, which-

F4: Or like you can, but like number one, what are you looking at? number two, [inaudible 00:16:06] yeah, that's what I had to do.

M2: Yeah.

F4: Yeah, but I think it's also more than just ophthalm, like because we don't ever see anyone else doing it, it's so easy to just not do it. And so like you know, if we were in ED, then it seems like if I look in, I'm not going to see anything, but I also know that others probably aren't going to and see it as important. And so that, I know that disconnect and it's about, I know you can't really change this but the culture of fundoscopy doesn't really encourage us to be good at it and I don't know to get...

F4: I think it would be good if we had like, you know, conditions, not ... Like a more heavy focus on conditions that we shouldn't miss.

F4: Yeah.

F4: So like, retinal haemorrhages in children, and papilledema, and stuff like that, that we could focus on really knowing those skills pretty well, rather than just like knowing-

F2: Vaguely everything, yeah. Because I think they do a really good job of drilling it into us in PEDS, like red reflex, red reflex, red reflex, and so-

F4: Yeah.

F2: PEDS exam, red reflex, cool. But beyond that, just kind of ...

Facilitator 2: What do you think about M2's suggestion, that if you had the camera on your phone and you just send it to the ophthalmologist registrar and say, "I don't know what this means, what do you think?"

F2: Yeah.

Facilitator 2: "Do you want to come down and audit?" It actually gets you past the point of having to say, "I've seen something."

F2: Yeah.

F4: Yeah.

Facilitator 2: Like at ISPAR. The ISPAR is about your recommendation, "I'd like you to come down because I think...this," but what you're saying is you hear people say, "I have no idea what I'm looking at, but does this look okay to you?"

F2: How do you take a picture? Is it just like, hold your phone up to the [inaudible 00:17:57]

F4: Yeah. Like with your smart.... With you're iPhone? And it just has like a little thing that goes over here-

F2: Yeah.

F4: -like it's about that big, and then you put it against the eye, and a picture of it comes up.

F4: What?

F2: Yeah. I feel like that would be-that's sweet.

F4: And you can film it and so then you can go back and look again.

F2: All right. Well, that's really going to be a game-changer.

F4: Yeah. I'd say.

M2: That would be for you.

F2: Yeah. Absolutely.

Facilitator 2: So what is it about that would be?

F2: Because I feel it's more like I've got a record of it, so I don't have to try to describe what it is, like I can show somebody-

F4: Proof!

F2: I can also see, I feel like you'd have more control of like seeing if you're in the right spot and getting a good picture rather than ...

M1: And it adheres to what is commonly done elsewhere in the hospital.

M2: Yeah.

M1: So, especially in ED, before the registrar comes down, you will take a picture and ask them do you want us to deal with this or do you want to intervene?

Facilitator 1: So is that a common thing that you're kind of nodding to that now... across other fields, you'll take a picture of something else and send them to [inaudible 00:19:02] and show that to them?

M2: It's usually for surgical consults and...

F4: [inaudible 00:19:06] X-rays, I think [inaudible 00:19:08]

F2: I did a derm rotation and it was constantly just like other people sending, this was in Canada, like an elective, but we'd just get tonnes of pictures, like, "Do you want to come see this or can you tell by the picture?" And like they'd kind of pick, which consults they would do kind of more urgently or not, based on ...

Facilitator 1: So one of the things, obviously getting completely off the record, the ... Were most of those just personals someone would send from their phone to the REG's phone direct?

F2: Yeah. Definitely not the whole [crosstalk 00:19:37]

F4: Yeah, and I think there should be definitely rules surrounding that. Like I would say when you take the photo you try not to have the patient's vitae and details like that in. But still, you've got stuff on your phone that shouldn't be on your phone.

Facilitator 1: Yeah.

F4: It's like in an ideal world, though, there'd be like work phones, like work iPods or something.

F4: I think they should get rid of pagers, and just give you phones.

F4: Yeah, me too. Like they have VOIP phones in orange, but I don't know how you can upgrade from just like the ...

Facilitator 2: Yeah. I think by the time you guys are out there, there'll be legislation around that that makes it much more easy for you to take photos and deal with them as you need to. Because it's so logical, you know.

Facilitator 1: So is part of that, again getting the consult and that kind of thing, does having the photo reduce the, change the barrier to you, you know, looking for intel? You know, if you're looking at an X-ray and you don't know how you would interpret that previously, if you know you can get immediate feedback from someone, does that change your likelihood of looking at it, change your likelihood of doing it, or change your likelihood of looking into how to interpret it? Or what do you think?

F4: I'd be more likely to do it, knowing that like, you know, it doesn't rest with my interpretation, because like that's the thing that's been difficult with fundoscopy is that if you look, it rests on your interpretation.

Facilitator 1: Right.

F4: But if you've got some sort of record, then you can look at it and think, "Uh, that looks weird," and even if you don't know if it is weird, or you know, it's got some condition, then you can rely on someone who actually knows what they're talking about. So I'd be more likely to do things, knowing that you know...

Facilitator 2: It's an efficient use of resources, isn't it?

Facilitator 1: That's kind of what happens in rural medicine.

F4: Yeah.

Facilitator 1: You have GPs and other people who don't have the specialist skills to check a retina. And it's been fairly routine, I guess. Is there a different attitude because you have all the resources [inaudible 00:21:42] is there a different attitude towards using sort of "exotic" interventions, than you would see in the country, where you know you don't have those interventions, so you want to actually ask somebody else's opinion?

M2: I don't know. But I do like the idea that I can have instead of just a glimpse over a second, that I can bring up a picture and then go through an algorithm that I can, or a series of steps, to analyse what I'm looking at, in the same way that when you give me an ECG, I'm just going to think, you know, it's one step at a time. Right? [inaudible 00:22:20] and so I'd happily have your algorithm of ... What are those questions? And if I had the picture, I could answer them one by one.

F4: Yeah. I'd love that too.

Facilitator 1: M1, you've been quiet.

M1: I think you'd also create the expectation you'd do it more if the ophthalmologist like was expecting to get them more frequently. Say, in like dermatology, you might leave things alone, but if you could just easily send it to someone, you might do it more.

F4: I wonder if people are sending photos and stuff to all the different REG's, poor things...does that just completely stuff up their workload and mean that everything is really interjected, you know, so like they're with the patient on ward rounds and they're just constantly getting messages about like, "Oh, look at this person's fundoscopy" You know, I don't know, I guess I'm saying is it practical? I don't know if anyone knows.

F2: What if it didn't get sent directly to the OP REG, like you would send it to your REG first? I mean like, because say you are doing...

F4: Yeah.

F2: [inaudible 00:23:30] like you're in ED, and you're an intern, and you talk to your person that you're reporting to.

F4: Yeah.

F2: Hopefully they'll know, but if they don't, then it goes. Like it could be like an ascending system.

F4: Yeah, yeah, that would be probably more effective.

Facilitator 1: Somewhere you have got to let the registrar in the hospital, the person whose job it is, is to farm out responsibilities, so you're actually saving them having to stop what they're doing, come down and look at the patient, look at the eye, and so on and so on.

F4: That's true.

Facilitator 1: If you [crosstalk 00:23:53]

F2: Like in triage.

F4: Yeah.

F2: [crosstalk 00:23:54]

Facilitator 1: It is like that. So I am the senior REG and I'm normally getting photos from ED or ward consults all the time for externalised stuff and that stuff and you just say send me a photo.

F4: Yeah.

Facilitator 1: Because you can look at a photo and triage in two seconds. I kind of need to see this in the next 30 minutes," or, "I'll see that in the next three days."

F4: Yeah. Okay, so it's not-

Facilitator 1: So the same thing with the fundus. You can quickly, you know, as opposed to going, "Okay, I've got 10 phone consults with no information about the disc," now I've got a bunch of photos that I can go, "Okay, 30 minutes-"

F2: So it is more effective.

Facilitator 1: "Today, next week."

F4: But as M1's saying, I think if it was an expected thing, like you don't, you know, like with visual acuity, you don't call the ophthalmology REG until, we shouldn't! Until you've done a visual acuity.

Facilitator 1: [inaudible 00:24:35]

F4: I've been told, I've heard ED people talk about it, it's like they'll just call the ophthalmology REG and have him really assess the patient. If they, you know, if it was part of that expectation that you had to have looked at the disc and taken a photo, perhaps, then I think it would become standard.

Facilitator 1: One of the things, just jumping back to something you said, Claire, which was that if you could show someone the photo, and you knew it wasn't resting just on your shoulders to do the examination, you might be more likely to do the exam? Just that point of deciding to the exam, does anyone else feel that you're kind of less-inclined to do an exam if it's going to... the responsibility for interpreting that is going to fall to you without backup?

F2: Well, yeah, because you don't want to be the one that like, threw like the spanner in the works by saying, "Oh, it's fine!"

F4: Yeah, I wouldn't be comfortable saying, "Oh yeah, it's fine."

F2: Yeah, I feel like it's just fine but if I thought there was something odd about it, because then I would ask somebody, but if I looked at it, and honestly thought it was negative, and then ...

F4: And then it wasn't.

F2: And then it wasn't, so by not having been the one that does it, you hope that somebody's going to take that risk and know for sure.

Facilitator 1: So that point, do you think then you would prefer not to do it at that point? At some clinical point where you go "do I not do the examination?"

F4: Oh, yeah, I would only do it if like they were [crosstalk 00:26:03]

F2: [crosstalk 00:26:03] if it was super relevant, like if they came in because, you know, [crosstalk 00:26:06]

F4: Headaches.

F2: Headaches and visual field disturbances or something, then I'd be like, "Yeah, okay, I'm going to do it." But then either way I'm going to get somebody to double-check.

F4: Yeah, I'd do it if it was clinically really important, but then I wouldn't document that it's normal, because I wouldn't be confident that I knew it was normal.

Facilitator 1: Right.

F4: Yeah.

Facilitator 2: Would you be happy to document, "looked at the eye, and could not see anything."

F4: Yeah, in my limited experience.

Facilitator 2: [inaudible 00:26:36]

F4: Nothing, no abnormalities detected by the intern, who's done this four times!

Facilitator 1: If you compare that to say a heart, so say you know, most patients come through, you put heart sounds, a murmur or not on a routine exam for something unrelated? Do you feel is that a different thing when you're kind of saying that on a patient? Like you think the heart's not necessarily related to their condition, do you go through that same thought process about do I feel comfortable doing this exam and giving a result in the notes? Is that the same process?

M1: I guess its the same. You think that with a cardiac condition you listen to the heart for a lot longer, whereas if [inaudible 00:27:14] heart sound [inaudible 00:27:18] a quick look at it.

F4: Some people just write, "Heart sounds [inaudible 00:27:23]." Well, if they're not sure, like we were in ICU and we had quite a big guy, and so it was actually just hard enough to hear his heart sounds, so you couldn't really tell whether there was a murmur, so they just wrote, "Heart sounds [inaudible 00:27:36]." But ... I wouldn't write something if I didn't actually know. Feel confident that it was like, it was not there or it was there.

F4: Yeah, so by that, meaning that you would have more confidence in not hearing the murmur, than you would in looking at a funduscope and being like, "Ah, that's normal."

F4: Yeah, yeah.

Facilitator 1: And those things change all the time on a public health level. You think about PSA screening. Only a couple year ago, everybody above a certain age was screened, every male was screened for PSA, and now they dropped that screening back because there are so many cases where it over diagnosed, so false positive rate. Do you think about it in those same terms of screening? You know, like if you were in clinic and you'd see a patient, you're screening for things that are likely to be there, and you're returning with your user screen on the basis of how likely it is you're going to resolve, you can interpret. Or is that a different sort of thinking?

M1: I think it's scarier to use the screen because then if I say it's okay, then, like because with screening you want everything rolled in kind of thing.

F4: Really sensitive.

M1: Yeah. Whereas some other things might look normal to me, or if I don't see it I might be concerned about ...Say I just look, don't even see the disc in a patient without serious needs, check the disc. I might not bother my registrar higher-ups to look.

M2: This might be abrogating our responsibilities but in almost all the other screening tests we do, there's a quantifiable value, which is more, and so far I don't see the same in ophthalm. It's just you go through the checklist, do they have this or do they have that? Then I'm not good at recognising whether they're there or not, which makes it more difficult.

F2: It's like the sensitivity of that is a screening test. It's way too user-dependent.

Facilitator 1: Okay. Okay.

F2: So that's why I wouldn't ...

Facilitator 2: Another issue is the fact that when you're looking at somebody's eye, you're right up close. Is that an issue you're aware of?

F2: I think it's something you're conscious of while doing it, but like I'm always like, "Oh no, should have brushed my teeth after lunch!" But I think it's one of those things that like, it goes both ways. It is what it is. It's like do we examine the lymph nodes it's like, no one's going to enjoy this, but we're going to do it.

Facilitator 2: So your feeling is you're aware of it.

F2: Yeah.

Facilitator 2: And as a kind of like, yeah, I'll just get on with it?

F2: Yeah, but it wouldn't, I don't think you would necessarily be a reason that I wouldn't do it or that I wouldn't spend as long in their face unless there was some-

F4: What if the patient was really smelly?

F2: But even like, I feel like it's not something that would prevent me from doing it or ... from doing it as thoroughly as I could? But it's just when you're kind of consciously there.

Facilitator 2: Consciously. And F4, you nod vigorously, what's your feeling about kind of intimacy of doing something?

F3: Well, I just remember when we were doing it on each other. Yeah, same thing, it was like, "Oh, I don't want to sneeze on them," or something like that. Like even when people were doing it to you, you felt like, "Oh, this is very," like not too close for comfort, but it was like, Oh, it's very ...[crosstalk 00:30:59] Yeah, yeah.

F1: But yeah, I'm the same as Kate, I wouldn't mind, like in the clinical situation, where it was necessary.

F2: Plus I feel like, maybe if I wore a mask, it might feel better doing it. Because like ...

M2: Just the familiarity with it, because it's nowhere near the most invasive procedure that we would normally have to do regularly, and the first couple of times we have to do them, we feel uncomfortable, and then after a while, it's just normal.

F2: I honestly think I'd be more comfortable, like if you were to right now give me the option of go do a speculum exam and a pap smear, or do direct ophthalmol, I'd probably take the pap smear.

F3: Me too.

Facilitator 1: Claire, what's your thoughts about the intimacy of an eye exam?

F1: I'm really the same, I think. It would not, I don't think it's too bad, you know, at the end of the day, they're kind of off to the side, anyway, I don't know if you... and it wouldn't stop me from, that wouldn't be a reason to not do it.

Facilitator 1: Okay. But you are aware of it too?

F1: Oh yeah, it's hard not to be aware of it when you're like that close to each other. But it's not, I'm not bothered by it.

Facilitator 1: What's your thoughts?

M1: I think the only thing is like, choosing whether to dilate someone's eye and knowing they can't then drive home or whatever. Like, a lot of the time, like [inaudible 00:32:24] it's really hard to see things. So then you think, is it worth ... But if they had a head injury I'd do it. But sometimes you might be like, "I should really dilate them, but then if there's not much a chance of there being something and that would inconvenience them.

Facilitator 1: I guess the experience we all have of a similar thing is going to the dentist.

M1: Yeah.

Facilitator 1: The dentist's got their face in, got their fingers in your mouth. What's your reaction on the receiving end of that. Is that, does that have a similar sort of feel of this is quite intimate?

F4: They have a mask.

F2: They wear a mask.

F1: Yeah.

Facilitator 1: They wear a mask? Okay.

F2: If my dentist weren't wearing a mask, I would be super grossed out.

F1: Me too.

F3: Me, too. And you can't really see them because they come from behind you.

F1: Oh, you can, but you just see their arms. You don't see their face, as much.

F3: But also, they're like on top, and so like everything's going to fall down. [crosstalk 00:33:23] Yeah, everything! So-

F1: I think it's probably a similar level of uncomfortableness.

Facilitator 1: What, as a recipient?

F1: Oh, no, no, not like in terms of comparing it to fundoscopy and being in a dentist's chair.

F3: Because yeah, I'd be more uncomfortable if they didn't wear a mask.

F1: Yeah.

F3: Because [inaudible 00:33:45]

Facilitator 1: M2? What's you're experience of sitting in the dentist's chair?

M2: Oh. I'm pretty nonplussed about it.

F3: That's how nice your dentist is.

Facilitator 1: Would a mask make it better for you, or ... ?

M2: [crosstalk 00:33:55] It's just the norm.

Facilitator 1: Especially if he was not doing it. Years ago, dentists never wore masks.

F2: I'm really, I need to go. I'm overdue for the dentist by about four years, right now.

Facilitator 1: Pretty much everybody I speak to is overdue for the dentist.

F2: I hate the dentist.

Facilitator 1: So there are two continuum we've talked about there. One is the intimacy, and the second is the inconvenience or the discomfort. On a... if you imagine a scale where we've got, this is absolutely no discomfort to the patient, that's maximal discomfort, presumably something like a lumbar puncture is right up there? In your experience, what's the top of the scale, the most-

F2: PR exam?

Facilitator 1: PR exam, yeah. I'm thinking in your routine examination or investigation, rather than something exotic, surgical intervention or-

F2: Yeah, I guess catheterization?

Facilitator 1: PR exam?

F2: Yeah, a catheter going in, like ...

F1: Yeah, pap smear or vaginal examination.

Facilitator 1: Okay, so and they are closely related to intimacy aren't they. So can you think of situations where the intimacy and the discomfort are not related?

F1: Just like painful things.

F3: Well, a lumbar puncture isn't really intimacy thing, I think it's just the painful thing.

Facilitator 1: It's just the pain.

F3: Not that I've had a lumbar puncture before, but I can imagine.

Facilitator 1: So I'm just trying to, by asking the question, get a feel for the balance of intimacy and discomfort, because you talked about dilating eyes and that's uncomfortable, and the fact that you might be physically close to a patient might be uncomfortable for them, might be uncomfortable for you. So just starting from the patient's perspective, what's the balance between intimacy and discomfort, from the patient's perspective, that you think is a barrier to eye examination?

M1: I find with those intimate stages if the person who's doing it to you is confident, it's not a big deal.

F4: Yeah.

M1: But because we're not confident and it's intimate you can kind of feel that they know you don't know what you're doing, yet you're still shining a torch in their eye.

F3: Yeah, you're going to spend longer doing it.

F1: Yeah, I feel like it's more user-dependent. Like the intimacy thing, you know, if you're confident and you're in quick and you're out quick, then it's not as bad as if you're, yeah, awkward and yeah, go for the wrong eye, that sort of thing!

F2: Because if you talk your way through, one of the other things, if you're like doing a pap smear or something, ....I just came from colonoscopy clinic. So, it's just like you're telling them, "Okay, now I'm going to put the speculum in, now I am going to open, you're going to feel a little bit of touch when I," you know, as I'm taking the brushing and you're like walking your way through it, but I feel like if I was doing, because I'd be like, now I'm going to awkwardly approach you just like this. I you're having to explain what you're doing it's more of like trying to fill the time ....

Facilitator 1: Okay.

F2: Maybe if there was like a script we could run from?

F4: A script!

Facilitator 1: That's actually a good idea, just nobody's come up with that before. Yeah, explaining what you're doing.

F2: Like, "Now I'm shining in a little more of this ... "

Facilitator 1: I remember my GP where I was used to have a script, "I'm going to give you this injection now, and it's going to feel a bit like this," like I looked at [inaudible 00:37:28] You know, really experienced people do have a script, you're right. Do you think that, that's missing from your experience of your training within the ophthalmological .. A script that accompanies it?

F2: Yeah, because everybody that does it or teaches it, they're so good at it that they don't need a script or that they do it so quickly that it doesn't require a script but then we're standing there for like 40 seconds, breathing on someone's cheek.

F3: It would be useful to say, you know, it's going to be a bright light, make sure they look at something on the wall, and try and ...

F2: Yeah, maybe like if there's always a happy face sticker on the wall behind ... ?

Facilitator 1: I remember when I went and looked at Hamish's ophthalmology clinics when he was based in West Maine and I came in and visited. There was a big screen of somebody with gross retinopathy, and that was the thing you'd look at, while you were having your eyes dilated. "This is good, this is ..."

Facilitator 2: The worst one was the laser room where was there the picture of the person with the chemical burn opposite!

Facilitator 1: Oh yes, that's right. Yes.

Facilitator 2: Poor choice of ...[crosstalk 00:38:30] at the horribly disfigured people while we shoot lasers at you ...

Facilitator 1: Is there anything ... we have had quite a bit of your time.

Facilitator 2: Yeah.

Facilitator 1: Is there anything, any nuances to how you feel about looking in people's eyes, that we haven't addressed at all?

F1: Just that I don't know what I'm doing.

Facilitator 1: Yeah.

F1: Or I don't know what is normal, because I think that everything that's not normal, that's the only ... That's the biggest issue for me.

F3: I think practise opportunities as well. Like MC, we listen to everyone's chest, which, you know, because we're doing it so often we get better at it. But we don't, apart from doing the ophthalm clinics, depending on ... I know some people that do them like, all in a row, and then don't do it for a long time. They don't think about spacing them out, so yeah, I think if we, if it was just kind of the standard thing where you could have a look in someone's eye on a ward round, and yeah, just get more practise.

Facilitator 2: There's all this stuff on the web, just [inaudible 00:39:35] because I know nothing about ophthalmology but he asked my help in this. I just went on the web and there's a lot of photographs of retinas.

F1: Yeah.

Facilitator 2: So you could actually do it by going through those. There are teaching modules that have come from the US and the UK that are actually very good.

F1: And that's what I would do in ophthalm clinic. I'd would look at an eye, and I think I could sort of describe what I saw, so then I'd get on Google and look up all the images of what I thought it would be, and try to go, "Oh yeah, that's what it looked like!" So we do use those things ...

Facilitator 2: Why did you do that?

F1: To confirm what I thought I saw?

Facilitator 2: Okay. And so the question is, why doesn't every student do that? Because presumably they don't all do that.

F2: I think part of it is I'm not as really getting that good view, right away. Like you don't necessarily get the full, like everything you want to see. You don't try to follow the vessels, what can you, you can't quite angle it, you can't quite see the disc, and then ...

Facilitator 2: Is there a ... In most things, there's a tipping point. And just the way you spoke, I thought maybe there's a tipping point involved when you think will come. If you go past the tipping point, you think, "Look, it's not important, the consultants don't do it, nobody else does it, so I won't bother." Or they do what you did, which, "I actually can't understand this picture here so I'll google it." And the tipping point is, "I want to understand this, I think it's important enough for me to understand it." So there's something different about the way you just described it, something that tipped you over to actually go and look up Google.

F2: See, because you're sitting in an ophthalm clinic. You wanted to know what it was.

F1: Yeah! What it was, yeah.

F2: Because you want to know. It's not that like you're looking at it and you don't...

F3: And were probably going to be asked what I saw!

M2: So, we don't get many opportunity to using the funduscope, so we've played around with them a couple of times, but when we do the ophthalm clinics ...

F1: Yeah, they will just use a slit lamp and so...

M2: And on wards, nobody uses a funduscope.

Facilitator 2: Yeah.

M2: So we're being asked to a piece of equipment that nobody, or we hadn't seen anybody use, really.

F2: And yet I still feel more comfortable with it than the slit lamp.

F1: And then, no, the slit lamp thing, they use that whatever it is, lens thing, and that's even harder. One ophthalmologist tried to make me use it and I went "Oh, I have no idea, this is even worse than fundoscopy.

Facilitator 2: The slit lamp? Okay.

F1: Or the using, the what is called?

Facilitator 2: The lens, yeah.

F1: The lens with the slit lamp because that's how you see the retina, with the slit lamp. So yeah, that was hard.

Facilitator 2: Just the last thing I wanted to ask is, having...the last time I went to my ophthalmologist, who was a guy I went to school with, he said to me, he came out with my file and said, "I see you're back for your 25-year checkup." He sits there, looking through his slit lamp, and then he talks to me around the side, and it's almost like the mechanics of the setup lead you to look at the eye, and it so disconnects you from the patient. It actually creates, there's something physical between you and the patient that drops that intimacy that you guys were talking about. Is that...does that make any sense to you?

F2: As in, you saying that the slit lamp would help?

Facilitator 2: I guess I'm wondering why have the ophthalmologists not gotten more teaching time into the curriculum and getting you guys exposed to fundoscopy. Is it something about their practise? I mean only need line up to probably think about how that machinery between you and the patient actually affects that intimacy, which is still very close.

M1: Yeah, there's a bit of distance. I suppose the question for those who have done that...the smart phone ones, is did you find the intimacy different using the smart phone version?

F3: Yeah. It was less because your face wasn't all the way up in there. You just held it there and looked and looked at the screen.

M1: Okay.

F3: I thought it was less.

F1: Yeah, definitely less.

M1: You did it M2, did you?

M2: Yeah.

M1: With the iPhone? And do you find ...

F3: Oh, an iPhone.

M2: I found it was much, much easier to use. It was quicker to acquire a good picture and you could look at it afterwards.

Facilitator 2: So, just to summarise some of the things of ...back in the corner...is there a bit of a spectrum in how likely you are to do any investigation with the patient based on how intimate it is with them, how much discomfort the patient is going to be in with it and how confident you feel with being able to get an answer to your examination and then alongside that some kind of feeling of being able to interpret what you've got and feedback from your superiors along with that? That seems to be the kind of decision making to going into it and then behind that is the culture of, or around that is the culture of whether it's expected that you do it or not or be able to ....

F1: But I think also is the patient presentation and how important it is to do fundoscopy and will it change the management? I think that's probably one of the most important things than your skill level, than patient discomfort. And then intimacy is [crosstalk 00:45:04]

F2: Intimacy is not something that really I consider at all.

Facilitator 2: So, the patient presentation, if you think of the patient presentations where they have come in with [inaudible 00:45:14] like you were saying the endocrine department, presenting, like coming for the diabetic check and the fundus changes are the first thing you see in diabetic complications and they are usually quite good at telling or getting people to start changing things because you can show them the picture and go "here it is. It's bad", some days....

F1: I think if you were an endocrinologist and you didn't have access to an ophthalmology clinic like they do at North Shore you definitely would....like if you were an endocrinologist that went out to the country you would definitely do it. But because we've done it in endocrinology here, they don't do it because they've got, oh, they've got their ophthalmology appointment in two weeks or something like that.

Facilitator 2: Right.

F1: So, it's not that it's not indicated, it's that someone is [crosstalk 00:46:02] ...

Facilitator 2: Someone else to do it.

F1: Yeah, someone is going to do the job.

M1: And just about [inaudible 00:46:06] I feel like [inaudible 00:46:08] hasn't learned stuff like the diabetic things which we probably don't need to know, whereas the things we do need to know like is it papilledema, like just looking at the optic disc every single time will be better than looking at the one patient whose got diabetic retinopathy and not seeing it. But I'd like every opthalmolo...like if I worked with the clinic and I got to look in every patient's eye while it's dilated just to find the disc I think that would probably be better than...

F2: Sitting there, trying to find some with the slit lamp.

F1: Yeah, like your checklist. Just go through that for every patient regardless of what they have.

F2: And it would be more useful to go through a checklist for fundoscopy for like 20 people and do that than it probably is to sit in the clinic and try get your medical records signed off and it's never going to get signed off.

F3: The other thing I is the OCT's because then you can look at it and get a bit of education and discuss these findings. Like I think [inaudible 00:47:15] teaching.

Facilitator 1: Good. Thank you very much. [crosstalk 00:47:23].

Facilitator 2: I will email something with the consent form and if you don't mind just signing it and send me a photo of that.
